# Lesion orientation of O^4^-alkylthymidine influences replication by human DNA polymerase η†
†Electronic supplementary information (ESI) available: Materials, experimental procedures, compound characterization, and additional discussion. The atomic coordinates and structure factors (codes 5DLF, 5DLG, 5DQG, 5DQH and 5DQI) have been deposited in the Protein Data Bank (http://www.wwpdb.org/). See DOI: 10.1039/c6sc00666c


**DOI:** 10.1039/c6sc00666c

**Published:** 2016-04-26

**Authors:** D. K. O'Flaherty, A. Patra, Y. Su, F. P. Guengerich, M. Egli, C. J. Wilds

**Affiliations:** a Department of Chemistry and Biochemistry , Concordia University , Montréal , Québec H4B1R6 , Canada . Email: chris.wilds@concordia.ca; b Department of Biochemistry , Vanderbilt Institute of Chemical Biology , Center for Structural Biology , School of Medicine , Vanderbilt University , Nashville , Tennessee 37232 , USA . Email: martin.egli@vanderbilt.edu

## Abstract

DNA lesions that elude repair may undergo translesion synthesis catalyzed by Y-family DNA polymerases. O^4^-Alkylthymidines, persistent adducts that can result from carcinogenic agents, may be encountered by DNA polymerases. The influence of lesion orientation around the C4–O^4^ bond on processing by human DNA polymerase η (hPol η) was studied for oligonucleotides containing O^4^-methylthymidine (O^4^MedT), O^4^-ethylthymidine (O^4^EtdT), and analogs restricting the O^4^-methylene group in an *anti*-orientation. Primer extension assays revealed that the O^4^-alkyl orientation influences hPol η bypass. Crystal structures of hPol η·DNA·dNTP ternary complexes with O^4^MedT or O^4^EtdT in the template strand showed the nucleobase of the former lodged near the ceiling of the active site, with the *syn*-O^4^-methyl group engaged in extensive hydrophobic interactions. This unique arrangement for O^4^-methylthymidine with hPol η, inaccessible for the other analogs due to steric/conformational restriction, is consistent with differences observed for nucleotide incorporation and supports the concept that lesion conformation influences extension across DNA damage. Together, these results provide mechanistic insights on the mutagenicity of O^4^MedT and O^4^EtdT when acted upon by hPol η.

## Abstract 2

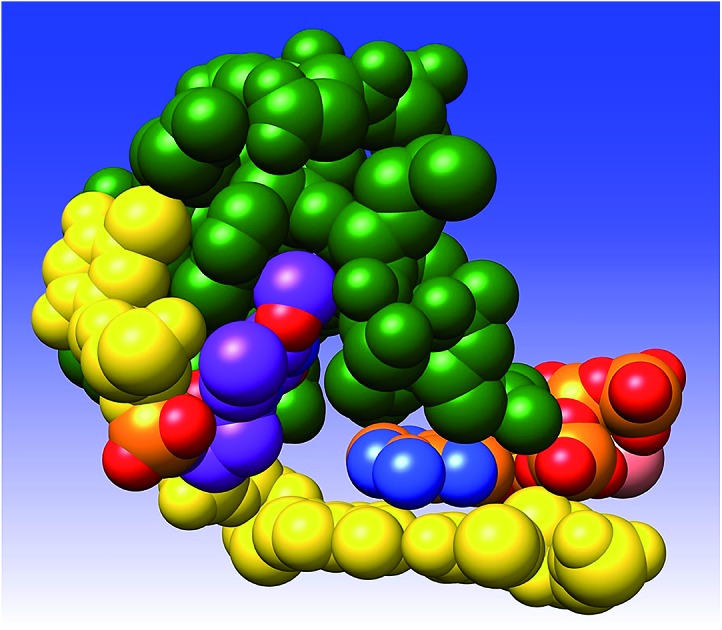
Conformation of the α-carbon of O^4^-alkylthymidine was shown to exert an influence on human DNA polymerase η (hPol η) bypass. Crystal structures of hPol η·DNA·dNTP ternary complexes reveal a unique conformation adopted by O^4^-methylthymidine, where the nucleobase resides nestled at the active site ceiling where hydrogen-bonding with the incoming nucleotide is prevented.

## Introduction

DNA alkylation results from a variety of endogenous and/or exogenous agents that can interfere with vital cellular processes, *i.e.* replication and transcription.^1^ The addition of alkyl appendages on the DNA scaffold can have adverse consequences such as DNA polymerase (Pol) blockage, nucleotide misincorporation, chromosomal instability, and activation of the cellular apoptotic pathway.^1^,^2^ However, organisms have various repair pathways to restore damaged DNA. In the event that a lesion evades the process of DNA repair, translesion synthesis (TLS) by Y-family DNA Pols can occur, allowing bypass of the DNA lesion in an error-free or error-prone manner.^3^ Y-family DNA Pols are described as more “promiscuous” given their larger active sites when compared with replicative DNA Pols, which accounts for their ability to bypass damaged nucleotides that induce blockage. DNA Pol η in humans (hPol η) plays a pivotal role in the bypass of certain UV-induced DNA damage, which impedes DNA replication.^4^ hPol η activity has also been correlated with chemotherapeutic resistance to platinum-based agents such as cisplatin and the efficient bypass of the oxidative DNA lesion 7,8-dihydro-8-oxo-2′-deoxyguanosine.^5^,^6^

The O^4^-position of thymidine is susceptible to alkylation by agents such as *N*-nitroso alkylamines in certain foods, water, air, and particularly tobacco products.^7^,^8^ Albeit a minor site of alkylation, lesions such as O^4^-methylthymidine (O^4^MedT) and O^4^-ethylthymidine (O^4^EtdT) are poorly processed by mammalian repair pathways, making them persistent in the genome.^9^,^10^ O^4^MedT and O^4^EtdT hinder high fidelity replicative DNA Pol activity, resulting in misinsertion of dGTP in the daughter DNA strands.^10^–^12^ Correlations between the mutagenicity of O^4^MedT and cancer have been established,^13^,^14^ highlighting the importance of investigating the structural properties and biological outcomes associated with this type of DNA damage.

**Fig. 1:**
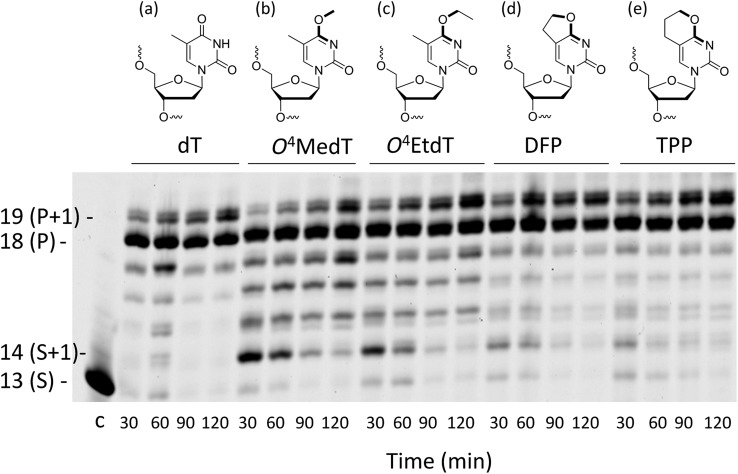
Structures of the (a) unmodified dT control, (b) O^4^MedT, (c) O^4^EtdT, (d) DFP and (e) TPP adducts. In bold are the bonds between the atoms shown to have a *syn* (b and c) or *anti* (d and e) orientation around the O^4^–C4 bond. Full primer extension assays for the control undamaged substrate (dT), O^4^MedT, O^4^EtdT, DFP, and TPP-bearing substrates with hPol η. The template strand sequence is 3′-AGCATTCGCAGTAXTACT-5′ where X denotes the modification and the 5′-FAM labeled primer strand sequence is 5′-TCGTAAGCGUCAT-3′.

The current understanding of the mechanism of Y-family DNA Pol misincorporation during TLS depends on a number of factors, including the nature of the DNA damage, the DNA Pol and the incoming nucleoside triphosphate. The geometrical array of the ternary complex formed (involving DNA, protein and nucleoside triphosphate) is the key characteristic that governs efficient bypass of a DNA lesion. Structural investigation by NMR and X-ray crystallography of duplexes containing an O^4^MedT insert has revealed that the methyl group preferentially adopts a *syn* conformation around the C4–O^4^ bond (Fig. 1).^15^,^16^ We hypothesized that the conformation of the O^4^-alkyl lesion could affect the base pair geometry during the primer extension reaction catalyzed by DNA Pol η. To address this possibility, we probed hPol η processivity with thymidine analogs that link the C5 and O^4^ atoms by a dimethylene or trimethylene group, which limits the O^4^-lesion to adopt an *anti*-conformation (Fig. 1) to relate the structural features of O^4^-alkylated dT with the bypass activity of hPol η. hPol η was selected as the model Y-family DNA polymerase, given previous studies concerning bypass of O^4^MedT and O^4^EtdT.^17^,^18^ Results of these studies indicated that Pol η, from yeast or human, were most efficient in extending across and past O^4^MedT^17^ and O^4^EtdT,^18^ respectively.

We investigated bypass profiles opposite all four lesions by hPol η (steady-state single nucleotide incorporation and LC-MS/MS analysis of full-length extension products). Crystal structures of ternary hPol η·DNA·dATP and hPol η·DNA·dGTP with template strands containing O^4^MedT or O^4^EtdT reveal a distinct orientation of the former lesion that stacks atop a tryptophan residue near the ceiling of the active site instead of pairing with the incoming nucleotide. Conversely, O^4^EtdT pairs with both incoming dA and dG nucleotides *via* bifurcated H-bonds in the insertion complexes and displays the same configuration opposite primer dG in the crystal structure of an extension complex adjacent to the nascent dG:dCTP pair. The structures provide a better understanding of the different behavior of the O^4^MedT or O^4^EtdT lesions in hPol η-catalyzed error-prone bypass reactions and suggests a unique intermediate step in the bypass of O^4^MedT.

## Results

### Synthesis and characterization of modified oligonucleotides

The structures of O^4^MedT, O^4^EtdT and the modified pyrimidyl nucleosides 3-(2′-deoxypentofuranosyl)-5,6-dihydrofuro[2,3-*d*]pyrimidin-2(3*H*)-one (DFP) and 3-(2′-deoxypentofuranosyl)-3,5,6,7-tetrahydro-2*H*-pyrano[2,3-*d*]pyrimidin-2-one (TPP) are shown in Fig. 1 (methods describing the preparation of nucleosides and oligonucleotides can be found in the ESI†). UV thermal denaturation studies of duplexes containing single inserts of the DFP or TPP modification revealed a comparable destabilizing effect to O^4^MedT and O^4^EtdT with complementary strands containing adenine or any mismatched base pairing partner (Fig. S38†). Circular dichroism spectra of duplexes containing the DFP or TPP inserts revealed little deviation from a B-form structure (see Fig. S39†).

### Steady-state kinetics

**Fig. 2:**
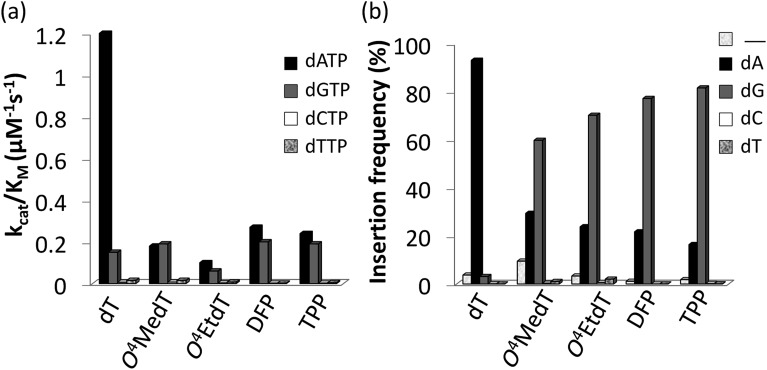
(a) Steady-state incorporation efficiencies opposite dT, O^4^MedT, O^4^EtdT, DFP, and TPP by hPol η with individual dNTPs. (b) Incorporation frequencies based on ESI-MS/MS analysis of primer extension products opposite the dT, O^4^MedT, O^4^EtdT, DPF, and TPP containing templates (“_” indicates frameshift adduct formation. Tabulated kinetic parameters and fragments identified by LC-MS/MS analysis of full-length extension products can be found in ESI†).

Steady-state kinetic assays of individual nucleotide incorporations opposite O^4^MedT, O^4^EtdT, DFP, TPP and unmodified dT were carried out with the catalytic core construct of hPol η (amino acids 1-432). In all cases, these pyrimidyl modifications blocked DNA synthesis by the polymerase relative to the unmodified control (Fig. 2a, values shown in Table S1†). Incorporation of the correct dAMP nucleotide by hPol η opposite to O^4^MedT, O^4^EtdT, DFP, and TPP was reduced approximately 6.5-, 12-, 4.5-, and 5-fold, respectively, relative to dT (see Fig. 2a).

hPol η incorporated dCMP and dTMP opposite all the pyrimidyl modifications and dT with similar catalytic efficiencies (ranging from approximately 0.002–0.015 μM^–1^ s^–1^). However, a strong preference for either dAMP or dGMP incorporation opposite the modified pyrimidines was observed. Other than O^4^MedT, hPol η preferentially incorporated the correct dAMP nucleotide opposite all the pyrimidyl modifications. The significant incorporation of dGMP when hPol η encountered these pyrimidyl modifications, compared to the unmodified control, indicates a clear loss in substrate specificity by the polymerase (Fig. 2a). In the case of O^4^MedT, dGMP was slightly preferred as the nucleotide incorporated by hPol η (0.19 ± 0.01 *vs.* 0.18 ± 0.03 μM^–1^ s^–1^ for dGMP and dAMP, respectively).

### LC-MS/MS analysis of full-length extension products produced by hPol η

Analysis of single insertions by a DNA polymerase is useful for kinetic evaluation but may not reflect incorporation fidelity in the presence of all four dNTPs across the damage and beyond this site. The fidelity of hPol η and its processivity past the damage site was addressed by the use of a full extension assay coupled with LC-MS/MS analysis.^5^,^19^,^20^

The optimal reaction times to observe the full extension products from the template strands containing the modifications and the unmodified control were evaluated (Fig. 1). Full extension was achieved for the unmodified control at 30 min, whereas templates containing the modifications required longer reaction times (60–90 min). The time course assay revealed that hPol η had difficulty in extending past O^4^MedT and O^4^EtdT and displayed a significant “S + 1” band at reaction times of 30 and 60 min. UPLC separation of the cleaved products and mass spectrometry analysis of their sequence identities revealed that dGMP was incorporated most efficiently opposite all the modifications (see Fig. 2b).

The incorporation frequency opposite dT, O^4^MedT, O^4^EtdT, DFP, and TPP for the full extension products was evaluated (see Fig. 2b and Table S2†). The presence of O^4^MedT increased the level of frameshift formation by hPol η relative to the control (9.5 *vs.* 3.5%). Comparable levels of frameshifts were observed opposite O^4^EtdT and the dT control. However, the templates containing the bicyclic pyrimidine adducts did not induce a similar increase in frameshift formation with levels that were approximately one-half, relative to the control. The correct dAMP nucleotide was incorporated by hPol η at a frequency of approximately 30, 24, 22, 16, and 93% opposite O^4^MedT, O^4^EtdT, DFP, TPP, and dT, respectively. Out of the lesions investigated, hPol η exhibited the highest fidelity opposite O^4^MedT and lowest opposite the TPP. Incorporation of dGMP was observed to occur in the extension products with overall frequencies of 60, 70, 72, 82, 3% opposite O^4^MedT, O^4^EtdT, DFP, TPP, and dT, respectively.

The accuracy of bypass varied for the O^4^-alkylthymidine modifications by approximately 2 : 1 in favor of dGMP opposite O^4^MedT to 5 : 1 in favor of dGMP opposite TPP. An increased adduct size, from O^4^MedT to O^4^EtdT and DFP to TPP, resulted in a 10% increase of dGMP misinsertion at the expense of a 10% decrease of the correct dAMP incorporation. Similarly, the conformationally restrained analogues (DFP and TPP) induced an increase in dGMP misinsertion (10%) by hPol η compared to O^4^MedT and O^4^EtdT, respectively.

### Pre-steady-state kinetics

The pre-steady-state kinetic assays of dATP and dGTP incorporations opposite O^4^MedT, O^4^EtdT, DFP, and TPP, and dATP incorporation opposite unmodified dT were carried out with the catalytic core of hPol η. The burst rates for dATP insertion were 3.1-, 4.2-, and 1.8-fold higher compared to dGTP opposite O^4^MedT, DFP, and TPP, respectively (Fig. S48 and Table S3†). The burst rates were low in the case of dATP and dGTP opposite O^4^EtdT. The burst amplitudes for the extensions were 15–35% opposite O^4^MedT, O^4^EtdT, DPF, and TPP, which may indicate the presence of multiple non-productive ternary complexes.

### Crystal structures of ternary hPol η·DNA·dNTP complexes with templates containing O^4^MedT or O^4^EtdT at the insertion stage

**Fig. 3:**
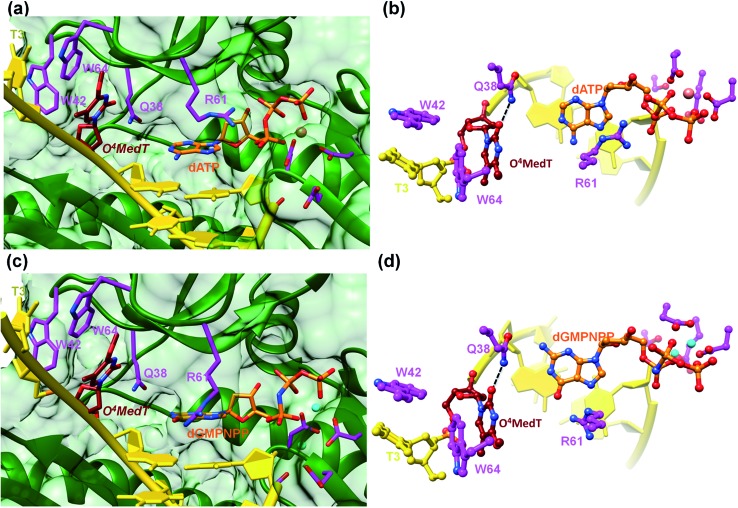
Detached arrangement of incoming purine nucleotide triphosphate and O^4^MedT in two hPol η insertion-stage complexes. (a) Active site conformation in the complex with dATP opposite O^4^MedT viewed into the DNA major groove, and (b), rotated by 90° and viewed perpendicular to the adenine plane. (c) Active site conformation in the complex with dGMPNPP opposite O^4^MedT, viewed into the DNA major groove, and (d), rotated by 90° and viewed perpendicular to the guanine plane. Carbon atoms of O^4^MedT, the incoming nucleotide triphosphate, and selected hPol η amino acid side chains are colored in maroon, orange and magenta, respectively. Mg^2+^ and Ca^2+^ ions are cyan and pink spheres, respectively, and selected H-bonds are shown as dashed lines.

To visualize the O^4^MedT and O^4^EtdT lesions at the active site of hPol η trapped at the insertion stage, we determined four crystal structures of ternary complexes with the Pol bound to a 12mer template strand with the incorporated lesion and paired to an ; 8mer primer and incoming purine nucleoside triphosphate. For details regarding the crystallization, data collection and structure determination and refinement procedures please see the ESI.† Selected crystal data, data collection and refinement parameters and examples of the quality of the final electron density for all structures are summarized and depicted in the Table S4.† The two complexes with O^4^MedT-containing templates and incoming dATP or dGMPNPP reveal similar orientations of the lesion (Fig. 3, PDB ID codes ; 5DLF and ; 5DLG, respectively). Instead of pairing with the incoming nucleotide, O^4^MedT is lodged near the ceiling of the active site. Thus, its base portion is nestled against Trp-64 (base stacking interaction), Met-63 and Ser-62 (hydrophobic contacts between O^4^Me and both Cα and C(=O) from the two residues) from a loop region in the finger domain and Gly-46 from an adjacent β-strand that together form the roof of the active site (Fig. 3a and c). In addition, O^2^ of O^4^MedT and the amino group of Asn-38 are H-bonded (Fig. 3b and d). This position of the O^4^MedT lesion at the entrance of the active site places it quite far away from the incoming nucleotide triphosphate. The distance between its O^4^ atom and N^6^ of dATP is 9 Å (8.2 Å between O^4^ of O^4^MedT and O^6^ of dGMPNPP).

Closer inspection of the positions of the incoming nucleotides shows that adenine stacks on the adjacent t(emplate)A:p(rimer)T pair, with the side chain of Arg-61 from the finger domain hovering closely above the adenine plane and engaged in H-bonds to the α- and β-phosphates of dATP *via* its guanidino moiety (Fig. 3b). By comparison, guanine is shifted into the minor groove and the stacking interaction with the adjacent tA:pT pair is slightly less favorable. The shift is likely a consequence of the altered orientation of the Arg-61 side chain that is extended in the structure of the complex with dGMPNPP, resulting in formation of H-bonds between its guanidino moiety and guanine O^6^ and N7 (Fig. 3d). The particular orientation of the incoming dG brings it closer to Asn-38 from the finger domain, but the distance of 3.64 Å between N2 of the former and the Oε1 oxygen of asparagine is slightly too long for formation of a H-bond.

**Fig. 4:**
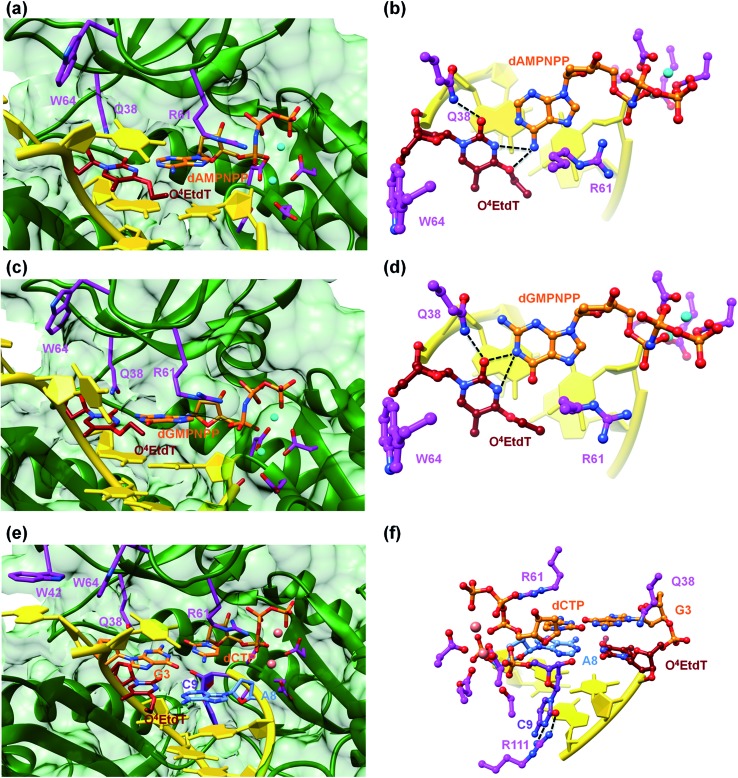
Pairing between incoming purine nucleotide triphosphate and O^4^EtdT in two hPol η insertion-stage complexes (a–d). (a) Major groove view of the Watson–Crick pair between dAMPNPP and O^4^EtdT with formation of bifurcated H-bonds. (b) The active site rotated by 90° relative to A, with the base pair viewed perpendicular to the adenine plane. (c) Major groove view of the sheared configuration between dGMPNPP and O^4^EtdT, with formation of bifurcated H-bonds. (d) The active site rotated by 90° relative to C, with the base pair viewed perpendicular to the guanine plane. Carbon atoms of O^4^EtdT, the incoming nucleotide triphosphate, and selected hPol η amino acid side chains are colored in maroon, orange and magenta, respectively. Mg^2+^ ions are cyan spheres and selected H-bonds are shown as dashed lines. Active site configuration in a ternary hPol η extension-step complex with O^4^EtdT opposite primer dA, followed by template dG opposite incoming dCTP (e and f). (e) Major groove view of the active site, with carbon atoms of O^4^EtdT and the paired dA colored in maroon and light blue, respectively. Carbon atoms of the nascent dG:dCTP pair are colored in orange, carbon atoms of selected hPol η amino acid side chains are colored in magenta, and those of the 3′-terminal primer dC are colored in purple. (f) The active site rotated by 180° relative to A and viewed into the minor groove.

A surface rendering of the hPol η active site in the O^4^MedT insertion-stage complex with the incoming dATP indicates that the nucleobase moiety of the lesion fits snugly into the gap between Trp-64 and Ser-62 (Fig. S49†). It is clear that O^4^EtdT (with the ethyl group in the *syn* conformation) cannot be accommodated in the same fashion, as the longer substituent would clash with residues from the finger domain. Instead, the O^4^EtdT lesion has been pulled inside the active site and pairs with incoming dAMPNPP or dGMPNPP *via* bifurcated H-bonds in the two crystal structures of insertion-stage complexes with this lesion (Fig. 4a–d, PDB ID codes ; 5DQG and ; 5DQH, respectively). As in the complexes with O^4^MedT, Asn-38 forms a H-bond to O^2^ of O^4^EtdT in the minor groove. However, unlike in the O^4^MedT complex with incoming dGMPNPP, the side chain of Arg-61 in the corresponding complex with O^4^EtdT does not adopt an extended conformation to contact the major groove edge of guanine. As can be seen in Fig. 4, Arg-61 is directed toward the triphosphate moiety and forms a salt bridge with the α-phosphate group in both insertion-stage complexes. The methylene group (C1) of the O4 substituent in O^4^EtdT adopts an *anti* conformation in the complex with dAMPNPP (torsion angle C1–O4–C4–N3 = –142°) and a *syn* conformation in the complex with dGMPNPP (torsion angle C1–O4–C4–N3 = +62°). This is a clear difference to the structures of complexes with O^4^MedT, both of which show the lesion adopting a *syn* conformation (torsion angles C1–O4–C4–N3 of +33° and +25° in the dATP and dGMPNPP complexes, respectively). A further difference between the O^4^MedT and O^4^EtdT complexes is constituted by the orientations of template nucleotides 5′-adjacent to the lesions. In the former, A2 and T3 form a stack with Trp-42 outside the active site (Fig. 3). In the O^4^EtdT complexes, A2 is located outside the active site and forms a stacking interaction with Trp-42. However, T3 sits inside the active site and stacks onto O^4^EtdT (dAMPNPP complex; Fig. 4a). In the complex with dGMPNPP, T3 juts into the major groove (Fig. 4c). Thus, neither orientation adopted by T3 in these complexes resembles that of O^4^MedT, lodged near the ceiling of the active site and stacked onto Trp-64.

### Crystal structure of a ternary hPol η·DNA·dCTP extension-stage complex with O^4^EtdT paired opposite primer dA

The structure of a complex with O^4^EtdT paired to dA at the –1 position followed by template dG opposite incoming dCTP was determined at 2.05 Å resolution (Fig. 4e and f, PDB ID codes ; 5DQI). The geometry of the O^4^EtdT:dA pair replicates that seen in the insertion complex with O^4^EtdT opposite incoming dAMPNPP (Fig. 4a, b, e and f). As in the case of the latter, the ethyl group has moved outside the thymine plane and adopts an *anti* orientation (torsion angle C1–O4–C4–N3 = –135°). The base pair itself adopts a Watson–Crick like geometry with formation of a single H-bond; the adjacent dG:dCTP pair displays a standard geometry with three H-bonds. Arg-61 is directed toward the phosphate moieties of the incoming nucleotide and forms two salt bridges with the α- and β-phosphate groups, and Asn-38 is engaged in two H-bonds with N3 and O4′ of template dG.

The most unusual feature of the extension-stage structure is the presence of an additional nucleotide at the 3′-end of the primer (Fig. 4f). Because the crystallization solutions contained dCTP and the residual electron density is consistent with a pyrimidine, we extended the primer by dC (Fig. S50†). We suspect that hPol η possesses weak catalytic activity with Ca^2+^ as the cofactor or that traces of Mg^2+^ present in the crystallizations led to primer extension *in situ* (even a very low activity could result in extension by a single nucleotide over the course of two weeks). We showed earlier that the translesion DNA polymerase Dpo4 from *Sulfolobus solfataricus* is able to catalyze nucleotide insertion with Ca^2+^, although the activity is far below that seen with Mg^2+^ as the prosthetic group.^21^ The additional dC stacks against the backbone of the template strand in the minor groove and its position is further stabilized by two H-bonds between N3 and O2 and the guanidino moiety of Arg-111 (Fig. 4f).

## Discussion

The known toxicity of alkylated adducts at the O^4^-position of thymidine prompted us to explore the influence of restricting orientation of the alkyl group around the C4–O^4^ bond to an *anti* conformation in translesion synthesis catalyzed by hPol η. In these studies, the bicyclic pyrimidine analogs DFP and TTP, which link the C5 and O^4^ atoms with a di- or trimethylene linker, were evaluated in addition to O^4^MedT and the bulkier O^4^EtdT lesion. Conformationally locked analogs of damage that can occur at the nucleobase have been previously synthesized and employed in studies which have provided insights into the requirements for DNA repair processes.^22^,^23^

UV thermal denaturation studies of oligomers containing DFP and TTP revealed similar influences on duplex stability to both complementary and mismatched nucleobases compared to O^4^MedT and O^4^EtdT. The most stable pairing of either DFP or TTP was with dG, also observed with O^4^MedT and O^4^EtdT. NMR studies of a duplex containing an O^4^MedT·dG pair revealed, in addition to the O^4^-methyl group adopting a *syn*-conformation, that the base formed a Watson–Crick “like” pairing with a single hydrogen bond.^24^ In this structure, the *syn*-orientation of the O^4^–Me group influences the hydrogen bond between the imino proton of dG and the N3 atom of O^4^MedT by increasing the distance between the O^6^ and O^4^ atoms of dG and O^4^MedT, respectively. Limiting the orientation of the methylene group at the O^4^-atom to the *anti*-conformation, in the case of the DFP and TTP modifications, appears to have a minimal impact on the interaction with dG and duplex stability. In pairing with dA, a similar drop in duplex stability compared with dT is observed for oligonucleotides containing the O^4^MedT, O^4^EtdT, DFP, and TTP modifications. The NMR structure of a duplex containing an O^4^MedT·dA pair indicated that the O^4^–Me group is *syn* and that the bases adopt a wobble alignment with one hydrogen bond formed between the imino nitrogen of O^4^MedT and the amino group of dA.^15^ The restricted *anti*-orientation of the methylene group for DFP or TPP modifications does not significantly impact duplex stability compared to O^4^MedT or O^4^EtdT.

Steady-state kinetics of individual nucleotide incorporation opposite the DFP and TTP modifications by hPol η demonstrated preferred insertion of purine nucleotides relative to the pyrimidines, similar to O^4^MedT and O^4^EtdT. The efficiency of nucleotide insertion (*k*_cat_/*K*_m_) for the correct nucleotide (dAMP) across the lesions followed the order DFP > TPP > O^4^MedT > O^4^EtdT. For dGMP, a similar efficiency of nucleotide insertion occurred for DFP, TPP, and O^4^MedT whereas a drop was observed for O^4^EtdT. In agreement with studies involving *Saccharomyces cerevisiae* DNA polymerase η (yPol η), a reduction in incorporation efficiency due to the presence of an O^4^MedT insert was observed.^17^ However, whereas the yeast homolog revealed a significant preference for dGMP, which was incorporated approximately 80 times more efficiently than dAMP,^17^ hPol η displayed almost equal selectivity at incorporating dAMP (*f* = 0.94) as dGMP opposite O^4^MedT. A comparable 80-fold preference for dGMP over dAMP was exhibited by yPol η for the bulkier O^4^-carboxymethylthymidine lesion.^25^ The rationale for the preferred incorporation of dGMP opposite O^4^MedT by yPol η was attributed to a dG·O^4^MedT wobble base pairing. Differences observed for nucleotide incorporation opposite O^4^MedT by the yeast and human homologs of Pol η may be influenced in part by different sequence contexts, as previously observed.^5^,^26^ In addition, homologs of Pol η have exhibited differences in nucleotide incorporation across some types of DNA damage. For example, yPol η accurately inserts dCMP across 8-oxodG whereas hPol η is less accurate, inserting some dAMP across this lesion as well.^5^,^27^ Interestingly, similar misinsertion profiles have been observed in bypass experiments of hPol η and yPol η with O^6^MedG, a lesion which also protrudes in the major groove of the DNA duplex.^28^

For the bulkier O^4^EtdT and conformationally restricted analogs DFP and TTP, a preference for nucleotide incorporation of dAMP over dGMP was observed. For O^4^EtdT, hPol η was more proficient at incorporating dAMP over dGMP with catalytic efficiencies of 0.10 and 0.06 μM^–1^ s^–1^, respectively. These values are approximately two-fold lower compared to those observed for the O^4^MedT-containing template, but can be rationalized by the increased bulk of the ethyl group, which may influence dNTP incorporation in the hPol η active site. Ethylation of the O^4^-position of dT has been shown to stall the human Y-family DNA polymerases hPol κ and hPol ι but not hPol η (although steady-state analysis was not reported for oligonucleotides containing O^4^EtdT).^18^ Bypass of O^4^EtdT by hPol η revealed dGMP misincorporation at 55% compared to 19% for dAMP insertion, in agreement with our data despite different sequence contexts.

For the conformationally restricted DFP and TPP modifications, incorporation efficiency was observed to be ∼1.5-fold higher for dAMP (0.27 and 0.24 μM^–1^ s^–1^, respectively) and comparable for dGMP relative to O^4^MedT. These results demonstrate that hPol η is more proficient at incorporating both the correct (dAMP) and incorrect (dGMP) nucleotides across from these more conformationally restricted lesions. In addition, the increase in steric bulk from the DFP to TPP slightly decreases incorporation efficiency. Exposure of the hydrogen bonding face of the DFP or TPP modifications may have a greater influence on stabilizing the wobble alignment geometry that has been suggested for the O^4^MedT·dA pairing. The conformational restriction of the alkyl group to an *anti*-orientation around the C4–O^4^ bond, as in the DFP and TPP modifications, would direct the O^4^-methylene group away from the amino group of dA, which could account for the enhancement of incorporation of the correct nucleotide (dAMP) compared to O^4^MedT. Incorporation of dGMP may not be as influenced by orientation of the alkyl group around the C4–O^4^ bond as the proposed hydrogen bonding interaction, based on the NMR structure of the duplex containing the O^4^MedT·dG, which occurs between the amino group of dG and O^2^-atom of O^4^MedT.^24^ In the case of O^4^EtdT, the combination of the *syn*-orientation and the size of the ethyl group may both contribute to the reduced efficiency of nucleotide insertion of dAMP and dGMP in this series.

Primer extension reactions in the presence of all four dNTPs for templates containing the O^4^MedT, O^4^EtdT, DFP, and TTP modifications demonstrated that hPol η was proficient at incorporating nucleotides across and past the adducted site. However, both O^4^MedT and O^4^EtdT exhibited a greater accumulation of non-full length oligonucleotide products at reduced reaction times (30 and 60 min), which was not observed for bicyclic DFP or TPP analogs. The LC-MS/MS analyses of the extension products from the *in vitro* primer bypass studies revealed that dGMP incorporation across the lesion was preferred over dAMP in all cases except the control (dT). The ratio of dGMP : dAMP incorporation by hPol η, assessed from the extension products, was found to decrease in the series TPP (4.6 : 1) > DFP (3.3 : 1) ≈ O^4^EtdT (3.2 : 1) > O^4^MedT (2.1 : 1). The presence of the larger alkyl group for O^4^EtdT or the analogs with the O^4^-methylene group in an *anti*-conformation (TPP and DFP) clearly promotes dGMP misincorporation in the presence of all four nucleotides. In addition, the DFP and TPP modification were not found to induce a significant amount of frameshifts in the products compared to O^4^MedT. Differences in fidelity observed between the steady-state kinetic and LC-MS/MS full-length experiments have been observed previously.^17^,^19^ The variance may be attributed to accommodation of the incoming dGTP relative to dATP for these modifications, highlighting that adduct size and the conformation of the O^4^-methylene group can influence interactions in the active site of hPol η. It should be noted, however, that other steric and/or stereoelectronic effects may have an impact on hPol η bypass processivity of the conformationally locked analogues relative to O^4^MedT or O^4^EtdT, respectively. In the case of the analogs investigated, hPol η continued extension of the primer in an error-free manner after incorporation of dATP or dGTP across from the damaged site on the template.

Several observations based on nucleotide incorporation profiles attest to the distinct effects on hPol η bypass synthesis exerted by the O^4^MedT and O^4^EtdT lesions. These concern (i) the more error-prone bypass caused by O^4^MedT, *i.e.* dGTP is favored relative to dATP (Fig. 2a), (ii) increased accumulation of the +1 product in the full-length extension reaction for O^4^MedT (Fig. 1), and (iii) significantly more frameshift products caused by the O^4^MedT lesion (Fig. 2b). Interestingly, the structural data for insertion-stage hPol η complexes with either O^4^MedT or O^4^EtdT in the template strand reveal starkly different orientations of the two adducted nucleotides at the active site. O^4^MedT is trapped in an orientation that keeps it at a considerable distance from the incoming purine nucleotide triphosphates. Conversely, O^4^EtdT pairs opposite both dATP and dGTP with formation of bifurcated H-bonds (whereby the latter pair features a sheared orientation of the two partners, with G being pushed toward the minor groove). The increased proclivity for insertion of dG opposite O^4^MedT compared to O^4^EtdT is not surprising if one considers the strict preference by the O^4^-methyl substituent for a *syn* conformation. The *syn* conformation precludes adoption of an O^4^MedT:dA pair with standard Watson–Crick geometry, but the sheared pairing mode seen in the case of O^4^EtdT:dG(MPNPP) (Fig. 4c and d), also presumably adopted by the O^4^MedT:dG pair, is compatible with a *syn* conformation of the substituent. This conclusion is borne out by the observations that the ethyl moiety in the O^4^EtdT:dA(MPNPP) pairs assumes an *anti* conformation (Fig. 4a, b, e and f), whereas its conformation is *syn* in the O^4^EtdT:dGMPNPP pair. Furthermore, the TPP adduct opposite dGMPNPP was modeled from the O^4^EtdT:dG(MPNPP) ternary crystal structure coordinates. The configuration of the adduct seen in the model is consistent with the enhanced incorporation of dG observed in the primer extension experiments since the constrained *anti* conformation of the bicyclic system does not hinder the guanine nucleobase from shifting towards the major groove and potentially form two H-bonds with TPP (Fig. S51†).

On one hand, one could argue that the higher fraction of frameshifts for O^4^MedT relative to O^4^EtdT is consistent with the structural data that show the former is not engaged opposite the incoming nucleotide but trapped adjacent to the ‘entrance’ of the active site. Perhaps the more pronounced accumulation of the +1 product in the case of the full-length extension reactions opposite O^4^MedT compared to the other O^4^ adducts tested here are the result of non-templated insertion. Thus, purine nucleoside triphosphates would be favored and their incorporation would not be affected by the particular conformation of the O^4^-methyl group, *syn* or *anti*. This scenario is certainly not inconsistent with the structural data that reveal no interaction between the O^4^MedT lesion and the incoming dATP or dGMPNPP. Clearly, it is intriguing that both activity and structural data show distinct consequences of the O^4^MedT and O^4^EtdT lesions for bypass by hPol η. However, it is important to note that the position of the O^4^MedT lesion at the active site, unique among all crystal structures of hPol η complexes analyzed to date, represents one state during bypass. Perhaps other orientations and interactions of the adducted nucleotide occur during bypass, which precludes all steps involved in the mechanism of O^4^-alkyl bypass synthesis by hPol η.

All results from the study (kinetic evaluation, full extension assays and crystal structures) may be integrated into one potential extension model. The crystal structure supports the notion that the O^4^MedT nucleobase is indeed nestled at the ceiling for an undefined period of time. The purine nucleoside triphosphates could then be imported into the active site, and would be subject to a template gap, analogous to being opposite an abasic site. According to full extension assays, there are approximately twice as many dG inserted by hPol η relative to dA. The lack of O^4^MedT – incoming dNTP clash in the active site may explain higher *k*_p_ values observed for dATP and dGTP for O^4^MedT compared to O^4^EtdT (by 8.8 or 9.3 fold respectively). This may also aid in explaining the preference for purine insertion, in an approximate 2 : 1 dG : dA ratio across O^4^MedT, whereas a larger ratio is observed for all the other modifications as discussed above. Perhaps the other modifications prevent the modified nucleobases from accessing the conformation at the top of the active site. The ethyl group, albeit bulkier, can populate the other conformations (*syn versus anti*) as observed in the crystallographic data, which may contribute to the higher correct dA insertion relative to the bicyclic analogues which are locked in an *anti* conformation. Increase in bulk (from O^4^MedT to O^4^EtdT and DFP to TPP) leads to an increase of incorrect dG insertion. Eventually the O^4^MedT is required to move from the top of the active site back to the 0 or –1 (post-replicative) position(s). It is possible that this mobility causes the frameshift adduct formation observed in the full extension assays. As a result, we have provided intriguing insights on a potentially different bypass mechanism of O^4^MedT in comparison to the larger O^4^EtdT adduct.

## Conclusions

Oligonucleotides containing DFP and TPP, designed as analogs of O^4^-alkylated thymidine, were synthesized to explore the influence of limiting the O^4^-alkyl lesion to an *anti*-orientation on nucleotide incorporation by hPol η. These modifications were shown to destabilize the DNA duplex, based on UV thermal denaturation studies, regardless of the base-pairing partner (A, G, T, or C), similar to O^4^MedT and O^4^EtdT. Primer extension assays demonstrated that these pyrimidyl modifications hindered nucleotide incorporation by hPol η. Single nucleotide incorporation studies revealed increased selectivity towards dAMP over dGMP that followed the order O^4^EtdT > DFP ≈ TPP. A slight preference for dGMP over dAMP incorporation was observed for O^4^MedT. LC-MS/MS analysis of primer extension studies (in the presence of all four dNTPs) revealed that hPol η incorporated dGMP over dAMP across the lesions in the order TPP > DFP ≈ O^4^EtdT > O^4^MedT. These trends suggest that limiting the orientation of the O^4^-alkylene group enhances the proficiency of dNTP incorporation by hPol η across O^4^-alkylated dT damage. In the presence of all four dNTPs, error-prone nucleotide incorporation by hPol η is enhanced by restricting the O^4^-lesion to an *anti*-orientation. This study exemplifies how restricting a lesion's conformational freedom impacts bypass profile by hPol η. Moreover, our results provide mechanistic insights into the mutagenicity of the biologically relevant O^4^MedT and O^4^EtdT DNA adducts.

## Supplementary Material

Supplementary information
