# Supplementary material for: Lesion orientation of O4-alkylthymidine influences replication by human DNA polymerase η
Source: Chem Sci. 2016 Apr 26;7(8):4896–904. doi: 10.1039/c6sc00666c (PMC5001687; doi:10.1039/c6sc00666c)
Supplement: Supplementary file 1 [file SC-007-C6SC00666C-s001.pdf]

Electronic Supplementary Material (ESI) for Chemical Science.
This journal is © The Royal Society of Chemistry 2016

Supplementary Information

Lesion Orientation of O^4-Alkylthymidine Influences Replication by Human DNA Polymerase η

Derek K. O'Flaherty[1], Amritraj Patra[2], Yan Su[2], F. Peter Guengerich[2], Martin Egli[2,*] and Christopher J. Wilds[1,*]

[1]Department of Chemistry & Biochemistry, Concordia University, Montréal, Québec H4B1R6, Canada
[2]Department of Biochemistry, Vanderbilt Institute of Chemical Biology, Vanderbilt Ingram Cancer Center, Center in Molecular Toxicology, and Center for Structural Biology, School of Medicine, Vanderbilt University, Nashville, Tennessee 37232, United States

Contents **Page**

Supplementary Methods

General methods for the preparation and characterization of nucleosides **S-4**
Chemical synthesis of nucleosides **S-4**
Solid phase synthesis and purification of oligonucleotides **S-10**
Oligonucleotide characterization by ESI-MS and nuclease digestion **S-11**
UV thermal denaturation studies of DNA duplexes **S-11**
Circular dichroism (CD) spectroscopy of DNA duplexes **S-11**
Molecular modeling of DNA duplexes **S-12**
Steady-state kinetics with hPol η **S-12**
LC-MS/MS analysis of full-length extension products **S-12**
Pre-steady-state kinetics with hPol η **S-13**
Crystallization **S-13**
X-ray Diffraction Data Collection, Structure Determination and Refinement **S-14**

Supplementary Notes

Synthesis and characterization of nucleosides **S-15**
Synthesis of DNA containing single modification inserts **S-15**
UV thermal denaturation of DNA duplexes and mismatch studies **S-16**
Circular dichroism spectroscopy and molecular modeling of DNA duplexes **S-17**

Supplementary Schemes, Figures and Tables

Supplementary Scheme S1 - Synthesis of compounds **6a** and **6b** **S-18**
Supplementary Figure S1 - 500 MHz ^{1}H NMR spectrum of compound (**2a**) (in d_6-DMSO) **S-19**
Supplementary Figure S2 - 125.7 MHz ^{13}C NMR spectrum of compound (**2a**) (in d_6- **S-20**

	DMSO)	
**Supplementary Figure S3 -**	HR ESI-MS spectrum of compound (**2a**)	**S-21**
**Supplementary Figure S4 -**	500 MHz ^{1}H NMR spectrum of compound (**2b**) (in d_6-DMSO)	**S-22**
**Supplementary Figure S5 -**	125.7 MHz ^{13}C NMR spectrum of compound (**2b**) (in d_6-DMSO)	**S-23**
**Supplementary Figure S6 -**	HR ESI-MS spectrum of compound (**2b**)	**S-24**
**Supplementary Figure S7 -**	500 MHz ^{1}H NMR spectrum of compound (**3a**) (in d_6-DMSO)	**S-25**
**Supplementary Figure S8 -**	125.7 MHz ^{13}C NMR spectrum of compound (**3a**) (in $CDCl_3$)	**S-26**
**Supplementary Figure S9 -**	HR ESI-MS spectrum of compound (**3a**)	**S-27**
**Supplementary Figure S10 -**	500 MHz ^{1}H NMR spectrum of compound (**3b**) (in $CDCl_3$)	**S-28**
**Supplementary Figure S11 -**	125.7 MHz ^{13}C NMR spectrum of compound (**3b**) (in $CDCl_3$)	**S-29**
**Supplementary Figure S12 -**	HR ESI-MS spectrum of compound (**3b**)	**S-30**
**Supplementary Figure S13 -**	500 MHz ^{1}H NMR spectrum of compound (**4a**) (in $CDCl_3$)	**S-31**
**Supplementary Figure S14 -**	125.7 MHz ^{13}C NMR spectrum of compound (**4a**) (in $CDCl_3$)	**S-32**
**Supplementary Figure S15 -**	HR ESI-MS spectrum of compound (**4a**)	**S-33**
**Supplementary Figure S16 -**	500 MHz ^{1}H NMR spectrum of compound (**4b**) (in $CDCl_3$)	**S-34**
**Supplementary Figure S17 -**	125.7 MHz ^{13}C NMR spectrum of compound (**4b**) (in $CDCl_3$)	**S-35**
**Supplementary Figure S18 -**	HR ESI-MS spectrum of compound (**4b**)	**S-36**
**Supplementary Figure S19 -**	500 MHz ^{1}H NMR spectrum of compound (**5a**) (in $CDCl_3$)	**S-37**
**Supplementary Figure S20 -**	125.7 MHz ^{13}C NMR spectrum of compound (**5a**) (in $CDCl_3$)	**S-38**
**Supplementary Figure S21 -**	HR ESI-MS spectrum of compound (**5a**)	**S-39**
**Supplementary Figure S22 -**	500 MHz ^{1}H NMR spectrum of compound (**5b**) (in $CDCl_3$)	**S-40**
**Supplementary Figure S23 -**	125.7 MHz ^{13}C NMR spectrum of compound (**5b**) (in d_6-DMSO)	**S-41**
**Supplementary Figure S24 -**	HR ESI-MS spectrum of compound (**5b**)	**S-42**
**Supplementary Figure S25 -**	500 MHz ^{1}H NMR spectrum of compound (**6a**) (in d_6-acetone)	**S-43**
**Supplementary Figure S26 -**	125.7 MHz ^{13}C NMR spectrum of compound (**6a**) (in d_6-acetone)	**S-44**
**Supplementary Figure S27 -**	202.3 MHz ^{31}P NMR spectrum of compound (**6a**) (in d_6-acetone)	**S-45**
**Supplementary Figure S28 -**	HR ESI-MS spectrum of compound (**6a**)	**S-46**
**Supplementary Figure S29 -**	500 MHz ^{1}H NMR spectrum of compound (**6b**) (in d_6-acetone)	**S-47**
**Supplementary Figure S30 -**	125.7 MHz ^{13}C NMR spectrum of compound (**6b**) (in d_6-acetone)	**S-48**
**Supplementary Figure S31 -**	202.3 MHz ^{31}P NMR spectrum of compound (**6b**) (in d_6-acetone)	**S-49**
**Supplementary Figure S32 -**	HR ESI-MS spectrum of compound (**6b**)	**S-50**
**Supplementary Figure S33 -**	SAX-HPLC profile of purified $\mathbf{S_{DFP}}$ and $\mathbf{S_{TPP}}$	**S-51**
**Supplementary Figure S34 -**	ESI MS of oligonucleotide $\mathbf{S_{DFP}}$	**S-52**
**Supplementary Figure S35 -**	ESI MS of oligonucleotide $\mathbf{S_{TPP}}$	**S-53**
**Supplementary Figure S36 -**	C-18 HPLC profile of digested oligomer $\mathbf{S_{DFP}}$ at (a) 260 nm and (b) 300 nm.	**S-54**
**Supplementary Figure S37 -**	C-18 HPLC profile of digested oligomer $\mathbf{S_{TPP}}$ at (a) 260 nm and (b) 300 nm	**S-55**
**Supplementary Figure S38 -**	T_m values (^{o}C) of duplexes containing dT, O^4MedT, O^4EtdT,	**S-56**

	DFP or TPP across different base pairing partners	
**Supplementary Figure S39 -**	Far-UV circular dichroism spectra of DNA sequences	**S-57**
**Supplementary Figure S40 -**	Geometry optimized models of DNA duplexes	**S-58**
**Supplementary Figure S41 -**	Top view of geometry optimized models of the DNA duplexes	**S-59**
**Supplementary Figure S42**	Steady-state incorporation efficiencies opposite dT, O^4MedT, O^4EtdT, DFP, and TPP by hPol η with individual dNTPs.	**S-60**
**Supplementary Figure S43 -**	LC-MS analysis of the most abundant full-length extension products opposite unmodified dT in the DNA template by hPol η in the presence of all four dNTPs	**S-61**
**Supplementary Figure S44 -**	LC-MS analysis of the most abundant full-length extension products opposite O^4MedT in the DNA template by hPol η in the presence of all four dNTPs	**S-62**
**Supplementary Figure S45 -**	LC-MS analysis of the most abundant full-length extension products opposite O^4EtdT in the DNA template by hPol η in the presence of all four dNTPs	**S-63**
**Supplementary Figure S46 -**	LC-MS analysis of the most abundant full-length extension products opposite DFP in the DNA template by hPol η in the presence of all four dNTPs.	**S-64**
**Supplementary Figure S47 -**	LC-MS analysis of the most abundant full-length extension products opposite TPP in the DNA template by hPol η in the presence of all four dNTPs.	**S-65**
**Supplementary Figure S48 -**	Pre-steady-state kinetic plots of incorporation of dATP, dGTP by hPol η opposite O^4MedT, O^4EtdT, DFP, and TPP, and unmodified control (dT)	**S-66**
**Supplementary Figure S49 -**	Space filling model of the hPol *h* active site with O^4MedT wedged between Trp-64 and Ser-62.	**S-68**
**Supplementary Figure S50 -**	Quality of the final models of ternary hPol η complexes with O^4MedT/O^4EtdT-adducted DNA template strands.	**S-69**
**Supplementary Figure S51 -**	Model of the active site configuration in the ternary hPol η insertion-step complex with dGMPNPP opposite TPP.	**S-71**
**Supplementary Table S1 -**	Steady-state kinetics of incorporation of dATP, dGTP, dCTP and dTTP opposite dT, O^4MedT, O^4EtdT, DFP, and TPP by hPol η.	**S-72**
**Supplementary Table S2 -**	LC-MS/MS analysis of full-length extension products opposite dT, O^4MedT, O^4EtdT, DFP and TPP by hPol η (values are reported in %)	**S-73**
**Supplementary Table S3 -**	Pre-steady-state kinetic parameters of incorporation of dATP, dGTP by hPol η opposite O^4MedT, O^4EtdT, DFP, and TPP, and unmodified control (dT)	**S-73**
**Supplementary Table S4 -**	Crystal data, data collection parameters and structure refinement statistics	**S-74**
**References**		**S-76**

Supplementary Methods

General methods for the preparation and characterization of nucleosides.

1-(*α*)-Chloro-3,5-di-*O*-(p-toluoyl)-2-deoxy-D-ribose was purchased from Berry & Associates, Inc. (Dexter, MI). *N*,*N*-Diisopropylaminocyanoethylphosphonamidic chloride was purchased from ChemGenes Corporation (Wilmington, MA). 5-*O*-Dimethoxytrityl-2'-deoxyribonucleoside-3-*O*-(*β*-cyanoethyl-*N*,*N*-diisopropyl)phosphoramidites and protected 2´-deoxyribonucleoside-CPG supports were purchased from Glen Research (Sterling, Virginia). All other chemicals and solvents were purchased from the Aldrich Chemical Company (Milwaukee, WI) or EMD Chemicals (Gibbstown, NJ). Flash column chromatography was performed using silica gel 60 (230-400 mesh) obtained from Silicycle (Quebec City, QC). Thin layer chromatography (TLC) was carried out using precoated TLC plates (Merck, Kieselgel 60 F_{254}, 0.25 mm) purchased from EMD Chemicals Inc. (Gibbstown, NJ). NMR spectra were recorded on a Varian 500 MHz NMR spectrometer at room temperature. ^{1}H NMR spectra were recorded at a frequency of 500.0 MHz and chemical shifts were reported in parts per million (ppm) downfield from tetramethylsilane. ^{13}C NMR spectra (^{1}H decoupled) were recorded at a frequency of 125.7 MHz and chemical shifts were reported in ppm with tetramethylsilane as a reference. ^{31}P NMR spectra (^{1}H decoupled) were recorded at a frequency of 202.3 MHz and chemical shifts were reported in ppm with H_3PO_4 used as an external standard. High resolution mass spectrometry of modified nucleosides were carried out using an LTQ Orbitrap Velos - ETD mass spectrometer (Thermo Scientific) at the Concordia University Centre for Biological Applications of Mass Spectrometry (CBAMS) or using a 7T-LTQ FT ICR mass spectrometer (Thermo Scientific) at the Concordia University Centre for Structural and Functional Genomics. The mass spectrometer was operated in full scan, positive ion detection mode. ESI mass spectra for oligonucleotides were obtained at CBAMS using a Micromass Qtof2 mass spectrometer (Waters) equipped with a nanospray ion source. The mass spectrometer was operated in full scan, negative ion detection mode.

Chemical synthesis of nucleosides.

Compounds **1a** and **1b** were prepared according to published procedures. [1,2,3,4,5]

5-Hydroxyethyluracil (**2a**)

To a solution of NaOEt (1.23 g, 18.1 mmol) in EtOH (90 mL) was added compound **1a** (7.55 g, 48.4 mmol). After 16h, the solvent was removed in vacuo and the crude was taken up in a minimal amount of boiling water. The solution was acidified (to a pH ~ 2-3) on an ice bath and the precipitate was isolated by vacuum filtration and washed with cold EtOH (3 × 40 mL). To maximize the yield, the filtrate was evaporated and the solid was resuspended in EtOH (50 mL). The solid was isolated and washed once more according to the procedure described above to give **2a** in an overall yield of 7.16 g (94.8%) as a colorless powder. $\lambda_{max(MeCN)}$ 262 nm. ^{1}H NMR (500MHz, d_6-DMSO, ppm): δ 10.99 (broad s, 1H, NH), 10.64 (broad s, 1H, NH), 7.19 (s, 1H, H6), 4.52 (t, 1H, OH, J = 5.5 Hz), 3.44 (m, 2H, $\underline{CH_2}$OH), 2.30 (t, 2H, CH_2Ar, J = 6.5 Hz). ^{13}C NMR (125.7 MHz, d_6-DMSO, ppm): 164.6, 151.4, 138.8, 109.1, 59.44, 29.7. IR (thin film); ν_{max}

(cm^{-1}) 3680, 2922, 2849, 2382, 2349, 1755, 1674, 1451, 1244, 1032. HRMS (ESI-MS) *m/z* calculated for $C_6H_8N_2O_3Na^+$ 179.0427: found 179.0435 $[M+Na]^+$.

5-Hydroxypropyluracil (**2b**)

To a solution of NaOEt (1.52 g, 22.4 mmol) in EtOH (225 mL) was added compound **1b** (4.76 g, 28.0 mmol). After 16h, the solvent was removed in vacuo and the crude was taken up in a minimal amount of boiling water. The solution was acidified (to a pH ~ 2-3) and cooled, and the precipitate was isolated by vacuum filtration and washed with cold EtOH (3 × 50 mL). To maximize the yield, the filtrate was evaporated and the solid was resuspended in EtOH (50 mL). The solid was isolated and washed once more according to the procedure described above to give **2b** in an overall yield of 4.75 g (>99%) as a colorless powder. $\lambda_{max(MeCN)}$ 261 nm. ^1H NMR (500 MHz, d_6-DMSO, ppm): δ 10.92 (broad s, 1H, NH), 10.54 (broad s, 1H, NH), 7.14 (s, 1H, H6), 4.63 (t, 1H, OH, J = 5.0 Hz), 3.35 (m, 2H, $\underline{CH_2}OH$), 2.17 (t, 2H, CH_2Ar, J = 8 Hz), 1.54 (m, 2H, $CH_2\underline{CH_2}CH_2$). ^{13}C NMR (125.7 MHz, d_6-DMSO, ppm): 164.9, 151.7, 138.1, 111.9, 61.7, 31.3, 23.1. IR (thin film); ν_{max} (cm^{-1}) 3680, 2922, 2849, 2381, 2349, 1755, 1674, 1451, 1244, 1032. HRMS (ESI-MS) *m/z* calculated for $C_7H_{10}N_2O_3Na^+$ 193.0584: found 193.0594 $[M+Na]^+$.

3´5´-*O*-*bis*-Toluoyl-5-hydroxyethyl-2´-deoxyuridine (**3a**)

To a solution of compound **2a** (2.4 g, 15.4 mmol) in HMDS (60 mL, 164 mmol) was added TMS-Cl (0.4 mL, 3.14 mmol) which was then stirred vigorously at 140 ^oC. After 5 h the excess HMDS was removed in vacuo and the resulting gum was left on the high vacuum for 2 h. The gum was then dissolved in 1,2-dichloroethane (60 mL) and to this was added a solution of 1-(α)-chloro-3,5-di-*O*-(*p*-toluoyl)-2-deoxy-D-ribose (2.0 g, 5.1 mmol) in 1,2-dichloroethane (80 mL) while stirring at room temperature. After 16 h the solvent was removed in vacuo and the residue was taken up in CH_2Cl_2 (60 mL) then washed with 3% (aq) $NaHCO_3$ (w/v, 2 × 50 mL). The organic layer was dried over anhydrous Na_2SO_4, decanted and the solvent evaporated. Then, 1 M TBAF (in THF, 5 mL, 5 mmol) was added and the reaction stirred for 15 min. The solvent was removed and the mixture of anomers was purified *via* flash column chromatography using a $CH_3OH:CH_2Cl_2$ solvent system (2% → 5%, v/v). The β-anomer was isolated by precipitation from EtOAc: hexanes (1:1, v/v) at a concentration of 31 mM with stirring at room temperature for 10 min. The resulting suspension was filtered under vacuo to afford 1.15 g (44%) of **3a** as a colorless foam. R_f (SiO_2 TLC): 0.33 $CH_3OH:CH_2Cl_2$ (5%, v/v). $\lambda_{max(MeCN)}$ 241 nm. ^1H NMR (500MHz, d_6-DMSO, ppm): δ 11.37 (s, 1H, NH), 7.92-7.87(m, 4H, Ar), 7.48 (s, 1H, H6), 7.36-7.30 (m, 4H, Ar), 6.28 (t, 1H, H1’, J = 8 Hz), 4.61 (m, 1H, H3’), 4.55 (m, 1H, H5’), 4.51 (m, 1H, H5’’), 4.46 (m, 1H, H4’), 3.37 (m, 2H, $\underline{CH_2}OH$), 2.95 (m, 1H, H2’), 2.53 (m, 1H, H2’’), 2.39 (s, 3H, CH_3Ph), 2.38 (s, 3H, CH_3Ph), 2.24 (t, 2H, CH_2Ar, J = 7.0 Hz). ^{13}C NMR (125.7 MHz, $CDCl_3$, ppm): 166.6, 166.5, 163.6, 150.8, 145.1, 145.0, 136.8, 130.4, 130.1, 130.0, 129.8, 127.1, 126.8, 112.9, 85.5, 83.2, 75.3, 64.7, 60.8, 38.5, 30.6, 22.3, 22.2. IR (thin film); ν_{max} (cm^{-1}) 3204, 3064, 2955, 2925, 1719, 1685, 1612, 1510, 1464, 1269, 1178, 1100, 1020. HRMS (ESI-MS) *m/z* calculated for $C_{27}H_{29}N_2O_8^+$ 509.1918: found 509.1935 $[M+H]^+$.

Alternative method for resolution of the anomers: To a solution of the purified mixture of anomers (3.7 g, 7.3 mmol) and imidazole (1.6 g, 23 mmol) in CH_2Cl_2 (80 mL) was added TBS-Cl (1.8 g, 12 mmol) under stirring. After 4h the solvent was removed in vacuo and the content

was taken up in DCM (50 mL), washed with 3% (aq.) $NaHCO_3$ (2 × 50 mL), dried over anhydrous Na_2SO_4, decanted and the solvent evaporated. Purification was performed by flash column chromatography using an EtOAc: Hex solvent system (1:4, then 1:3, v/v) to afford 3.1 g (65%) of the β-anomer and 0.82 g (17 %) of the α-anomer, both as colorless foams. To a solution of the *β*-anomer **3a** (2.6 g, 4.1 mmol) in THF (40 mL) was added 1 M TBAF (in THF, 4.9 mL, 4.9 mmol) which was heated to 45°C under stirring. After 1 h the solvent was removed in vacuo and the residue was taken up in DCM (50 mL) then washed with 3% (aq., w/v) $NaHCO_3$ (2 × 50 mL). The organic layer was dried over anhydrous Na_2SO_4, decanted and the solvent was evaporated. Purification was achieved by flash column chromatography using a CH_3OH:CH_2Cl_2 solvent system (1% → 3%, v/v) to afford 2.0 g (96 %) of **3a** as a colorless foam.

3´,5´-*O*-*bis*-Toluoyl-5-hydroxypropyl-2´-deoxyuridine (**3b**)

To a solution of compound **2b** (4.5 g, 26.4 mmol) in HMDS (95 mL) was added TMS-Cl (0.73 mL, 5.74 mmol) while stirred at 135°C. After 5h, the excess HMDS was removed in vacuo and the resulting gum was placed on the high vacuum for 1 h. To a solution of the gum in 1,2-dichloroethane (225 mL) was added 1-(*α*)-chloro-3,5-di-*O*-(*p*-toluoyl)-2-deoxy-D-ribose (3.5 g, 9.0 mmol) and the atmosphere was exchanged with Ar while stirring at room temperature. After 16h the solvent was removed in vacuo and the crude was taken up in CH_2Cl_2 (125 mL), washed with cold brine (50 mL) followed by cold 3% (aq., w/v) $NaHCO_3$ (2 × 50mL). The organic layer was dried over anhydrous Na_2SO_4, decanted and the solvent was evaporated. The product was purified *via* flash column chromatography (short column) using an isocratic CH_3OH:CH_2Cl_2 solvent system (4%, v/v). A mixture of silylated and desilylated products were isolated to which 1 M TBAF (in THF, 5 mL, 5 mmol) was added under stirring. After 15 min, the solvent was removed in vacuo and the mixture of desilylated anomers were purified by flash column chromatography using a CH_2Cl_2:CH_3OH solvent system (2 % → 4 % by increments of 0.5 %, v/v). The *β*-anomer was isolated by precipitation from EtOAc at a concentration of 31 mM with stirring at room temperature for 10 min. The resulting suspension was filtered under vacuo to afford 2.05 g (44 %) of **3b** as a colorless foam. R_f (SiO_2 TLC): 0.41 CH_3OH:CH_2Cl_2 (5%, v/v). $\lambda_{max(MeCN)}$ 242 nm. ^1H NMR (500MHz, $CDCl_3$, ppm): δ 7.92-7.89 (m, 4H, Ar), 7.28 (s, 1H, H6), 7.25-7.23 (m, 4H, Ar), 6.41 (dd, 1H, H1´, *J* = 8.5, 8.75 Hz), 5.61 (m, 1H, H3´), 4.78 (dd, 1H, H5´, *J* = 3.5, 12.5 Hz), 4.63 (dd, 1H, H5´, *J* = 3.5, 12 Hz), 4.52 (m, 1H, H4´), 3.44 (m, 2H, C$\underline{H_2}$OH), 2.43 (s, 3H, CH_3Ph), 2.42 (s, 3H, CH_3Ph), 2.29 (m, 1H, H2´), 2.21 (m, 2H, CH_2Ar), 2.13 (m, 1H, H2"), 1.52 (m, 2H, $CH_2C\underline{H_2}CH_2$). ^{13}C NMR (125.7 MHz, $CDCl_3$, ppm): 166.14, 166.12, 164.1, 150.4, 144.61, 135.4, 129.8, 129.7, 129.6, 129.5 , 129.4, 129.3, 126.5, 126.3, 115.1, 85.0, 82.3, 74.9, 64.2, 60.8, 60.4, 38.0, 32.0, 31.8, 22.7, 21.73, 21.67, 21.0, 14.2. IR (thin film); ν_{max} (cm^{-1}) 3495, 3191, 300, 2926, 1718, 1611, 1466, 1377, 1270, 1178, 1103, 1020. HRMS (ESI-MS) *m/z* calculated for $C_{28}H_{31}N_2O_8^+$ 523.2075: found 523.2099 $[M+H]^+$.

Alternative method for the resolution of anomers: To a solution of a purified mixture of anomers (2.0 g, 3.8 mmol) and imidazole (1.2 g, 17 mmol) in CH_2Cl_2 (38 mL) was added TBS-Cl (1.3 g, 8.4 mmol) while stirring. After 4h the solvent was removed in vacuo and the content was taken up in CH_2Cl_2 (50 mL) and washed with 3% (aq., w/v) $NaHCO_3$ (2 × 50 mL). The organic layer was dried over anhydrous Na_2SO_4, decanted and the solvent removed in vacuo. Purification was achieved by flash column chromatography using an EtOAc:hexanes solvent system (35:65, v/v) to afford 1.2 g (49 %) of the *β*-anomer and 0.68 g (28 %) of the *α*-anomer, both as colorless

foams. To a solution of the β-anomer of **3b** (0.50 g, 0.79 mmol) in THF (4 mL) was added 1 M TBAF (in THF, 1.2 mL, 1.2 mmol) which was heated to 45 ^{o}C with stirring. After 4 h the solvent was removed in vacuo and the content purified by flash column chromatography using a solvent system (1% → 3%, v/v) to afford 0.33 g (81 %) of **3b** a colorless foam.

3-(2´-Deoxy-3´-5´-*O*-bis(toluoyl)-β-D-ribofuranosyl)-5,6-dihydrofuro[2,3-*d*]pyrimidin-2(3*H*)-one (**4a**)

To a solution of compound **3a** (1.2 g, 2.3 mmol) in pyridine (25 mL) was added MsCl (240 μL, 3.1 mmol) dropwise at 0°C while stirring. After 5 h, the reaction was diluted with THF (300 mL) and DBU (1.2 mL, 8.0 mmol) was added and heated to a gentle reflux. After 16 h the solvent was removed in vacuo and the content was taken up in CH_2Cl_2 (100 mL), washed with brine (2 × 50 mL) and 3% (aq.) $NaHCO_3$ (2 x 50 mL). The organic layer was dried over anhydrous Na_2SO_4, decanted and the solvent was removed in vacuo to produce a yellow gum. The product was purified *via* flash column chromatography using a gradient of CH_2Cl_2:CH_3OH (1 % → 4 % increased by increments of 0.5 %, v/v) to afford 1.0 g (91%) of **4a** as a colorless foam. R_f (SiO_2 TLC): 0.45 CH_3OH:CH_2Cl_2 (5% , v/v). $\lambda_{max(MeCN)}$ = 241 nm. ^{1}H NMR (500 MHz, $CDCl_3$, ppm): δ 7.95 (d, 2H, Ar, *J* = 8.5 Hz), 7.87 (d, 2H, Ar, *J* = 8.5 Hz), 7.72 (s, 1H, H6), 7.31 - 7.23, (m, 4H, Ar), 6.41 (dd, 1H, H1´, *J* = 6.0, 14 Hz), 5.61 (m, 1H, H3´), 4.83 (m, 1H, H5´), 4.66 - 4.60 (m, 3H, H4´ and CH_2O), 4.55 (m, 1H, H5´´), 3.06 (m, 1H, H2´), 2.91 (m, 1H, CH_2Ar), 2.69 (m, 1H, CH_2Ar), 2.44 (s, 3H, CH_3Ph), 2.43 (s, 3H, CH_3Ph), 2.22 (m, 1H, H2´´). ^{13}C NMR (125.7 MHz, $CDCl_3$, ppm): 166.1, 166.0, 162.8, 150.2, 144.61, 144.60, 136.9, 129.9, 129.7, 129.6, 129.5, 129.39, 129.37, 129.3, 126.6, 126.3, 111.4, 85.0, 82.9, 74.9, 64.1, 42.6, 38.1, 30.4, 21.75, 21.73. IR (thin film); ν_{max} (cm^{-1}) 3196, 3061, 2923, 2851, 1718, 1611, 1577, 1465, 1377, 1270, 11178, 1101, 1020. HRMS (ESI-MS) *m/z* calculated for $C_{27}H_{27}N_2O_7^{+}$ 491.1813: found 491.1797 $[M+H]^{+}$.

3-(2´-Deoxy-3´-5´-*O*-bis(toluoyl)-β-D-ribofuranosyl)-3,5,6,7-tetrahydro-(2*H*)-pyro[2,3-*d*]pyrimidin-2-one (**4b**)

To a solution of compound **3b** (1.5 g, 2.9 mmol) in CH_2Cl_2 (30 mL) was added NEt_3 (0.80 mL, 5.74 mmol) which was allowed to stir at 0 ^{o}C for 15 min. MsCl (0.30 mL, 3.9 mmol) was added dropwise over 10 min and cooled to -20 ^{o}C for 1 h without stirring. The reaction was diluted with CH_2Cl_2 (100 mL) and washed with ice/water (50 mL), ice/brine (50 mL) and ice/3% aq. (w/v) $NaHCO_3$ (50 mL). The organic layer was then dried over anhydrous Na_2SO_4, decanted and the solvent was removed in vacuo to produce a yellow gum. The gum was dissolved in THF (400 mL), DBU (0.40 mL, 2.7 mmol) was added and the reaction was gently refluxed under stirring. After 3 h the solvent was removed in vacuo and the crude was taken up in CH_2Cl_2 (150 mL), washed with ice/brine (50 mL) and 3% aq. $NaHCO_3$ (w/v, 2 × 50mL). The organic layer was dried over anhydrous Na_2SO_4, decanted and the solvent was removed in vacuo to produce a yellow gum. Purification was achieved by flash column chromatography using a CH_3OH:CH_2Cl_2 solvent system (3 → 4 %, v/v) to afford 1.27 g (88%) of **4a** as a colorless foam. R_f (SiO_2 TLC): 0.33 CH_3OH:CH_2Cl_2 (6 %). $\lambda_{max(MeCN)}$ 239 nm. ^{1}H NMR (500 MHz, $CDCl_3$, ppm): 7.96 - 7.94 (d, 2H, Ar, *J* = 8.5 Hz), 7.87 - 7.85 (d, 2H, Ar, *J* = 8.5 Hz), 7.67 (s, 1H, H6), 7.28 - 7.24 (m, 4H, Ar), 6.41 (dd, 1H, H1´, *J* = 5.5, 8.0 Hz), 5.61 (m, 1H, H3´), 4.84 (dd, 1H, H5´, *J* = 4, 13 Hz), 4.64 - 4.60 (m, 2H, H4´ and H5´´), 4.33 (m, 1H, CH_2O), 4.26 (m, 1H, CH_2O), 3.10 (dd, 1H, H2´,

J = 5, 14.5 Hz), 2.44 (s, 3H, CH_3Ph), 2.42- 2.38 (m, 4H, 1H from CH_2Ar and CH_3Ph), 2.23 (m, 1H, H2´´), 2.15 (m, 1H, CH_2Ar), 1.87 (m, 1H, $CH_2\underline{CH_2}CH_2$), 1.79 (m, 1H, $CH_2\underline{CH_2}CH_2$). ^{13}C NMR (125.7 MHz, $CDCl_3$, ppm): 170.0, 166.2, 166.0, 155.3, 144.55, 144.52, 140.4, 129.8, 129.5, 129.41, 129.37, 129.3, 129.2, 126.5, 126.3, 101.4, 87.5, 83.7, 75.3, 68.5, 64.3, 39.6, 21.72, 21.69. 21.6, 21.5. IR (thin film); ν_{max} (cm^{-1}) 3357, 2923, 2851, 2362, 2336, 1719, 1667, 1611, 1520, 1337, 1271, 1103, 1020. HRMS (ESI-MS) *m/z* calculated for $C_{28}H_{29}N_2O_7^+$ 505.1969: found 505.1985 $[M+H]^+$.

3-(2´-Deoxy-5´-*O*-(4,4´-dimethoxytrityl)-*β*-D-ribofuranosyl)-5,6-dihydrofuro[2,3-*d*]pyrimidin-2(*3H*)-one (**5a**)

Compound **4a** (0.59 g, 1.2 mmol) was allowed to stir at 0 °C in a solution of saturated methanolic ammonia (250 mL) and gradually allowed to warm up to room temperature. After 5 h the solvent was removed in vacuo and the resulting residue was washed with Et_2O (2 × 25mL). The deprotected nucleoside was partially purified by flash column chromatography (using a short column) with a gradient of $CH_3OH:CH_2Cl_2$ (4 % →15 %, v/v) to elute the product as a colorless powder. This powder was taken up in pyridine (15 mL) and stirred at 0 °C for 15 min. To this solution was added DMAP (cat) and DMT-Cl (0.38 g, 1.1 mmol) in small portions over 20 min under stirring. After 16 h, the solvent was removed in vacuo and the content was taken up in CH_2Cl_2 (100 mL), washed with 3 % (aq., w/v) $NaHCO_3$ (2 × 100 mL) and brine (100 mL). The organic layer was dried over anhydrous Na_2SO_4, decanted and the solvent was removed in vacuo to produce a yellow gum. The product was purified *via* flash column chromatography using a gradient of $CH_3OH:CH_2Cl_2$ (2% → 4%, v/v) to afford 0.32 g (48%) of **5a** as a colorless foam. R_f (SiO_2 TLC):0.18 $CH_3OH:CH_2Cl_2$ (5%, v/v). $\lambda_{max(MeCN)}$ 283 nm. ^{1}H NMR (500 MHz, $CDCl_3$, ppm): 8.08 (s, 1H, H6), 7.35 (d, 2H, Ar, J = 7.5 Hz), 7.28 - 7.19 (m, 7H, Ar), 6.81 (d, 4H, Ar, J = 9 Hz), 6.28 (t, 1H, H1´, J = 6.0 Hz), 4.61 - 4.50 (m, 3H, H3´ and CH_2O), 4.07 (m, 1H, H4´), 3.78 (s, 6H, OCH_3), 3.48 (dd, 1H, H5´, J = 3, 11 Hz), 3.42 (dd, 1H, H5’, J = 3, 10.5 Hz), 2.69 - 2.62 (m, 2H, H2´ and 1H from CH_2Ar), 2.49 - 2.41 (m, 1H, CH_2Ar), 2.32 (m, 1H, H2’´´), 2.08 (d, 1H, OH, J = 4 Hz). ^{13}C NMR (125.7 MHz, $CDCl_3$, ppm): 177.9, 158.68, 158.67, 156.6, 144.5, 137.4, 135.3, 130.09, 130.06, 128.1, 128.0, 127.0, 113.29, 113.27, 103.9, 87.2, 86.9, 86.3, 71.7, 71.2, 63.0, 55.3, 42.3, 24.0. IR (thin film); ν_{max} (cm^{-1}) 3361, 2922, 2650, 1673, 1608, 1570, 1509, 1481, 1437, 1322, 1251, 1178, 1154, 1034. HRMS (ESI-MS) *m/z* calculated for $C_{32}H_{32}N_2O_7Na^+$ 579.2102: found 579.2100 $[M+Na]^+$.

3-(2´-Deoxy-5´-*O*-(4,4´-dimethoxytrityl)-β-D-ribofuranosyl)-3,5,6,7-tetrahydro-(*2H*)-pyro[2,3-*d*]pyrimidin-2-one (**5b**)

Compound **4b** (0.95 g, 1.9 mmol) was allowed to stir in a solution of saturated methanolic ammonia (500 mL) at 0°C and gradually allowed to warm to room temperature. After 5 h, the solvent was removed in vacuo and the resulting residue was washed with Et_2O (3 × 5 mL). The resulting gum was diluted with pyridine (18 mL) and stirred at 0 °C for 15 min. To this solution was added DMAP (cat) and DMT-Cl (0.38 g, 1.1 mmol) in pyridine (10 mL) dropwise over 25 min under stirring. The reaction was allowed to gradually warm up to room temperature. After 16h the solvent was removed in vacuo and the content was taken up in CH_2Cl_2 (50 mL) then washed with ice/brine (50 mL) followed by ice/3% (aq.) $NaHCO_3$ (w/v, 2 × 50 mL). The organic layer was then dried over anhydrous Na_2SO_4, decanted and the solvent was removed in vacuo to

produce a yellow-orange gum. The product was purified *via* flash column chromatography using a gradient of CH_3OH:CH_2Cl_2 (3% → 5%, v/v) to afford 0.39 g (36%) of **5b** as a colorless foam. R_f (SiO_2 TLC): 0.18 CH_3OH:CH_2Cl_2 (5%, v/v). $\lambda_{max(MeCN)}$ 282 nm. ^1H NMR (500 MHz, $CDCl_3$, ppm): δ 8.05 (s, 1H, H6), 7.38 (d, 2H, Ar, J = 7.5 Hz), 7.31 - 7.21 (m, 7H, Ar), 6.83 (d, 4H, Ar, J = 9 Hz), 6.32 (t, 1H, H1´, J = 6.0 Hz), 4.49 (m, 1H, H3´), 4.30 (m, 2H, CH_2O), 4.11 (m, 1H, H4´), 3.80 (s, 6H, OCH_3), 3.50 (dd, 1H, H5´, J = 3, 10.5 Hz), 3.38 (dd, 1H, H5´´, J = 3, 10.5 Hz), 2.70 (m, 2H, H2´), 2.35 (m, 1H, H2´´), 2.21 (d, 1H, OH, J = 3.5 Hz), 2.15 (m, 1H, CH_2Ar), 1.87 (m, 1H, CH_2Ar), 1.81 (m, 2H, $CH_2\underline{CH_2}CH_2$). ^{13}C NMR (125.7 MHz, d_6-DMSO, ppm): 170.0, 158.7, 155.0, 144.1, 142.1, 135.9, 135.7, 130.22, 130.16, 128.4, 128.1, 127.3, 113.8, 101.7, 86.4, 86.3, 86.2, 70.5, 68.7, 63.8, 55.54, 55.46, 41.5, 21.5, 21.2. IR (thin film); ν_{max} (cm^{-1}) 3360, 2921, 2850, 1661, 1632, 1522, 1509, 1454, 1336, 1250, 1177, 1117, 1033. HRMS (ESI-MS) *m/z* calculated for $C_{33}H_{35}N_2O_7^+$ 571.2439: found 571.2455 $[M+H]^+$.

3-(2’-Deoxy-3´-*O*-(β-cyanoethyl-*N*,*N*´-diisopropylphosphite)-5´-*O*-(4,4′-dimethoxytrityl)-*β*-D-ribofuranosyl)-5,6-dihydrofuro[2,3-*d*]pyrimidin-2(*3H*)-one (**6a**)

To a stirred solution of compound **5a** (0.25 g, 0.45 mmol) and DIPEA (0.147 mL, 0.844 mmol) in THF (4.5 mL) was added dropwise Cl-POCEN*i*Pr_2 (0.149 mL, 0.674 mmol). After 30 min, the solvent was evaporated in vacuo and the content was diluted with EtOAc (50 mL) then washed with 3% (aq., w/v) $NaHCO_3$ (2 × 50 mL) and brine (50 mL). The organic layer was dried over anhydrous Na_2SO_4, decanted and the solvent was removed in vacuo to produce a yellow gum. Purification was achieved *via* short flash column chromatography using EtOAc:hexanes (4:1, v/v) (with 0.1% NEt_3, v/v) as eluent to afford 0.25 g (74 %) of **6a** as a white foam. R_f (SiO_2 TLC): 0.40, 0.31 EtOAc/hexanes (4:1, v/v). $\lambda_{max(MeCN)}$ 283 nm. ^1H NMR (500 MHz, d_6-acetone, ppm): δ 8.14-8.09 (m, 1H, H6), 7.53-7.50 (m, 2H, Ar), 7.40 - 7.25 (m, 7H, Ar), 6.95-6.92 (m, 4H, Ar), 6.33-6.26 (m, 1H, H1´), 4.83-4.77 (m, 1H, H3´), 4.64-4.56 (m, 2H, CH_2O), 4.26-4.20 (m, 1H, H4´), 3.94-3.47 (m, 12H, CH_2OP, NCH, H5´, H5´´ and OCH_3), 2.92-2.58 (m, 5H, CH_2CN, CH_2Ar & H2´), 2.43-2.36 (m, 1H, H2´´), 1.33-1.14 (m, 12H, 4 × CH_3). ^{13}C NMR (125.7 MHz, d_6-acetone, ppm): 177.89, 177.88, 158.9, 155.7, 144.95, 144.93, 136.9, 136.8, 135.7, 136.6, 135.5, 135.4, 130.2, 130.1, 128.2, 128.1, 127.90, 127.89, 126.91, 126.88, 118.1, 118.0, 113.2, 103.9, 103.8, 86.74, 86.71, 86.5, 86.4, 85.5, 85.2, 73.2, 71.5, 62.9, 62.7, 58.72, 58,65, 58.6, 58.5, 58.2, 54.7, 45.0, 44.9, 43.1, 43.0, 40.7, 40.5, 24.1, 24.02, 24.00, 23.92, 23.89, 22.29, 22.29, 22.2, 19.91, 19.85, 19.8, 19.5, 19.4. ^{31}P NMR (202.3 MHz, d_6-acetone, ppm): 148.27, 148.11. IR (thin film); ν_{max} (cm^{-1}) 2967, 2929, 2362, 2337, 1678, 1570, 1509, 1322, 1250, 1179, 1034. HRMS (ESI-MS) *m/z* calculated for $C_{41}H_{50}N_4O_8P^+$ 757.3361: found 757.3385 $[M+H]^+$.

3-(2´-Deoxy-3´-*O*-(β-cyanoethyl-*N*,*N*´-diisopropylphosphite)-5´-*O*-(4,4´-dimethoxytrityl)-*β*-D-ribofuranosyl)-3,5,6,7-tetrahydro-(*2H*)-pyro[2,3-*d*]pyrimidin-2-one (**6b**)

To a stirred solution of compound **5b** (0.25 g, 0.44 mmol) and DIPEA (0.143 mL, 0.816 mmol) in THF (4.3 mL) was added dropwise Cl-POCEN*i*Pr_2 (0.146 mL, 0.656 mmol). After 30 min the solvent was evaporated in vacuo and the content was diluted in EtOAc (50 mL) then washed with 3 % (aq., w/v) $NaHCO_3$ (2 × 50 mL) and brine (50 mL). The organic layer was dried over anhydrous Na_2SO_4, decanted and the solvent was removed in vacuo to produce a yellow gum. Purification was achieved *via* short flash column chromatography using EtOAc (with 0.1 % NEt_3, v/v) as eluent to afford 0.25 g (74%) of **6b** as a colorless foam. R_f (SiO_2 TLC): 0.20, 0.14

EtOAc. $\lambda_{max(MeCN)}$ 283 nm. ^{1}H NMR (500 MHz, d_6-acetone, ppm): δ 8.04-8.00 (m, 1H, H6), 7.54-7.48 (m, 2H, Ar), 7.41 - 7.25 (m, 7H, Ar), 6.96-6.89 (m, 4H, Ar), 6.33-6.27 (m, 1H, H1´), 4.80-4.73 (m, 1H, H3´), 4.40-4.11 (m, 4H, CH_2O, H4´, 1H from CH_2OP), 3.94-3.47 (m, 11H, 1 H from CH_2OP, NCH, H5´, H5´´and OCH_3), 2.96-2.38 (m, 4H, CH_2CN, H2', H2"), 2.29-2.24 (m, 1H, CH_2Ar), 2.09-1.97 (m, 1H, CH_2Ar), 1.87-1.79 (m, 2H, $CH_2\underline{CH_2}CH_2$), 1.34-1.11 (m, 12H, 4 × CH_3). ^{13}C NMR (125.7 MHz, d_6-acetone, ppm): 169.9, 169.82, 169.80, 158.93, 158.92, 154.81, 154.79, 144.9, 144.77, 144.76, 141.5, 141.4, 141.3, 135.69, 135.66, 135.6, 135.5, 135.4, 130.20, 130.18, 103,16, 130.1, 128.22, 128.18, 128.0, 127.91, 127.90, 127.0, 126.9, 113.23, 113.18, 101.4, 101.2, 101.1, 87.0, 86.72, 86.69, 86.3, 86.1, 85.6, 68.3, 68.2, 62, 9, 54.7, 43.1, 43.0, 24.1, 24.0,23.9, 22.31, 22.29, 21.50, 21.48, 21.3, 19.9, 19.4, 19.30, 19.27. ^{31}P NMR (202.3 MHz, d_6-acetone, ppm): 148.30, 148.03. IR (thin film); ν_{max} (cm^{-1}) 2968, 2927, 2849, 1736, 1669, 1510, 1455, 1337, 1250, 1179, 1118, 1035. HRMS (ESI-MS) *m/z* calculated for $C_{42}H_{52}N_4O_8P^+$ 771.3517: found 771.3542 $[M+H]^+$.

Solid phase synthesis and purification of oligonucleotides.

All DNA sequences were synthesized using an Applied Biosystems Model 3400 synthesizer on a 1.5 μmol scale employing standard *β*-cyanoethyl phosphoramidite cycles supplied by the manufacturer with modifications to certain coupling times described below. Commercially available 3´-*O*-2´-deoxynucleoside phosphoramidites, containing "fast" deprotecting groups, were dissolved in anhydrous acetonitrile at a concentration of 0.1 M. The modified 3´-*O*-2´-deoxynucleoside phosphoramidites (**6a** and **6b**) were dissolved at a concentration of 0.12 M. Assembly of sequences began with detritylation (3% trichloroacetic acid (TCA) in CH_2Cl_2, v/v), followed by phosphoramidite coupling: Commercially available 3´-*O*-2´-deoxyphosphoramidites for 120 s and modified phosphoramidites (**6a** and **6b**) for 600 s. Capping with phenoxyacetic anhydride-pyridine-tetrahydrofuran (1:1:8; solution A) and 1-methyl-1*H*-imidazole-tetrahydrofuran (16:84 w/v; solution B) then oxidation (0.02 M iodine in tetrahydrofuran-water-pyridine, 2.5:2:1, v/v/v) followed every coupling. Removal of the 5´-terminal trityl group on the oligonucleotide was carried out on the synthesizer.

The oligomer-bound CPG support was removed from the column and transferred into screw cap microfuge tubes fitted with Teflon lined caps. The oligonucleotides containing the modifications 3-(2´-deoxypentofuranosyl)-5,6-dihydrofuro[2,3-*d*]pyrimidin-2(3*H*)-one (**DFP**) and 3-(2´-deoxypentofuranosyl)-3,5,6,7-tetrahydro-2*H*-pyrano[2,3-*d*]pyrimidin-2-one (**TPP**) were deprotected and cleaved from the solid support with mild deprotection conditions using freshly prepared anhydrous K_2CO_3 in CH_3OH (0.05 M) for 3.5 h at room temperature in the dark. The K_2CO_3 was neutralized with an equimolar amount of acetic acid prior to being transferred into clean vials to separate the solution from the CPG. The CPG was rinsed twice with 250 μL aqueous CH_3CN (50%, v/v). The crude DNA was dried down in a centrifugal lypophilizer ("Speed vac") and then desalted using C-18 SEP PAK cartridges (Waters) prior to purification.

All oligonucleotide sequences were purified from pre-terminated products by strong anion exchange (SAX) HPLC using a Dionex DNAPAC PA-100 column (0.4 cm × 25 cm) purchased from Dionex (Sunnyvale, CA) with a linear gradient of 0-52% buffer B, v/v, over 24 min (buffer A: 100 mM Tris HCl, pH 7.5, 10% acetonitrile and buffer B: 100 mM Tris HCl, pH 7.5, 10%

CH_3CN, 1 M NaCl) at 55 ^oC. The columns were monitored at 260 nm for analytical runs and/or 280 nm for preparative runs. The purified oligomers were desalted using C-18 SEP PAK cartridges (Waters).

Oligonucleotide characterization by ESI-MS and nuclease digestion.

Mass spectra for the oligonucleotides were acquired at the Concordia University Centre for Biological Applications of Mass Spectrometry (CBAMS) using a Micromass Qtof2 mass spectrometer (Waters) equipped with a nanospray ion source. The mass spectrometer was operated in full scan, negative ion detection mode and the raw data were deconvoluted.

The oligomers (0.05 A_{260} units / 0.38 nmol) were characterized by enzymatic digestion (snake venom phosphodiesterase: 0.28 units and calf intestinal phosphatase: 5 units) in buffer consisting of 10 mM Tris HCl, pH 8.1, and 2 mM $MgCl_2$ for 48 h at 37 ^oC. The resulting mixture of nucleosides was analyzed by reversed phase HPLC using a Symmetry® C-18 5 µm column (4.6 mm × 150 mm, Waters). A linear gradient of 0-70% buffer B (v/v) over 30 min was used to elute the analytes (buffer A: 50 mM sodium phosphate, pH 5.8, 2% CH_3CN (v/v) and buffer B: 50 mM sodium phosphate, pH 5.8, 50% CH_3CN (v/v)). The identity of the nucleosides was verified by co-injection with the corresponding standards and eluted at the following times: dC (4.3 min), dG (6.7 min), dT (7.4 min), dA(8.5 min), modified bicyclic pyrimidyl nucleosides (7.7 min for the **DFP,** and 9.1 min for the **TPP** nucleosides), and the ratio of unmodified nucleosides was determined. The bicyclic pyrimidyl nucleosides showed poor absorbance at 260nm and improved absorbance at 300 nm. As a result, absorption of the modified nucleosides was monitored at 300 nm and their presence confirmed by an independent injection of the deprotected analogues of **4a** and **4b**, respectively. The molecular weights of the modified oligomers were determined by ESI-QToF MS and these were in agreement with the calculated values.

UV thermal denaturation studies of DNA duplexes.

Molar extinction coefficients for the modified oligonucleotides were calculated using the nearest-neighbour approximations (M^{-1} cm^{-1}) of the mononucleotides and dinucleotides. The molar extinction coefficients for thymidine were used for the modified nucleotides. All duplexes were prepared by mixing equimolar amounts of the interacting strands (0.5 A_{260} unit of the strand containing the modification / 3.8 nmol) and lyophilizing the mixture to dryness in a centrifugal concentrator under vaccum. The resulting pellet was dissolved in 90 mM NaCl, 10 mM sodium phosphate, and 1 mM EDTA buffer (pH 7.0) to give a final concentration of 3.8 µM duplex. Before the thermal run, samples were degassed in a centrifugal concentrator for 1 min. Samples were held at 90 ^oC for 5 min to ensure duplex melting. Annealing profiles were acquired at 260 nm at a rate of cooling of 0.5 ^oC min^{-1}, from 90 to 15 ^oC, using a Varian CARY Model 3E spectrophotometer equipped with a 6-sample thermostated cell block and a temperature controller. Samples were held at 15 ^oC for 2 min and re-heated at 0.5 ^oC min^{-1} to 90 ^oC showing reversibility (data not shown). The data were analyzed according to the published procedure from Puglisi and Tinoco[6] and transferred to Microsoft Excel™ software.

Circular dichroism (CD) spectroscopy of DNA duplexes.

Circular dichroism spectra were acquired on a Jasco J-815 spectropolarimeter equipped with a Julaba F25 circulating bath as previously reported.[7] The spectra are an average of 5 scans acquired at a rate of 20 nm min^{-1}, with a bandwidth of 1 nm and a sampling wavelength of 0.2 nm in fused quartz cells (Starna 29-Q-10). Scans were performed between 350 and 220 nm at 15 °C. The molar ellipticity [θ] was calculated from the equation, θ = ε/Cl, where ε is the relative ellipticity (mdeg), C is the molar concentration of the DNA duplex (M), and l is the path length (cm).

Molecular modeling of DNA duplexes.

Molecular modeling was performed with the Hyperchem 7.5 software package from Hypercube utilizing the AMBER force field. Hybridized oligomers containing a dT·dA, ***O*^{4}-MedT**·dA, ***O*^{4}-EtdT**·dA, **DFP**·dA, and **TPP**·dA base pair were constructed from the nucleic acid template option using a B-form duplex. Duplexes were solvated with water using a periodic box occupying about 3 times the volume of the duplex alone. Standard Amber99 parameters were used with the dielectric set to constant. “One to four scale factors” non-bonded interactions were set to 0.5 (both electrostatic and van der Waals). Cutoffs were applied to “switched” to an outer and inner radius of 13.5 and 9.5 Å, respectively. All structures were geometry optimized using Polak-Ribiere conjugate gradient until the RMS gradient was less than 0.1 kcal/(Å mol) using the periodic boundary condition option.

Steady-state kinetics with hPol *η*.

The 5´-FAM-labeled primer (5´-TCGTAAGCGTCAT-3´) and template DNA (3´-AGCATTCGCAGTAXTACT-5´, where X denotes the modified nucleotide) were mixed in a 1:1 molar ratio and annealed by heating to 90 °C, followed by slow cooling. Steady-state experiments were conducted with typical hPol *η* concentrations of 1.9-7 nM and 5 µM template-primer duplex substrates in 40 mM Tris HCl buffer (pH 7.5) containing 100 mM KCl, 5mM $MgCl_2$, 10 mM DTT, 5% glycerol (w/v), and 100 µg/ml bovine serum albumin (BSA), to which was added varying concentrations of a single dNTP (added last to initiate the reaction). Experiments were carried out at 37 °C and reactions were typically run for 5 to 15 min in order to keep product formation below 20% of the oligonucleotide substrate concentration.

Reactions were terminated with a quench solution containing formamide, EDTA, bromophenol blue and xylene cyanol, and aliquots were applied to an 18% (w/v) acrylamide/7.5 M urea gel and separated by electrophoresis.[8] Fluorescence in the substrate and product primer bands was monitored and quantified using a Typhoon system (GE Healthcare Life Sciences, Pittsburgh, PA) and the data were fit to hyperbolic plots (Michaelis-Menten equation) using the program GraphPad Prism (GraphPad, La Jolla, CA).

LC-MS/MS Analysis of Full-length Extension Products.

The 5´-FAM-labeled primer (5´-TCGTAAGCGUCAT-3´) and template (3´-AGCATTCGCAGTAXTACT-5´ where X denotes the modified nucleotide) were annealed as described above. 2´-Deoxyuridine (U) was included in the primer for facile cleavage of the product to a shorter oligonucleotide (by treatment with uracil DNA glycosylase followed by hot

piperidine) that could be analyzed with an ion-trap mass spectrometer, as previously described.[9,10,11,12,13] DNA primer extension was accomplished by mixing hPol η (76 pmol, 0.95 μM) with template-primer duplex (2 nmol, 10 μM) and a mixture of 1 mM each of dATP, dCTP, dGTP, and dTTP at 37 °C for 1-1.5 h in 50 mM Tris-HCl buffer (pH 7.5), 50 mM NaCl, 5 mM DTT, 5 mM $MgCl_2$ and 50 μg/mL bovine serum albumin (BSA). The reactions were terminated by spin column separations to extract the dNTPs and Mg^{2+}. The extent of the reaction was determined by electrophoresis/fluorography prior to LC-MS analysis. The resulting product was then treated with 25 units of uracil DNA glycosylase and 0.25 M piperidine.[9,10,11,12,13] To identify the products, the resulting reactions were analyzed by LC-MS/MS using an Acquity UPLC system (Waters) interfaced to a Thermo-Finnigan LTQ mass spectrometer (Thermo Scientific, San Jose, CA) equipped with a negative ion electrospray source. Chromatographic separation was achieved with an Acquity UPLC BEH octadecylsilane (C18) column (2.1 mm × 100 mm, 1.7 μm). The LC solvent system was as follows: Mobile phase A, 10 mM $CH_3CO_2NH_4$ in 98% H_2O; mobile phase B, 10 mM $CH_3CO_2NH_4$ in 90% CH_3CN (v/v). The following gradient (v/v) was used with a flow rate of 300 μL min^{-1} at a temperature of 50 °C: Linear gradient from 0-3% B (v/v) in 3 min, followed by a linear increase to 20% B (v/v) from 3-5 min, then 20-100% B (v/v) from 5-6 min which is held for 2 min. The column was re-equilibrated for 3 min with 100% A (v/v). MS conditions were as follows: Source voltage, 4 kV; source current 100 μA; capillary voltage,- 49 V; capillary temperature, 350 °C; tube lens voltage, -90 V. Product ion spectra were recorded over the range *m/z* 300-2000 and the most abundant species (-2 charge) was used for collision-induced dissociation (CID) analysis. The calculation for the oligonucleotide sequence CID fragmentation was carried out using the Mongo Oligo Mass Calculator v2.06 from The RNA Institute (College of Arts and Science, University at Albany State University of New York). The relative yields of various products were calculated based on the peak areas of extracted ion chromatograms from LC-MS analyses. The sum of the peak areas was used for multicharged species.

Pre-steady-state experiments

The 5´-FAM-labeled primer (5´-TCGTAAGCGUCAT-3´) and template (3´-AGCATTCGCAGTAXTACT-5´ where X denotes the modified nucleotide) were annealed. Each annealed DNA substrate (1 μM) was mixed with hPol η (500 nM) in 40 mM Tris-HCl buffer (pH 7.5) containing 10 mM DTT, 0.1 mg/ml BSA, 5% glycerol (v/v), and 100 mM KCl (Solution A). Solution B contained 1 mM dATP or dGTP and 10 mM $MgCl_2$. The two solutions were mixed rapidly in a KinTek RP-3 instrument (KinTek Corp., Austin, TX) at 37 °C for 0.005 – 1 s (the final reaction concentration for hPol η was 250 nM) and stopped by the addition of 0.5 M EDTA. The products were separated with 18% denaturing polyacrylamide gels (w/v) and visualized with a Typhoon system (GE Healthcare). After quantitation using ImageJ software, the results were fit to a burst equation using GraphPad Prism: $y = A\,(1 - e^{-k_pt}) + k_{ss}E_ot$, where A is the burst amplitude, representing the apparent concentration of the active form of the enzyme, k_p is the burst rate, k_{ss} is the steady-state rate, and E_o is the total enzyme concentration.[14]

Crystallization

Crystals were obtained by the hanging drop vapor diffusion technique at 18 °C. O^4-Alkylthymidine modified DNA solutions were prepared by mixing template and primer strands

in a 1:1 molar ratio and annealing the mixture in the presence of 10 mM sodium HEPES buffer (pH 8.0), 0.1 mM EDTA, and 50 mM NaCl at 85 °C for 5-10 min, followed by slow cooling to room temperature. hPol *η* protein was mixed with the DNA duplex in a 1:1.2 molar ratio in the presence of 50 mM Tris-HCl, pH 7.5, containing 450 mM KCl, and 3 mM DTT, followed by addition of either 5 µL of 100 mM $MgCl_2$ or 5 µL of 100 mM $CaCl_2$. Using a spin concentrator with Amicon cutoff filter (Millipore, Billerica, MA), the complex was concentrated to a final concentration of ~2 mg/mL. Either dTTP or one of the non-hydrolyzable nucleoside triphosphate analogs was added to the concentrated mixtures containing Ca^{2+} or Mg^{2+}. The ternary complex solution was mixed with equal volume of reservoir solution containing 0.1 M MES (pH 5.5), 5 mM $MgCl_2$, and 16-22% (w/v) PEG 2000 monomethyl ether (MME) and equilibrated against 500 µL reservoir solutions. Crystals typically appeared after overnight incubation and were allowed to grow for one to a few weeks. They were transferred to cryoprotectant solution containing reservoir solution along with 25% glycerol (v/v), and then frozen in liquid nitrogen for data collection.

X-ray Diffraction Data Collection, Structure Determination and Refinement

X-ray diffraction data were collected at 100 K either on the 21-ID-F or the 21-ID-G beamline of the Life Sciences Collaborative Access Team (LS-CAT) at the Advanced Photon Source (APS), Argonne National Laboratory (Argonne, IL). All data were processed with the program HKL2000.[15] Data collection statistics are summarized in **Supplementary Table S4**. All structures were determined by Molecular Replacement in PHASER,[16,17] using the coordinates of the complex between hPol *η* and native DNA (PDB ID code 4O3N)[9] as the search model. Structures were refined either using PHENIX[18] or Refmac[16,19] and model building was carried out in COOT[20]. Model statistics and geometric parameters are summarized in **Supplementary Table S4**. Illustrations were generated with the program UCSF Chimera.[21]

Supplementary Discussion

Synthesis and characterization of nucleosides.

The incorporation of *O*^{4}MedT and *O*^{4}EtdT in DNA oligomers has been described previously.[22] The chemical synthesis for phosphoramidites **6a** and **6b** is shown in **Supplementary Scheme S1**. Precursors **1a** and **1b** were prepared according to published procedures.[1,2,3,4,5] Modified heterocycles **2a** and **2b** were obtained by a base-catalyzed intramolecular reaction under protic conditions.[2,3] Attachment of heterocycles **2a** or **2b** to commercially available 1-(α)-chloro-3,5-di-*O*-(*p*-toluoyl)-2-deoxy-D-ribose (Berry and Associates) by an uncatalyzed Vorbrüggen-type coupling yielded compounds **3a** and **3b** in moderate yields.[23] Resolution of the mixture of anomers for the latter coupling was challenging. Column chromatography using solvent systems such as CH_3OH:CH_2Cl_2, EtOAc:hexanes, and CH_3CN:CH_2Cl_2 with shallow gradients afforded low, if any, resolution of the anomeric mixture. The desired *β*-anomer was resolved by recrystallization from boiling EtOAc (1:1 EtOAc:hexanes, v/v for **3a** and 100% EtOAc for **3b**). Resolution of the anomers could also be accomplished by first protecting the free alcohol functionality with a *tert*-butyldimethylsilyl (TBS) group, followed by column chromatography (EtOAc:hexanes mobile phase) to separate the anomers. Subsequent removal of the TBS group supplied pure **3a** and **3b**. The bicyclic pyrimidines **4a** and **4b** were synthesized by first mesylating the terminal hydroxyl group followed by intramolecular cyclization in dilute basic conditions under reflux. A saturated solution of ammonia in methanol removed the toluoyl groups followed by protection of the 5´-hydroxyl group with DMTr to yield mono-protected nucleosides **5a** and **5b**, which were then phosphitylated with *N*,*N* diisopropylaminocyanoethylphosphonamidic chloride in the presence of diisopropylethylamine to produce phosphoramidites **6a** and **6b**. ^{31}P NMR signals were observed at 148.27, 148.11 ppm for **6a**, and 148.30, 148.03 ppm for **6b**, indicating the presence of the non-hydrolyzed phosphoramidite group. NMR and MS data for all small molecules can be found in **Supplementary Figures S1 - S32**.

Synthesis of DNA containing single modification inserts.

The solid-phase syntheses of **DFP** and **TPP** containing oligonucleotide sequences were performed on a 1.5 μmol scale using either phosphoramidite **6a** or **6b**, respectively. Given the potential lability of the lesions, as seen with other *O*^{4}-alkyl pyrimidine adducts[7], “fast-deprotecting” 3´-*O*-2´-deoxyphosphoramidites were employed (dissolved to a concentration of 0.1 M in anhydrous acetonitrile) with phenoxyacetic anhydride used as the capping agent to prevent an undesired N-acetylation reaction that could occur with acetic anhydride.[24] Phosphoramidites **6a** or **6b** were dissolved in acetonitrile to a concentration of 0.12 M with a coupling time of 600 s (as opposed to 120 s) to ensure optimal production of the desired full-length oligonucleotides.

An ultra-mild oligonucleotide deprotection protocol was required due to the labile nature of the *O*^{4}-alkylpyrimidine adducts.[7] These conditions employed treatment of the support bound oligonucleotide with a 0.05M solution of K_2CO_3 in anhydrous CH_3OH for 3.5 h at room temperature in the dark. The resulting crude DNA solution was neutralized with acetic acid to prevent oligonucleotide damage during the evaporation of the solvent in the centrifugal

concentrator. The resulting pellet was taken up in NaOAc (0.1M), desalted, and reconcentrated. The DNA sequences were purified by SAX-HPLC (see **Supplementary Figure S33** for the HPLC chromatographs) and subsequently desalted. ESI mass spectrometry of representative oligonucleotides $\mathbf{S_{DFP}}$ and $\mathbf{S_{TPP}}$ (with the sequence 5'-GGCTXGATCACCAG-3' where X is **DFP** and **TPP** for $\mathbf{S_{DFP}}$ and $\mathbf{S_{TPP}}$, respectively) revealed they had molecular weights of 4276.3 and 4290.8 Da (expected 4276.8 and 4289.9 Da), consistent with the expected values (see **Supplementary Figures S34** and **S35** for the mass spectra). For longer deprotection time under the same conditions, ESI-MS revealed the formation of a product with a mass 32 Da higher than expected. This was most-likely due to the attack of the methoxide nucleophile on the bicyclic pyrimidine moieties during the deprotection step. The composition of $\mathbf{S_{Fur}}$ and $\mathbf{S_{Pyr}}$ was further confirmed by enzymatic digestion to their constituent nucleosides with a mixture of snake venom phosphodiesterase and calf intestinal phosphatase followed by C_{18} RP-HPLC analysis (see **Supplementary Figures S36** and **S37** for the chromatographs). The presence of the **DFP** or **TPP** nucleosides was established by the appearance of one additional peak to the four standard nucleosides, which eluted from the HPLC column with a retention time of 7.7 min for the **DFP** nucleoside and 9.1 min for **TPP** nucleoside. The later elution of the **TPP** relative to the **DFP** nucleoside can be attributed to the presence of additional methylene linkage in the former, which increases its hydrophobicity. UV scans of **DFP** and **TPP** revealed that they had a reduced absorbance at 260 nm (the wavelength at which absorbance is monitored by the HPLC detector to detect the elution of nucleosides from the C_{18} column) relative to the four other nucleosides. Thus the C_{18} RP-HPLC experiments were simultaneously monitored at both 260 and 300 nm for the modified and standard nucleosides.

UV thermal denaturation of DNA duplexes and mismatch studies.

The influence of the **DFP** and **TPP** modifications on duplex stability was assessed by UV thermal denaturation experiments involving oligonucleotides $\mathbf{S_{DFP}}$ and $\mathbf{S_{TPP}}$ hybridized to complementary DNA (see **Supplementary Figure S38**). The melting curves of the duplexes containing **DFP** or **TPP** were sigmoidal with a comparable hyperchromicity between them that was slightly higher relative to the control duplex. The melting temperatures observed for duplexes containing **DFP** or **TPP** with adenine as the base pairing partner on the complementary strand were 48 and 49 ^{o}C, respectively, a significant reduction relative to the control duplex (62°C). Duplexes containing O^4-methylthymidine or O^4-ethylthymidine modifications (O^4MeT and O^4EtT) were found to have melting temperatures of 51 and 52 ^{o}C, respectively, comparable to the destabilizing influence of O^4-alkylated dT in DNA duplexes that has been reported previously.[25,26] The similar T_m values of duplexes containing **DFP** or **TPP** versus O^4MeT or O^4EtT suggest that the rigidity of the bicyclic pyrimidine system bears little impact on thermal stability relative to adducts with free rotation around the C4-O^4 bond. O^4-MedT has been shown, through NMR[27] and computational studies[28], to adopt a wobble base pairing motif when paired with a dA residue. Given the similar trends observed in the T_m values, it is reasonable to expect that **DFP** and **TPP** can engage in similar hydrogen bond interactions when paired with adenine.

The influence of other base pairings involving the **DFP** and **TPP** modifications were also studied by UV thermal denaturation experiments. The T_m values of duplexes containing these modifications were equivalent to O^4MedT or O^4EtdT when mispaired against cytosine or thymine. An increase in T_m was observed for all the O^4-alkylated thymidine analogs with guanine

as the base pairing partner relative to the other pairings. These findings for duplexes containing the bicyclic **DFP** and **TPP** modifications are consistent with studies involving *O*4-alkylated dT·dG mispairs.[22,25] Interestingly, previous studies with duplexes containing *O*4MedT and *O*4EtdT modifications reported biphasic thermal profiles with purine mispairs.[25,29] However, this behavior was not observed in this study, perhaps due to differences with the sequence investigated. Cruzeiro-Hansson and Goodfellow's computational analysis showed that the methyl group preferred to adopt the *syn* conformation for the *O*4-MedT·dG pair.[28] In this orientation, there are three hydrogen bonds between guanine and methylated thymine, namely that of the N^2H-O^2 (strong), N1H-O^2 (weak), and N1H-N3 (weak), respectively. The bicyclic constructs of **DFP** and **TPP** inherently lock the methylene group in the *anti* conformation, however, hydrogen bonding interactions between the same atoms as the *O*4-MedT·dG pair is possible.

Circular dichroism spectroscopy and molecular modeling of DNA duplexes.

CD spectra of duplexes composed of **DFP** and **TPP** paired against adenine on the complementary strand were acquired at 15 ^{o}C. The spectra (see **Supplementary Figure S39**) displayed typical B-form DNA signatures with a wavelength maximum of 280 nm, an intercept at approximately 260 nm, and a minimum around 250 nm, similar to the DNA duplex control (containing a dT·dA base pair). This suggests that the presence of the bicyclic pyrimidine moiety had a minimal influence on the global DNA structure, similar to what has been observed previously in NMR studies of duplexes containing *O*4-MedT.[27]

Hybridized oligomers containing a dT·dA, *O*4MedT·dA, *O*4EtdT·dA, **DFP**·dA or **TPP**·dA pair were constructed using Hyperchem® 7.5 software package starting from the canonical B-form duplexes. These were subject to geometry optimization using AMBER force field (see **Supplementary Figures S40** and **S41** for structures of the duplexes with the water molecules omitted for clarity and views of the modified and flanking base pairs from the top). Both optimized structures containing the *O*4MedT·dA and *O*4EtdT·dA base pairs had very similar features with the alpha carbon adopting a *syn* conformation with respect to the N3-atom, in agreement with published structural data by NMR and results from computational studies.[27,28] The **DFP** and **TPP** modifications behaved similarly to the *O*4MedT and *O*4EtdT. The duplexes exhibited minor global structural perturbations from the control duplex, namely retaining key features of B-form DNA. On a local level, the **DFP** and **TPP** modifications engaged with only one hydrogen bond contact between the N6H of adenine and O^4 of the modified pyrimidine at the Watson-Crick face. The modified pyrimidine bases were slightly shifted towards the major groove of the duplex to accommodate the bulk of the bicyclic construct. These slight distortions could explain the decrease in T_m values and increase in hyperchromicity changes observed. In general, the molecular model of **DFP** and **TPP** modifications do not seem to induce any gross geometrical distortions on the global structure, as corroborated by the circular dichroism experiments.

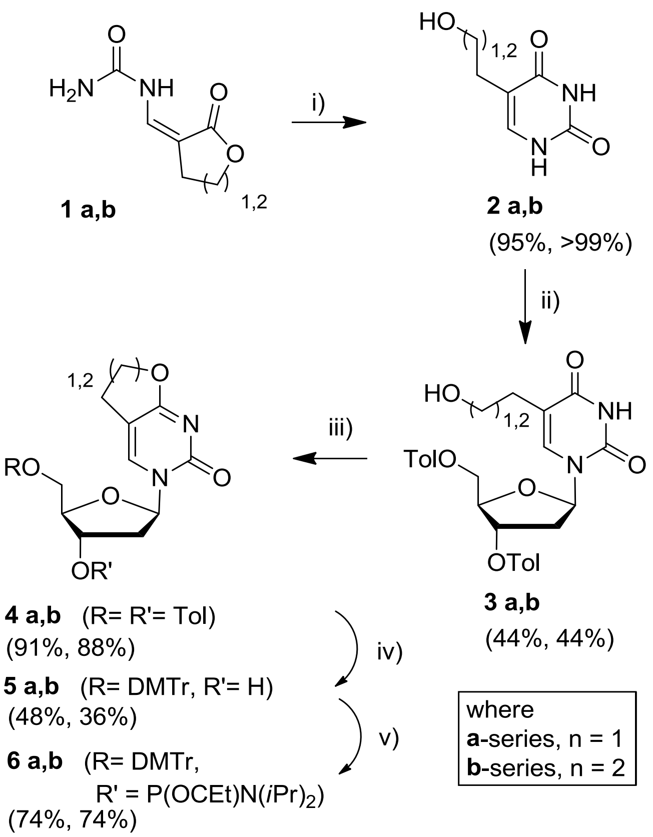

Supplementary Scheme S1. Reagents and conditions for **a**-series: i) NaOEt, EtOH, reflux. ii) 1. HMDS, TMS-Cl. 2. 1-(α)-chloro-3,5-di-*O*-(p-toluoyl)-2-deoxy-D-ribose, 1,2-dichloroethane. 3. TBAF (1 M in THF). iii) 1. Ms-Cl, Py 2. DBU, THF, reflux. iv) NH_3/CH_3OH (sat. solution). 2. DMTr-Cl, DMAP, pyridine. v) Cl-P(OCEt)N(iPr)$_2$, DIPEA, THF. Reagents and conditions for **b**-series: Identical to a-series except step iii) 1. Ms-Cl, NEt_3, CH_2Cl_2. 2. DBU, THF, reflux.

Supplementary Figure S1 - 500 MHz ^{1}H NMR spectrum of compound (**2a**) (in d_6-DMSO)

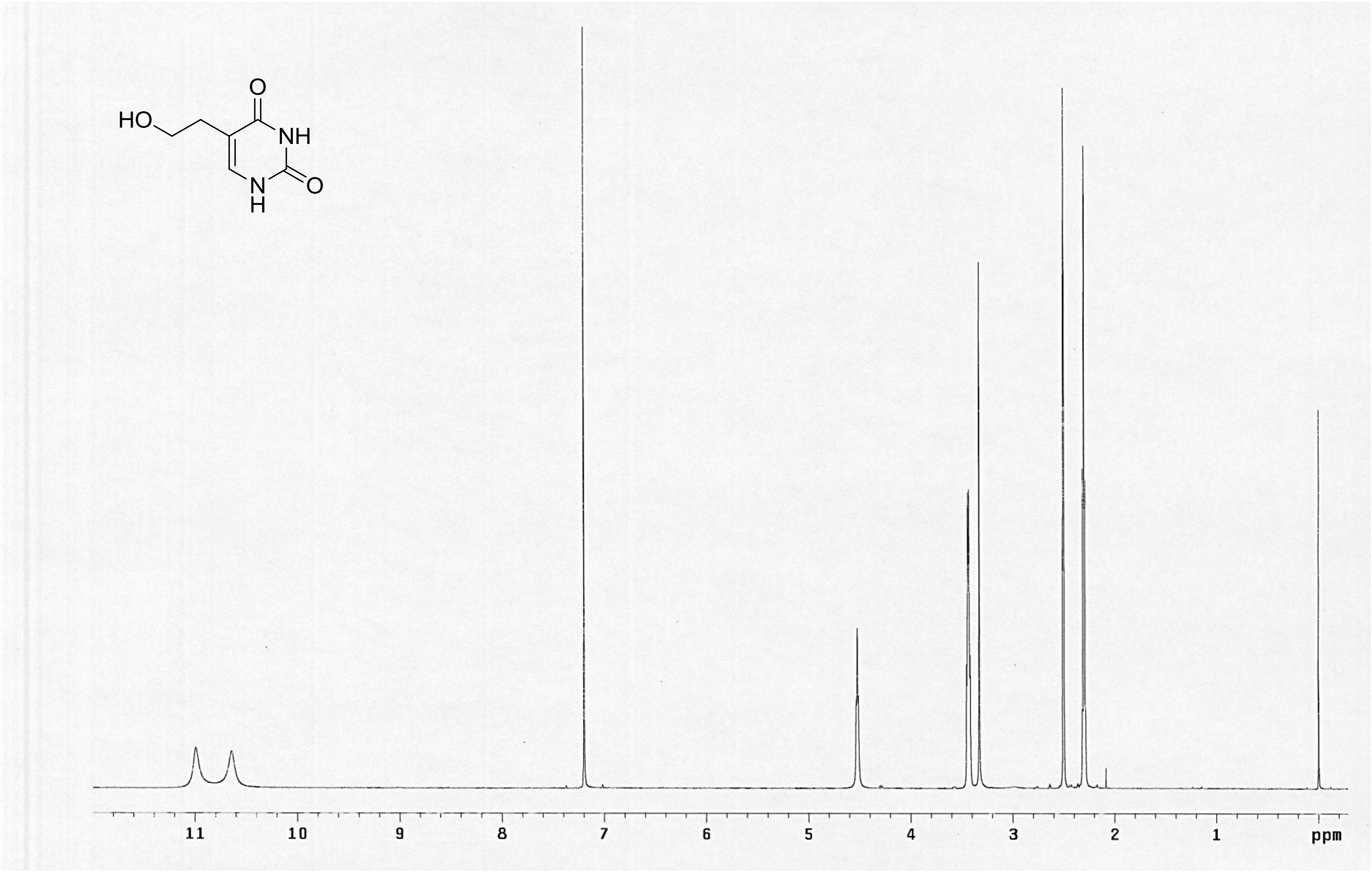

Supplementary Figure S2 - 125.7 MHz ^{13}C NMR spectrum of compound (**2a**) (in d_6-DMSO)

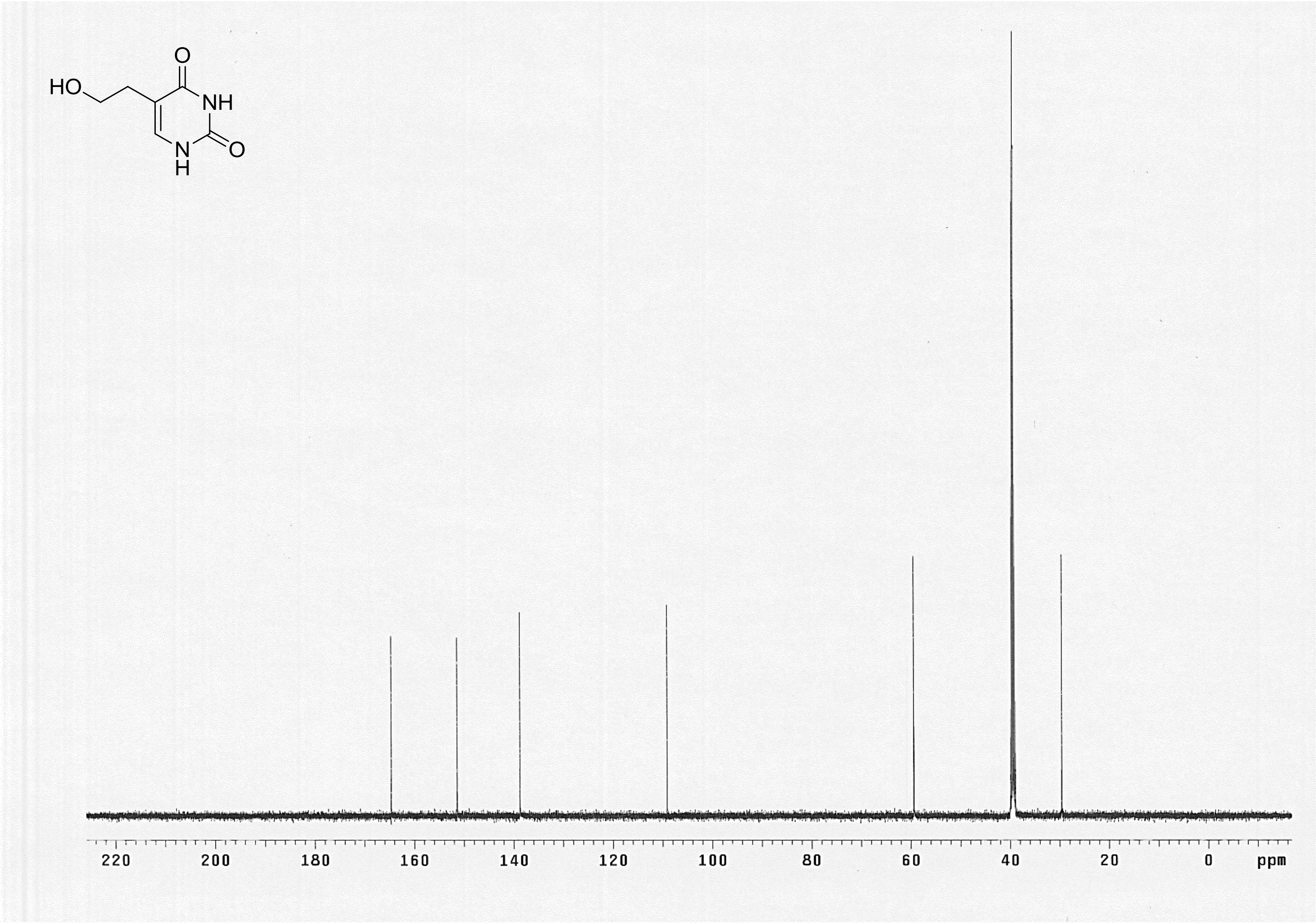

Supplementary Figure S3 - HR ESI-MS spectrum of compound (**2a**)

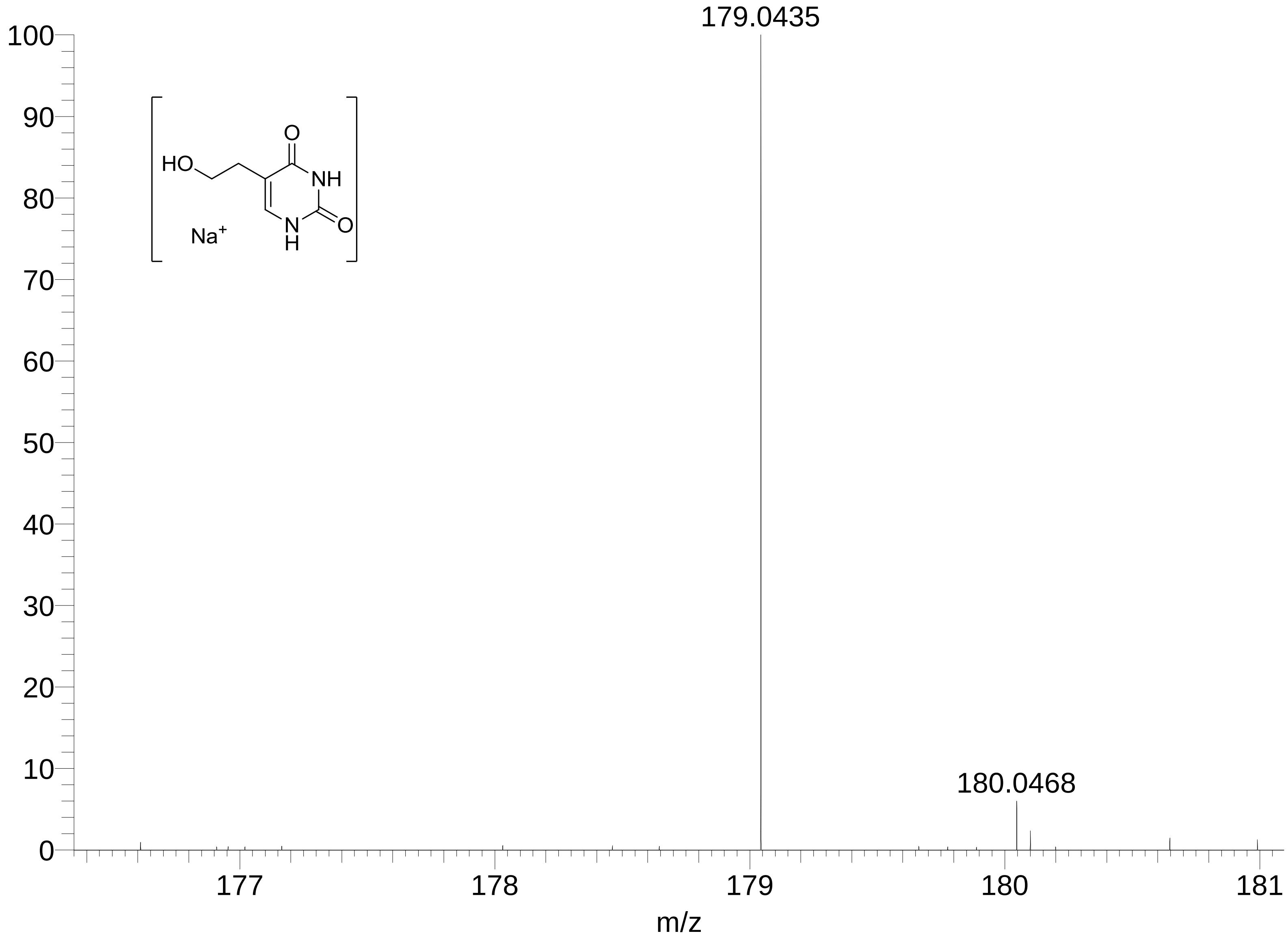

Supplementary Figure S4 - 500 MHz ^{1}H NMR spectrum of compound (**2b**) (in d_6-DMSO)

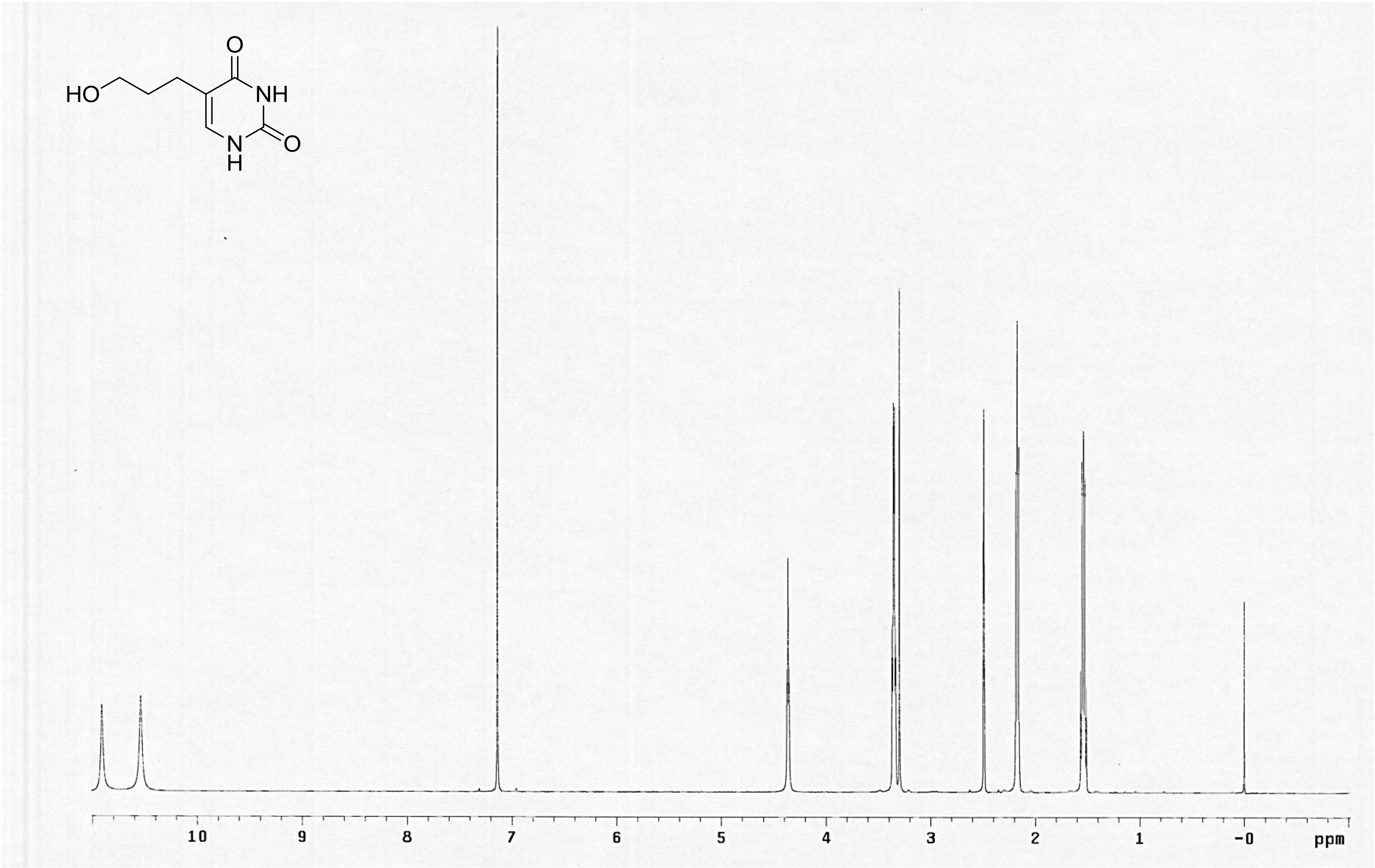

Supplementary Figure S5 - 125.7 MHz ^{13}C NMR spectrum of compound (**2b**) (in d_6-DMSO)

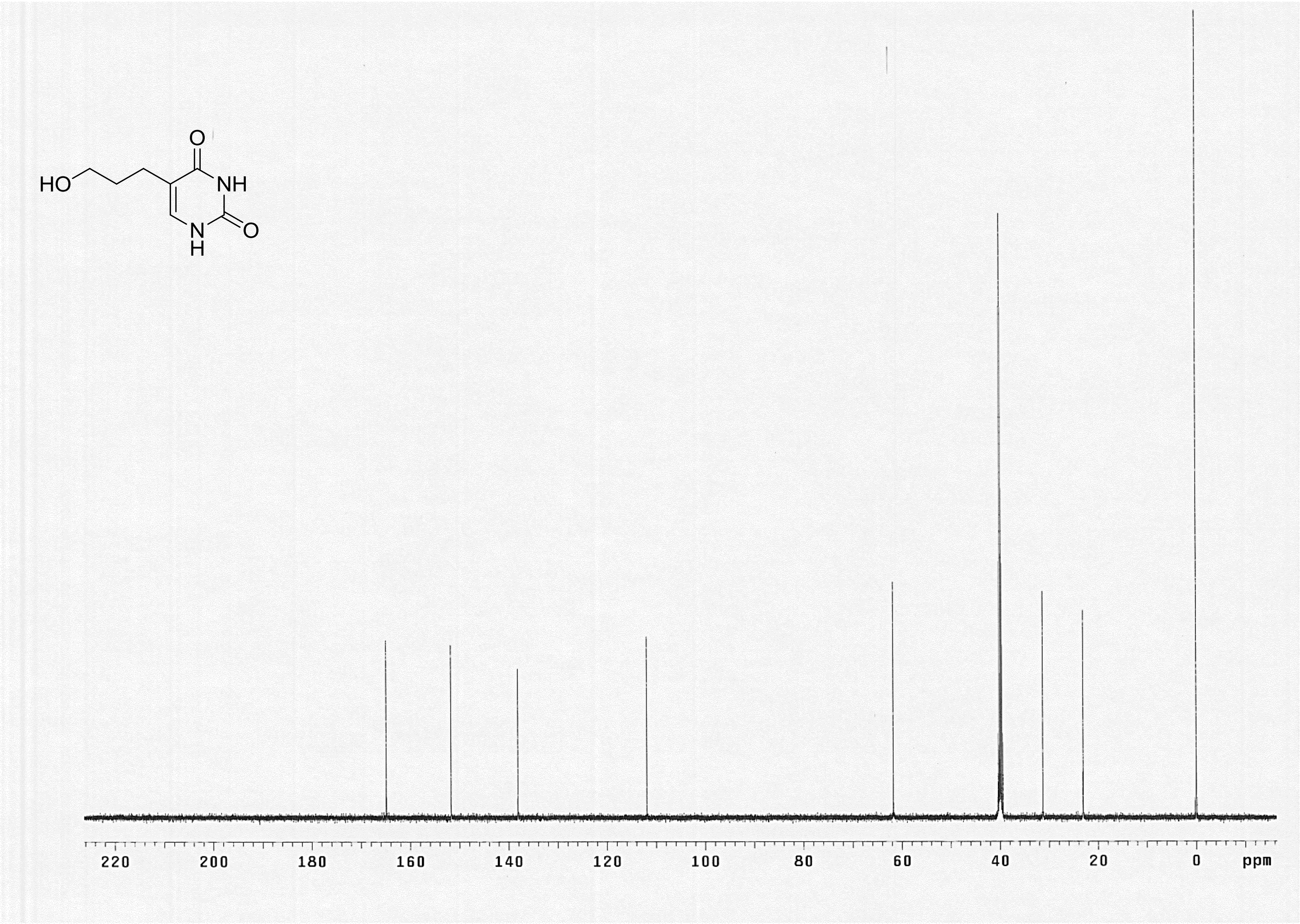

Supplementary Figure S6 - HR ESI-MS spectrum of compound (**2b**)

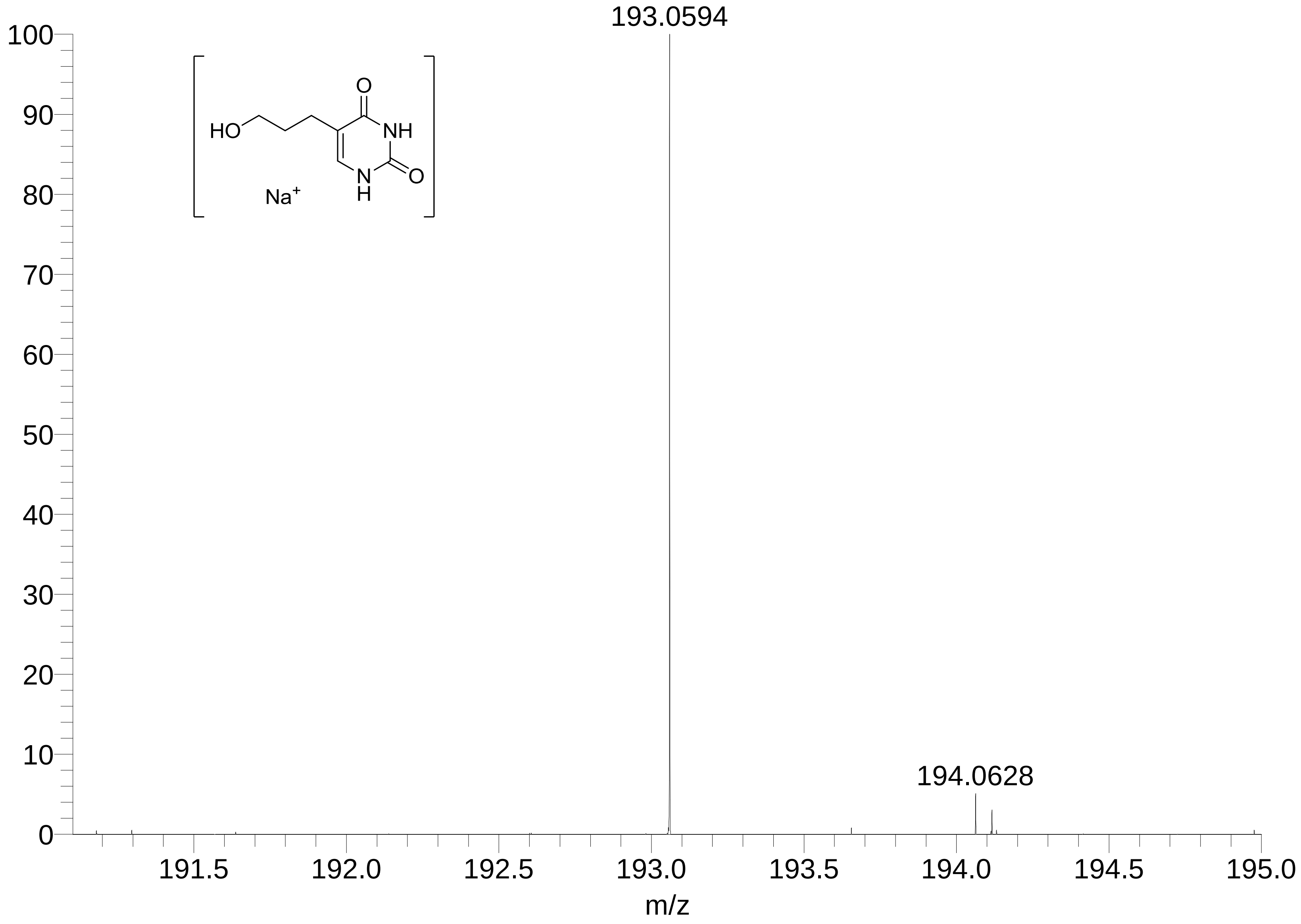

Supplementary Figure S7 - 500 MHz ^{1}H NMR spectrum of compound (**3a**) (in d_6-DMSO)

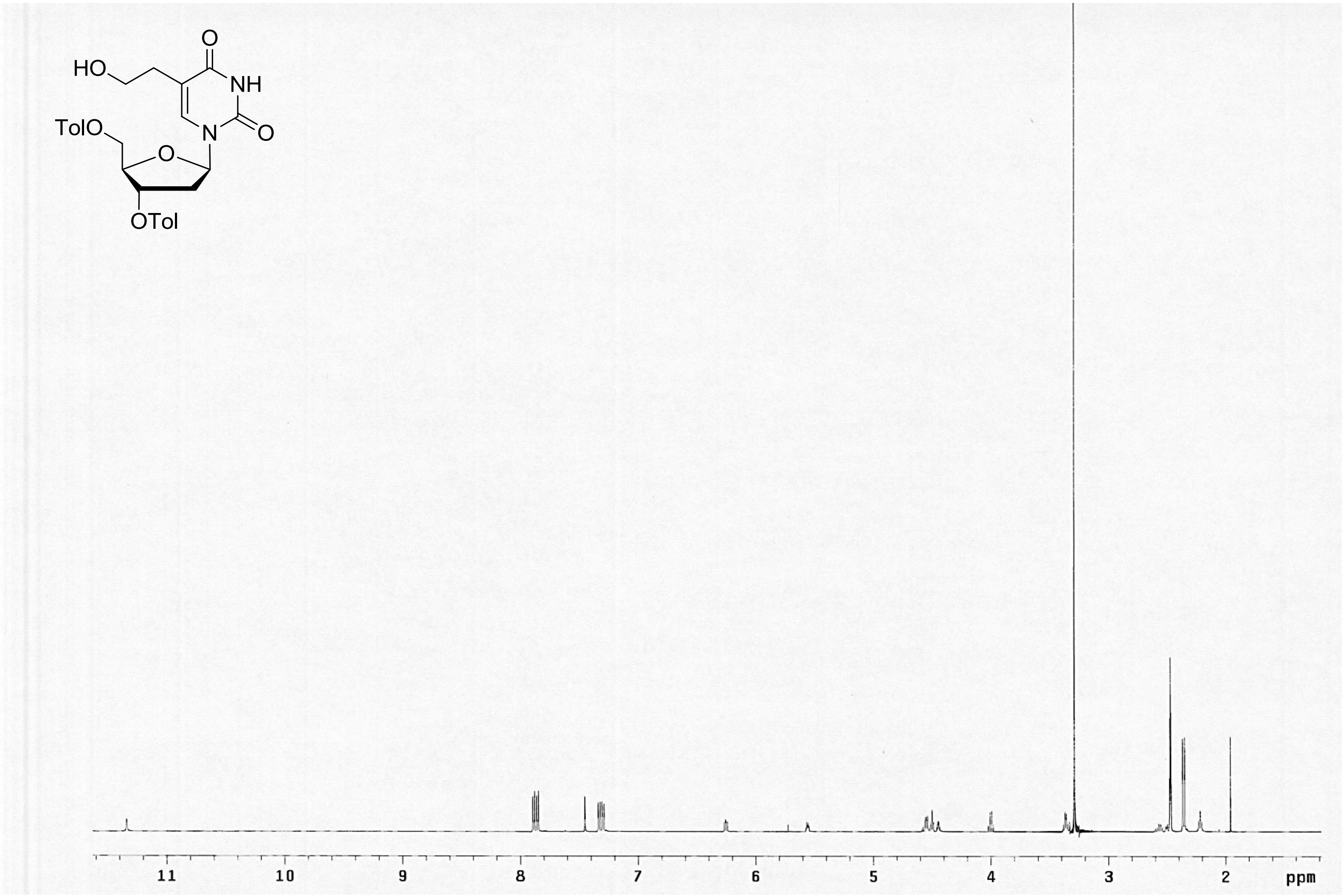

Supplementary Figure S8 - 125.7 MHz ^{13}C NMR spectrum of compound (**3a**) (in $CDCl_3$)

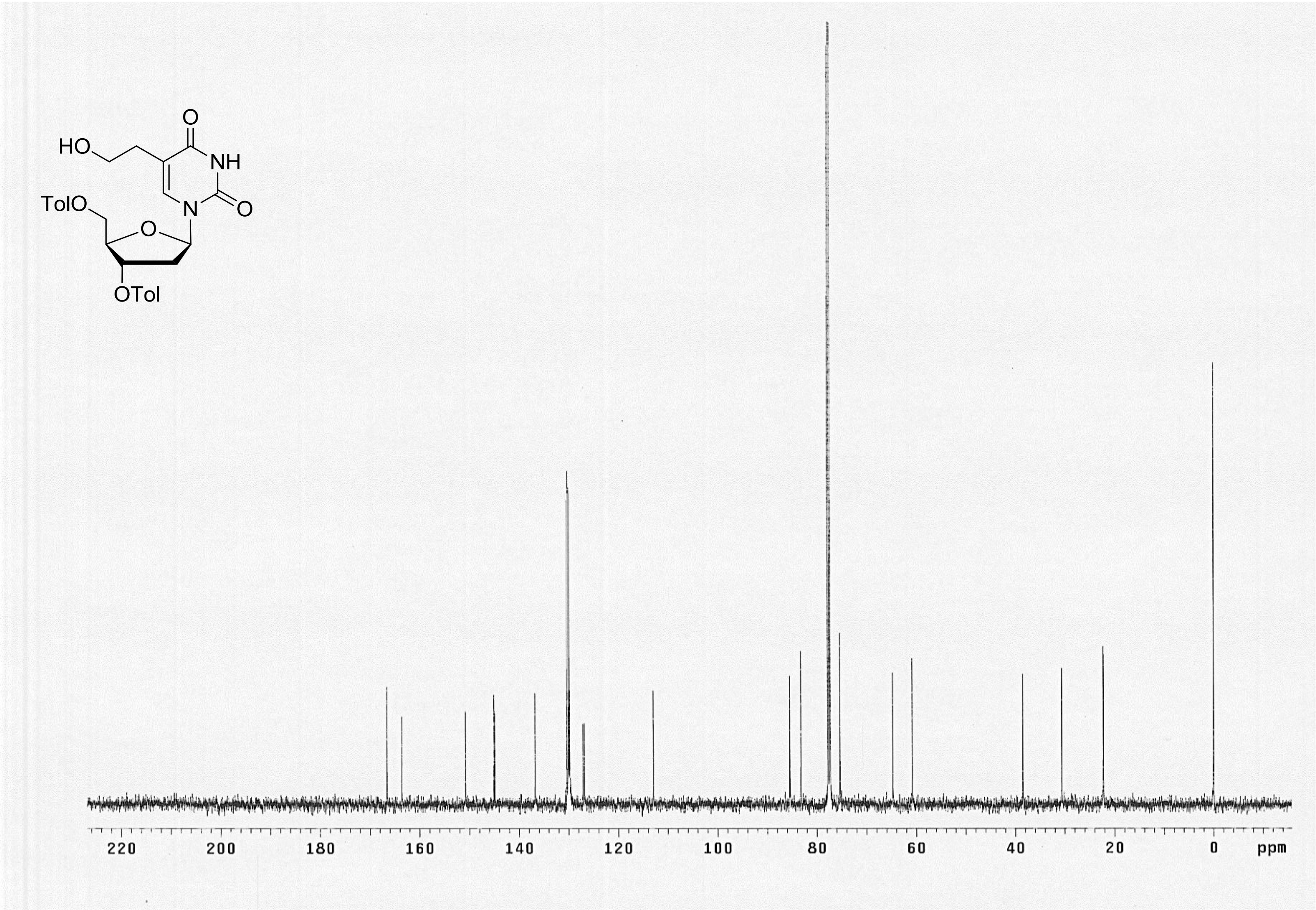

Supplementary Figure S9 - HR ESI-MS spectrum of compound (**3a**)

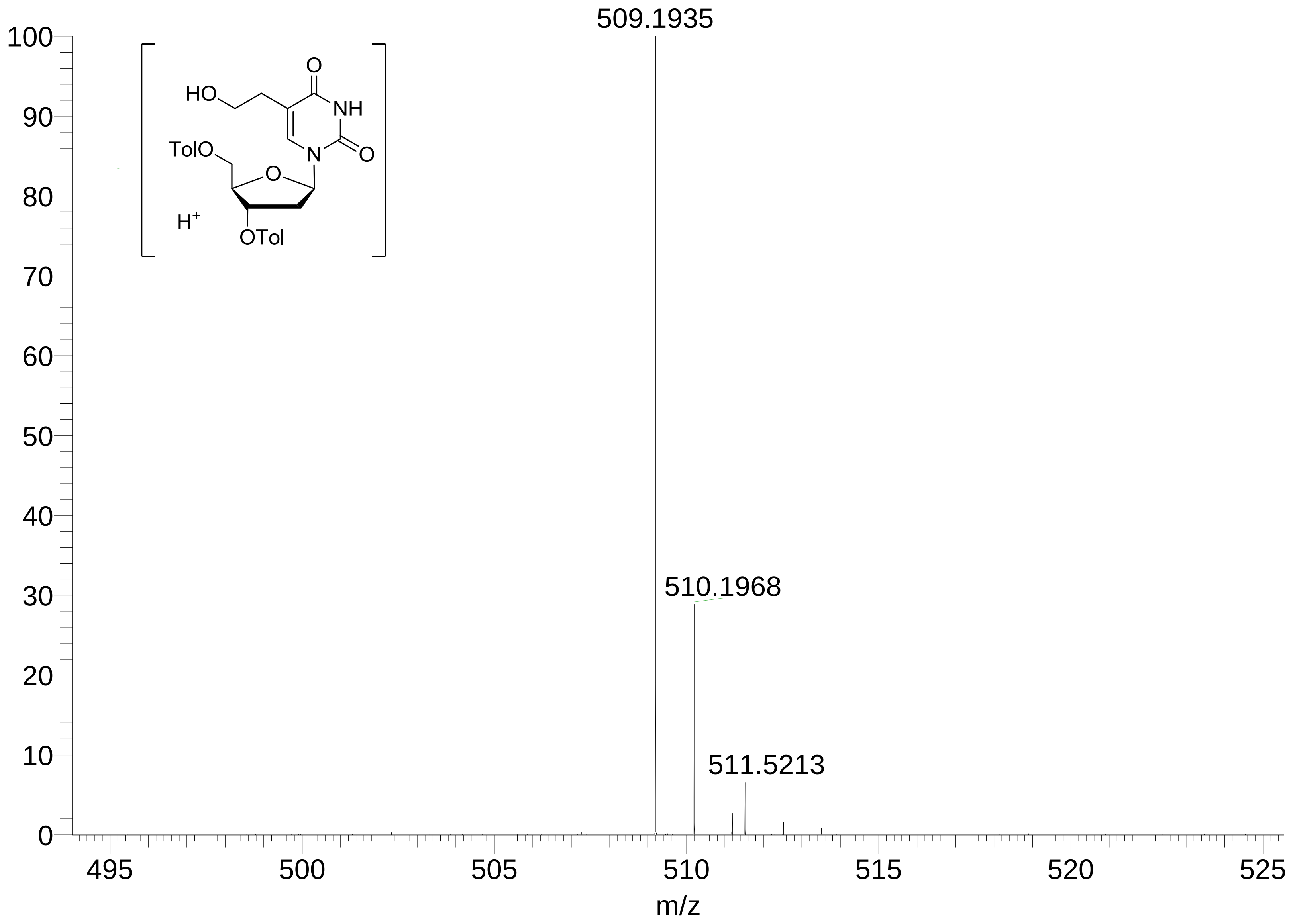

Supplementary Figure S10 - 500 MHz ^{1}H NMR spectrum of compound (**3b**) (in $CDCl_3$)

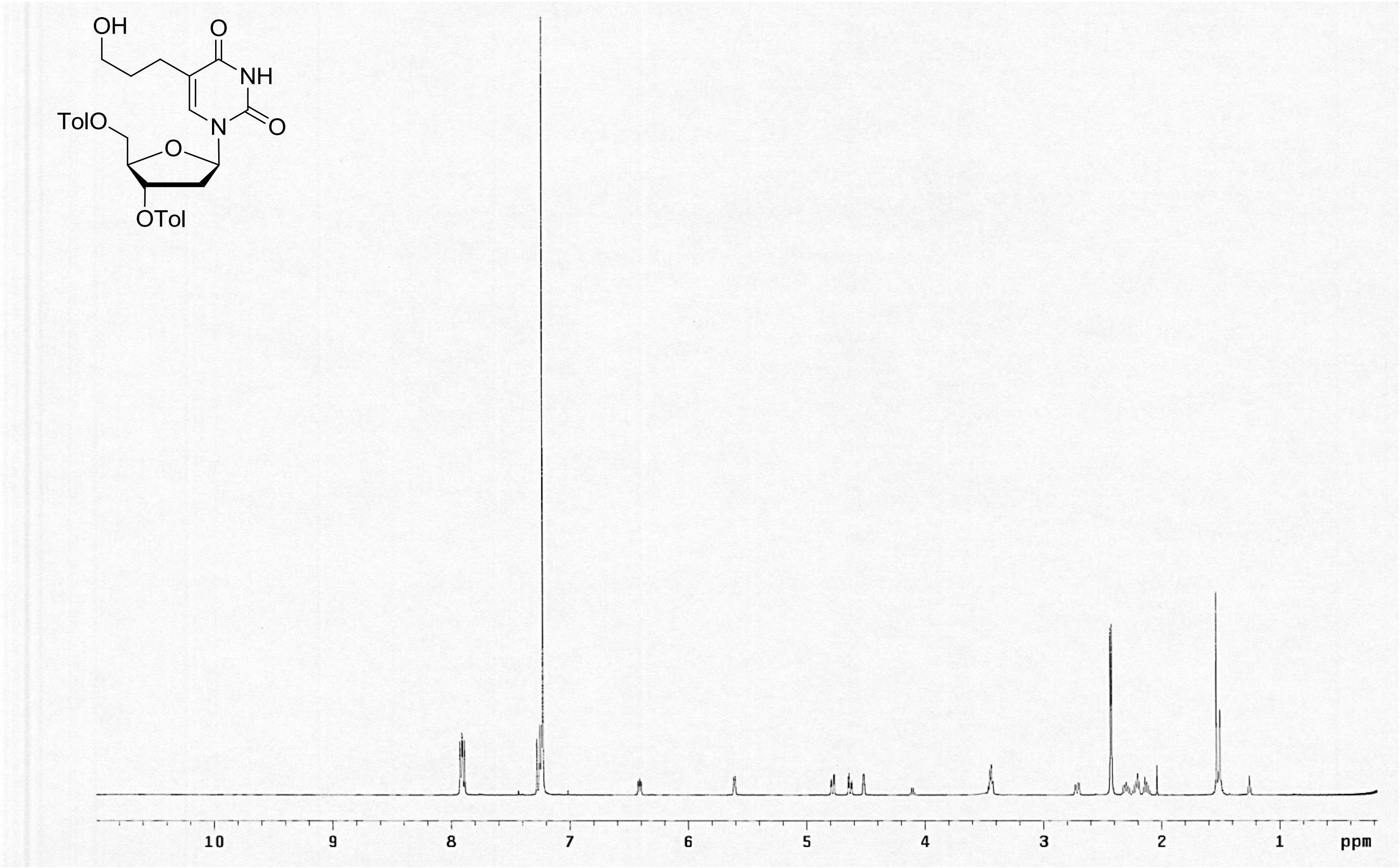

Supplementary Figure S11 - 125.7 MHz ^{13}C NMR spectrum of compound (**3b**) (in $CDCl_3$)

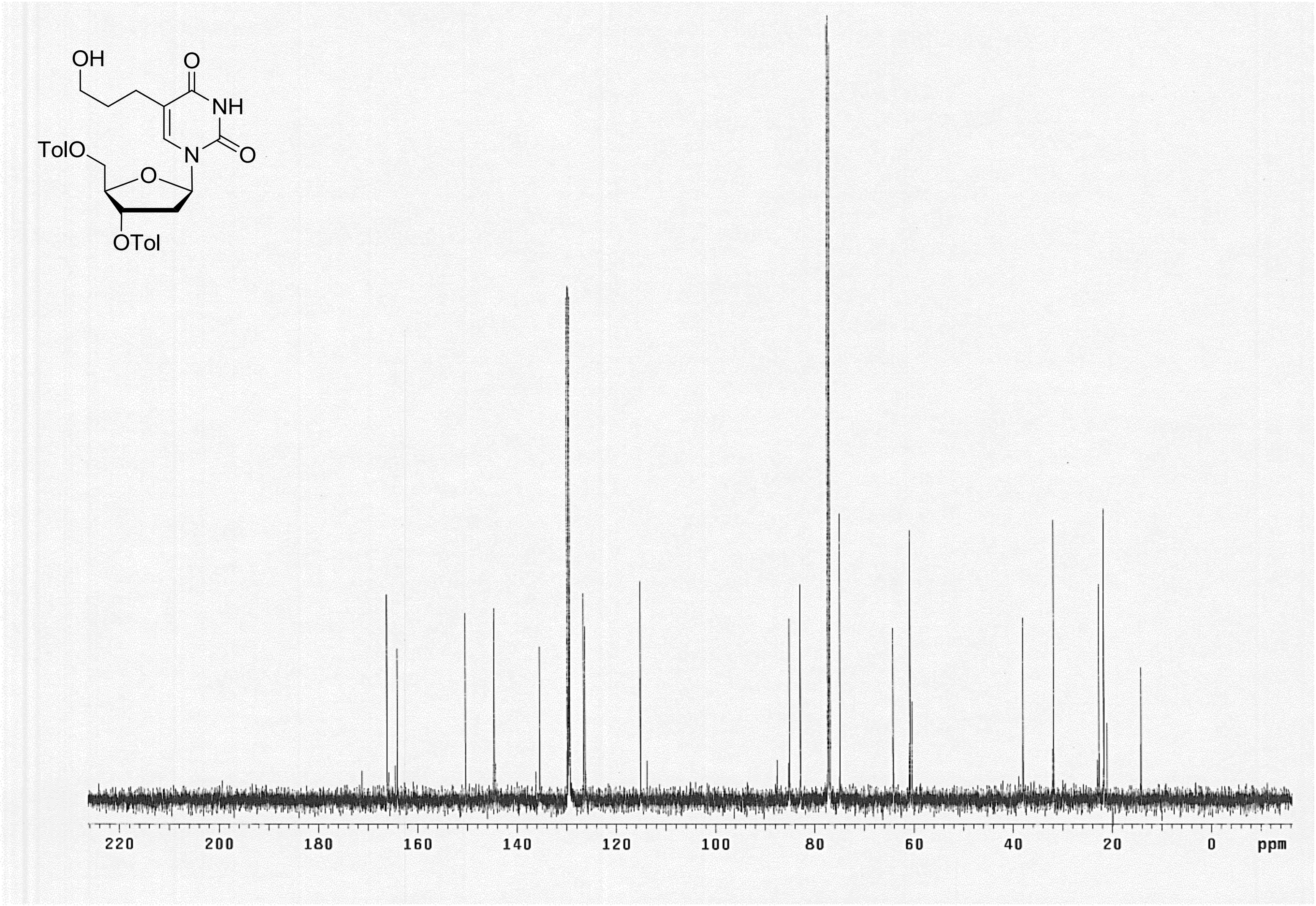

Supplementary Figure S12 - HR ESI-MS spectrum of compound (**3b**)

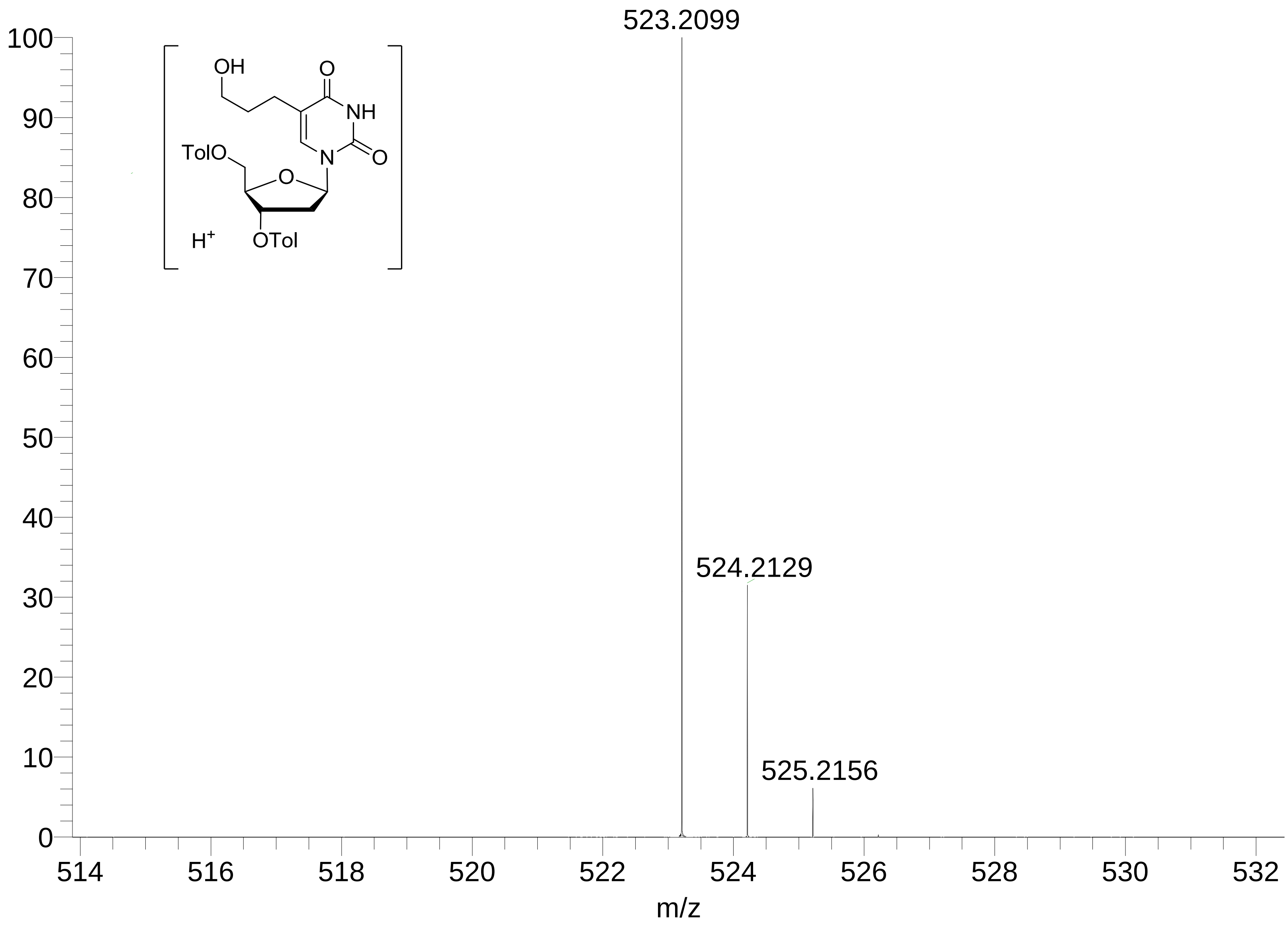

Supplementary Figure S13 - 500 MHz ^{1}H NMR spectrum of compound (**4a**) (in $CDCl_3$)

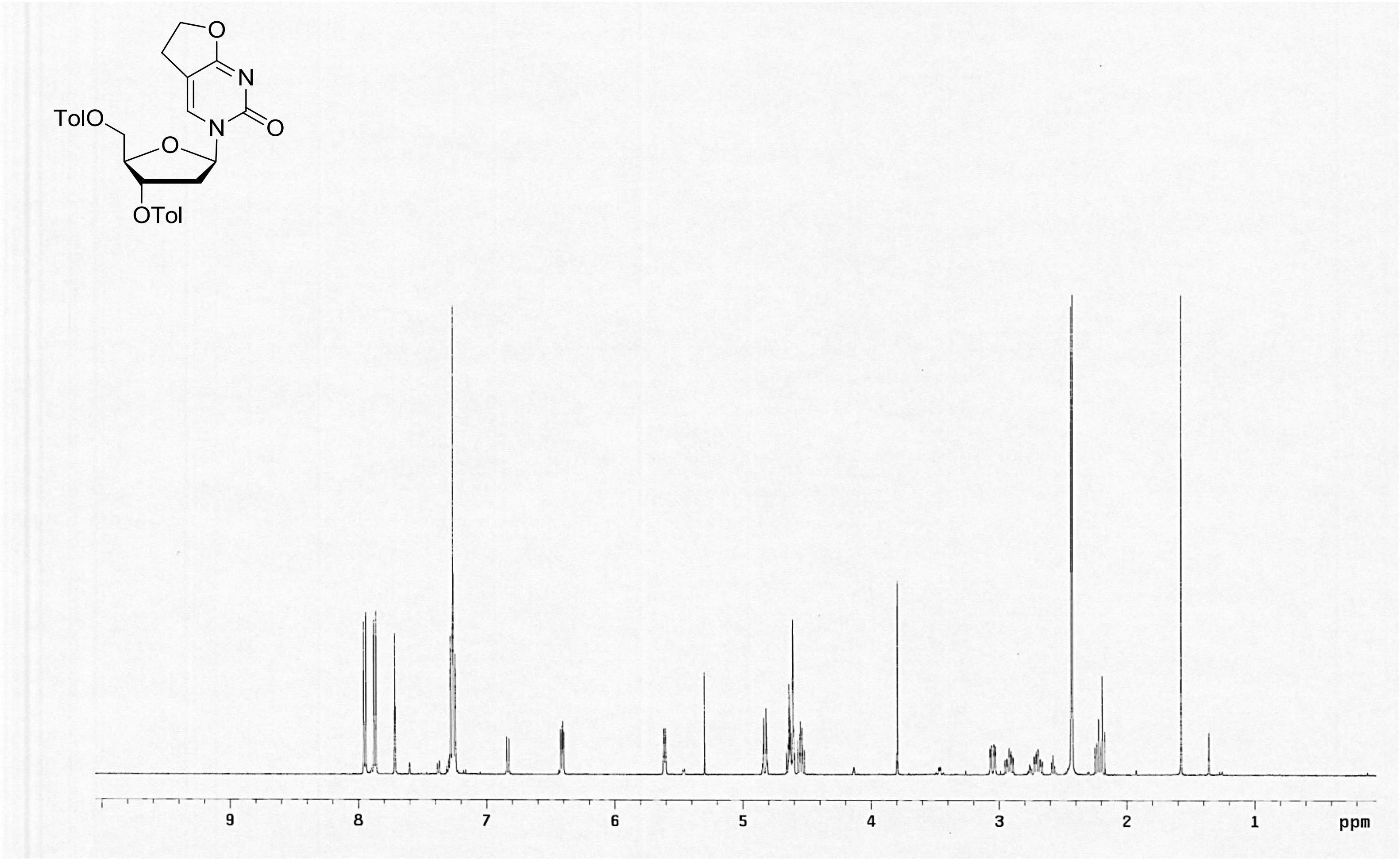

Supplementary Figure S14 - 125.7 MHz ^{13}C NMR spectrum of compound (**4a**) (in $CDCl_3$)

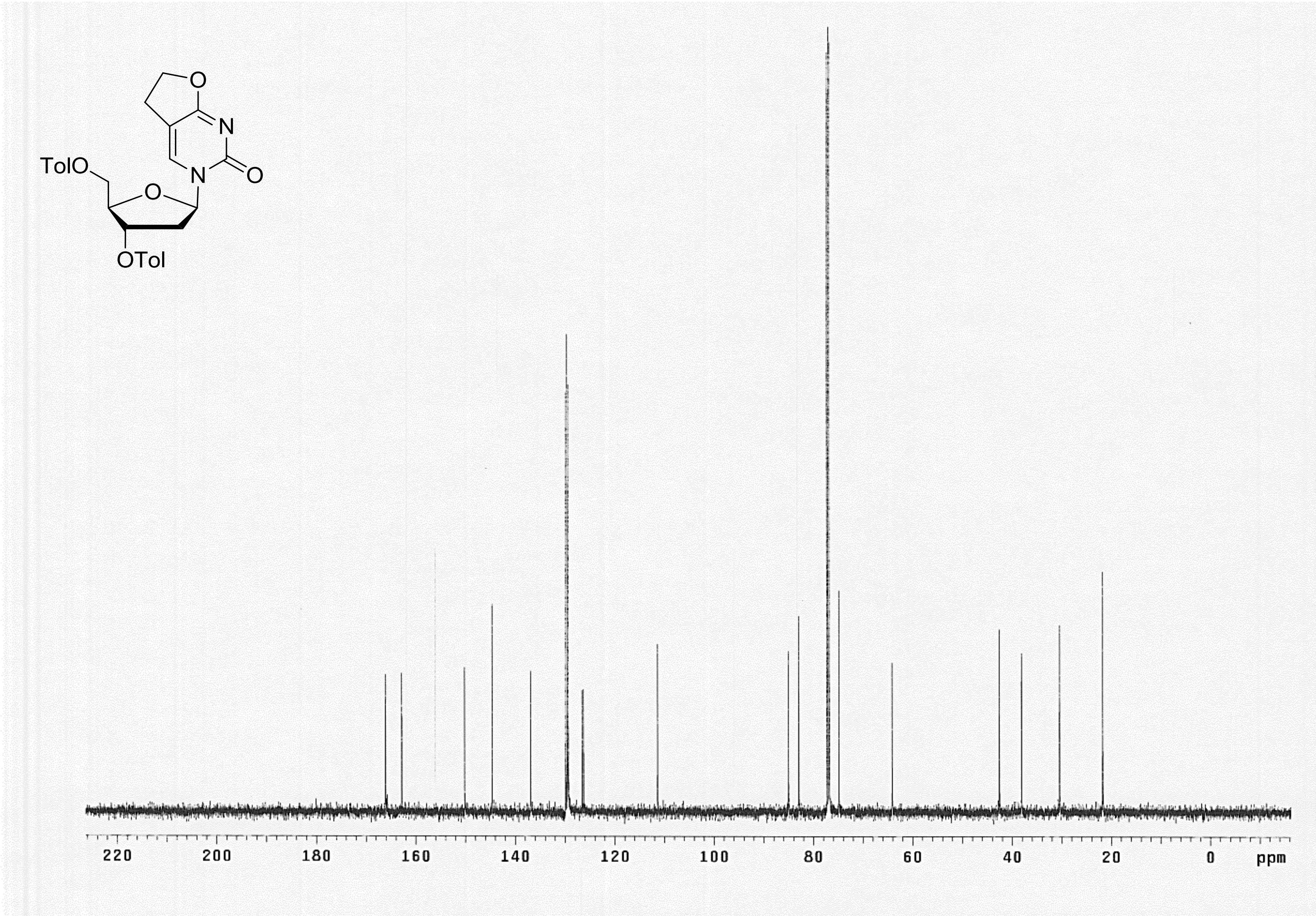

Supplementary Figure S15 - HR ESI-MS spectrum of compound (**4a**)

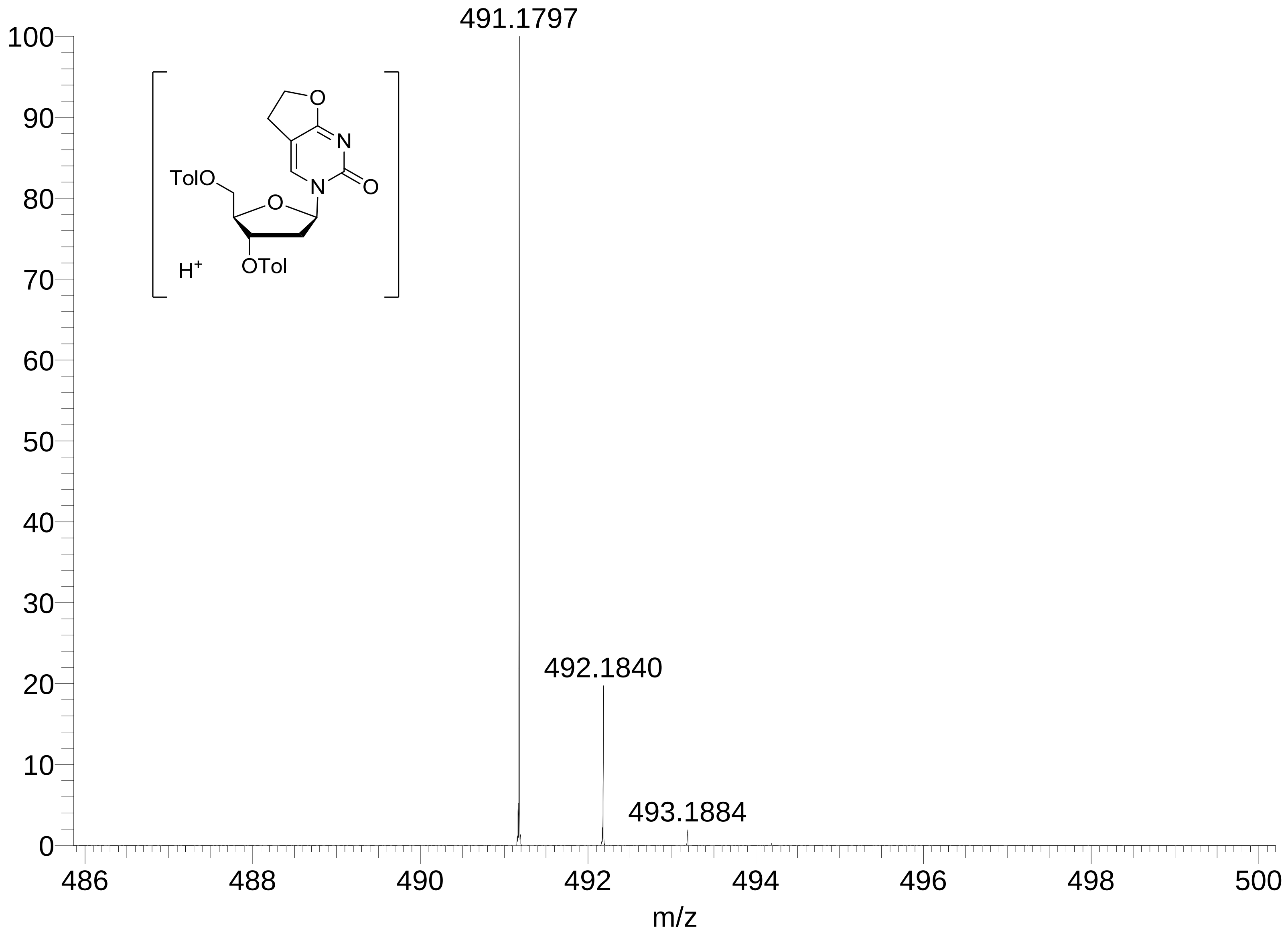

Supplementary Figure S16 - 500 MHz ^{1}H NMR spectrum of compound (**4b**) (in $CDCl_3$)

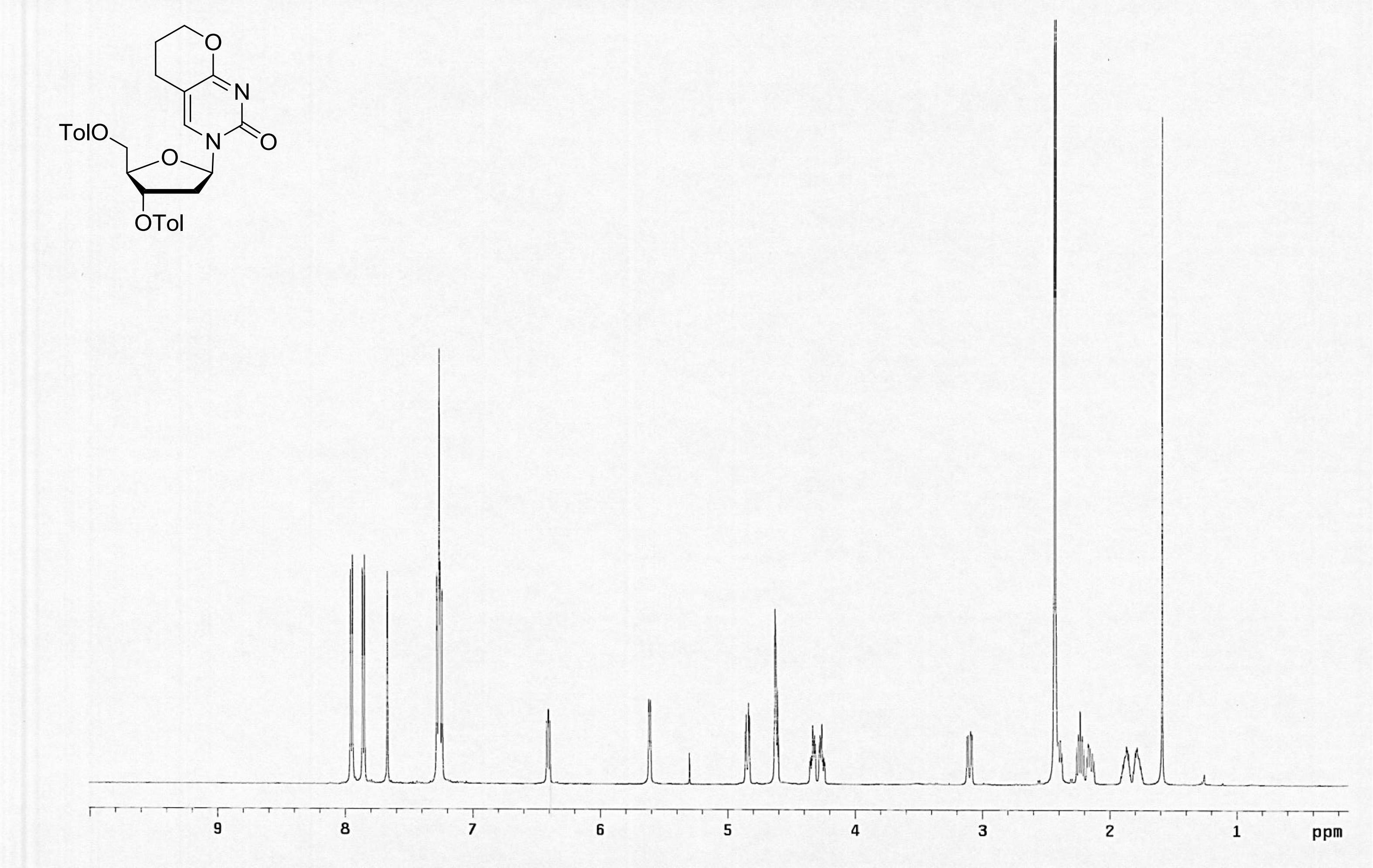

Supplementary Figure S17 - 125.7 MHz ^{13}C NMR spectrum of compound (**4b**) (in $CDCl_3$)

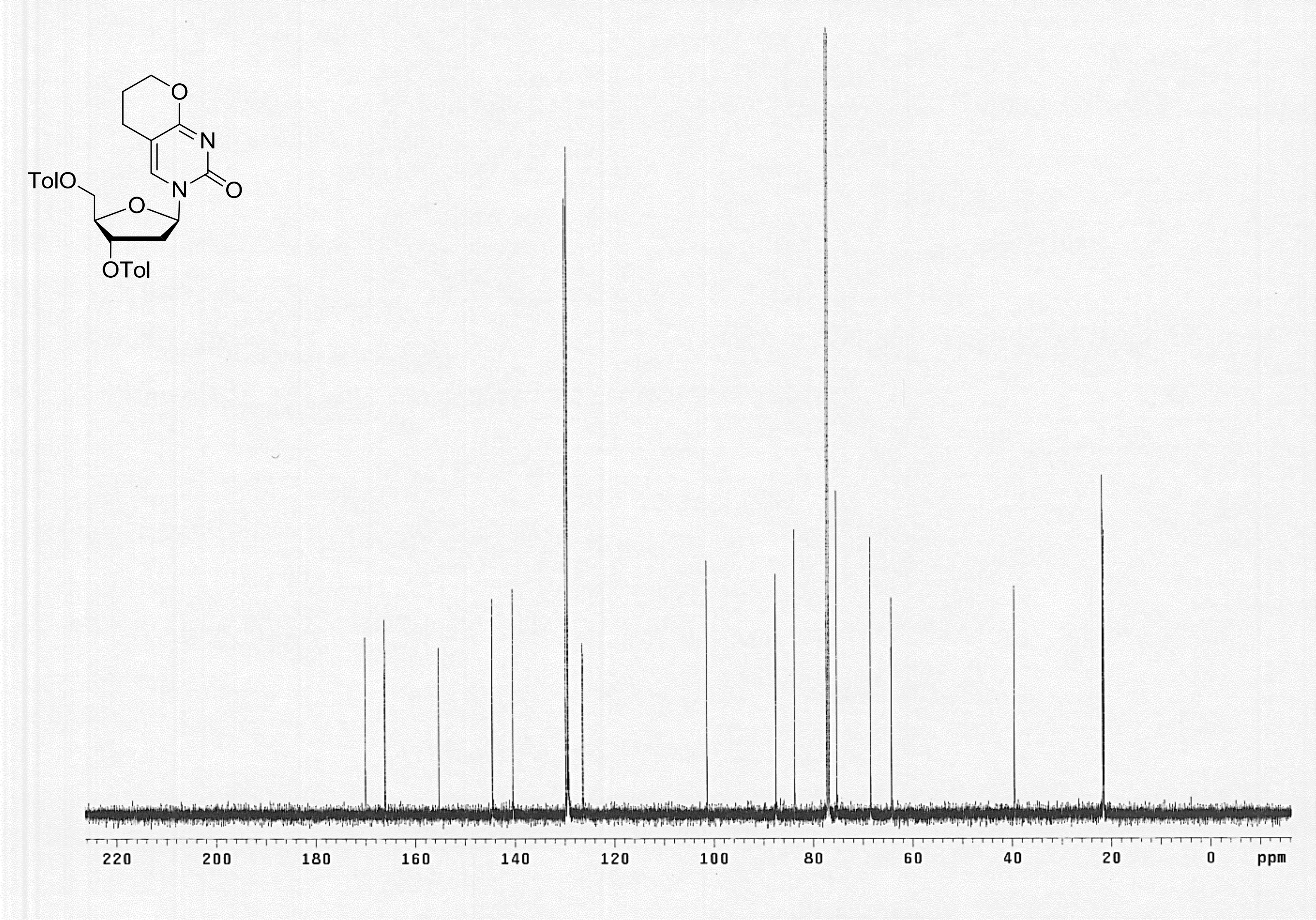

Supplementary Figure S18 - HR ESI-MS spectrum of compound (**4b**)

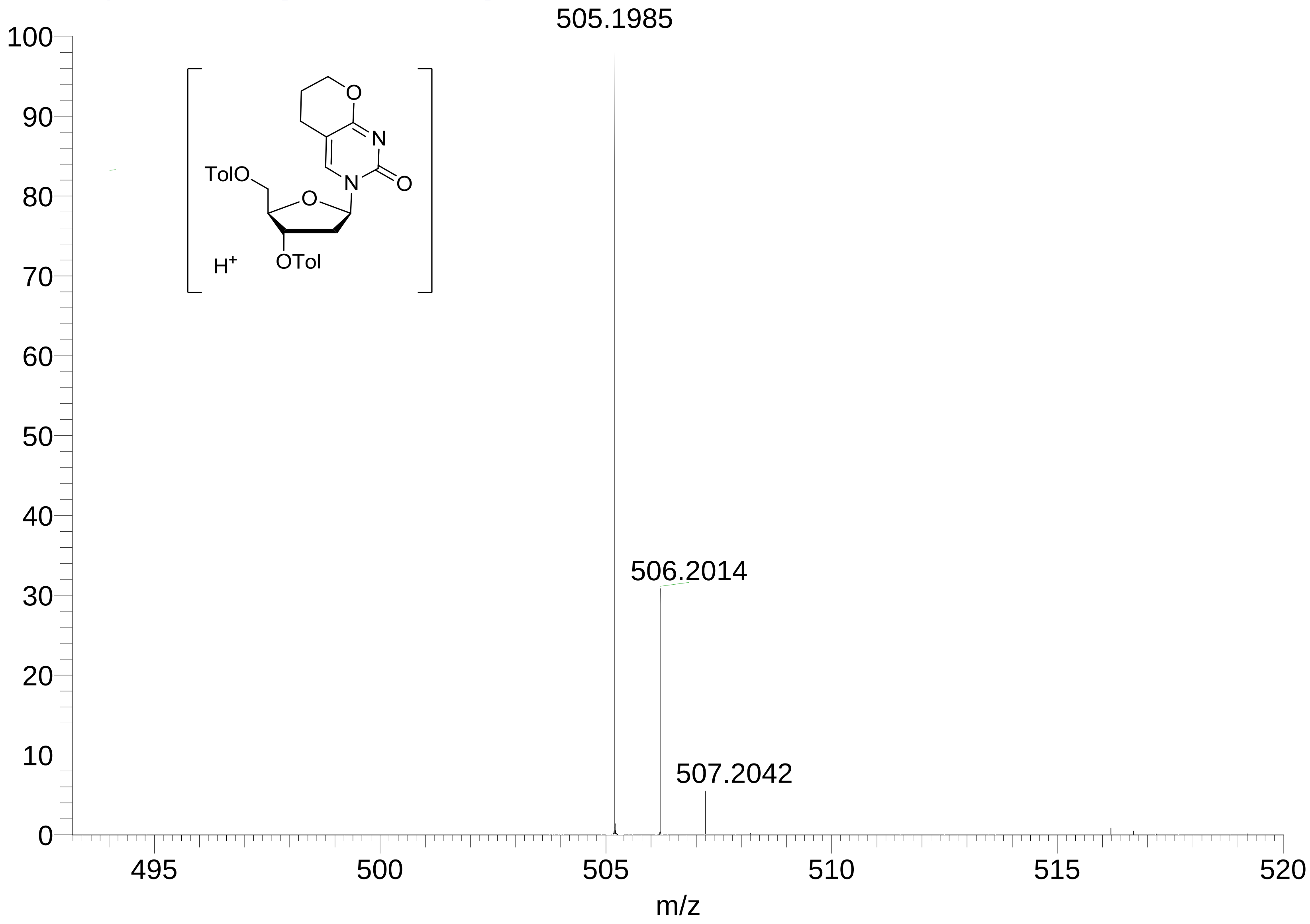

Supplementary Figure S19 - 500 MHz ^{1}H NMR spectrum of compound (**5a**) (in $CDCl_3$)

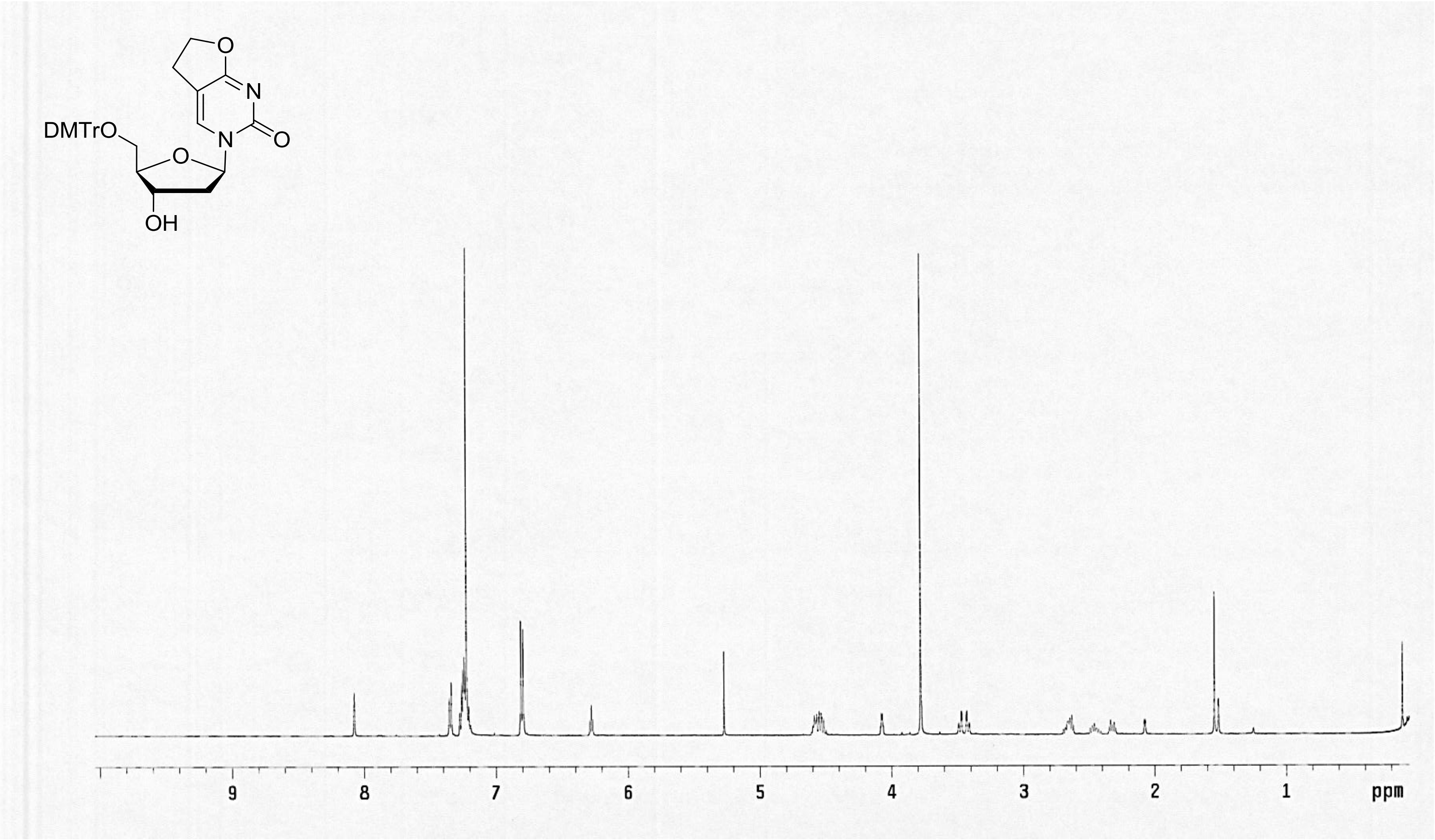

Supplementary Figure S20 - 125.7 MHz ^{13}C NMR spectrum of compound (**5a**) (in $CDCl_3$)

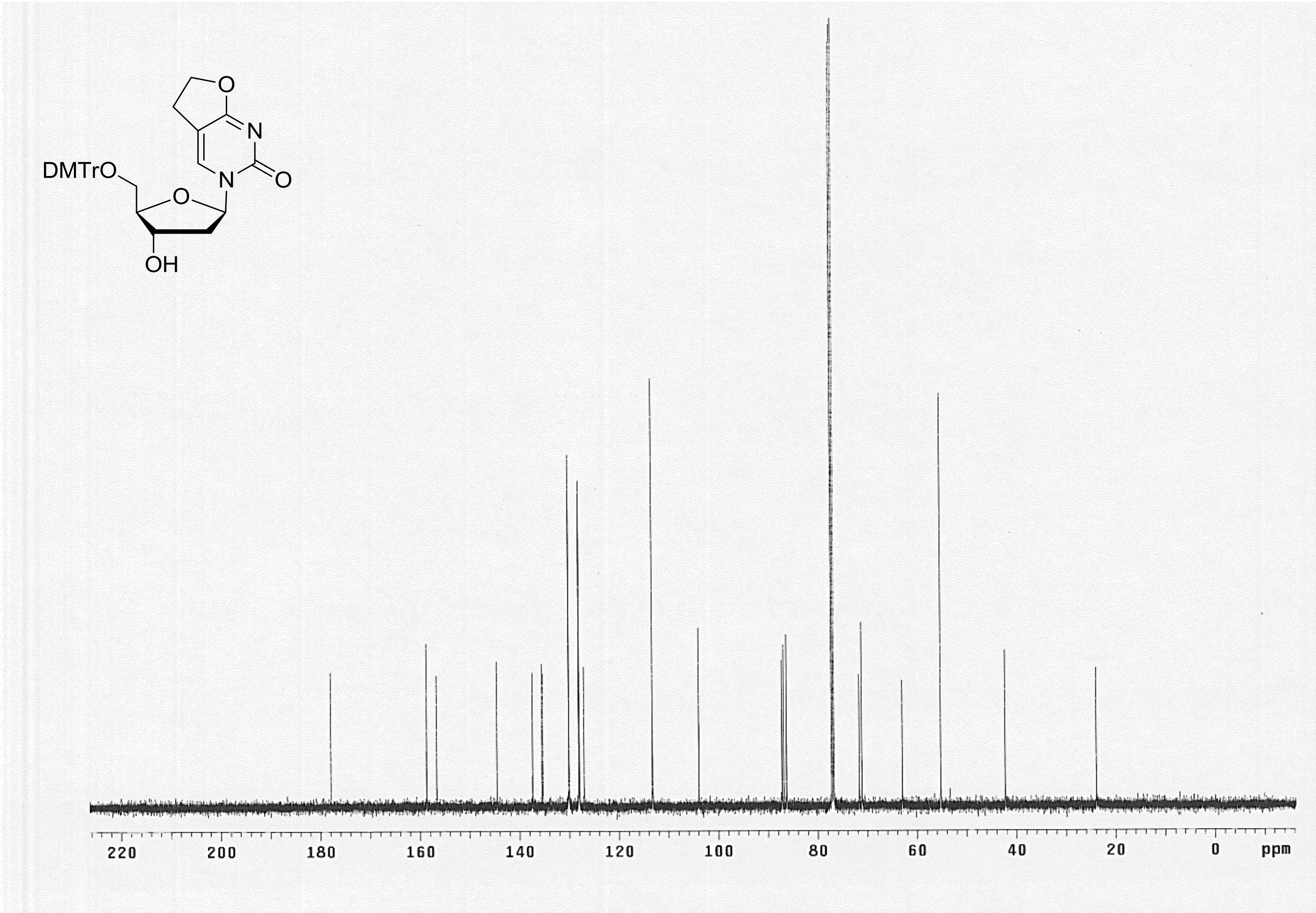

Supplementary Figure S21 - HR ESI-MS spectrum of compound (**5a**)

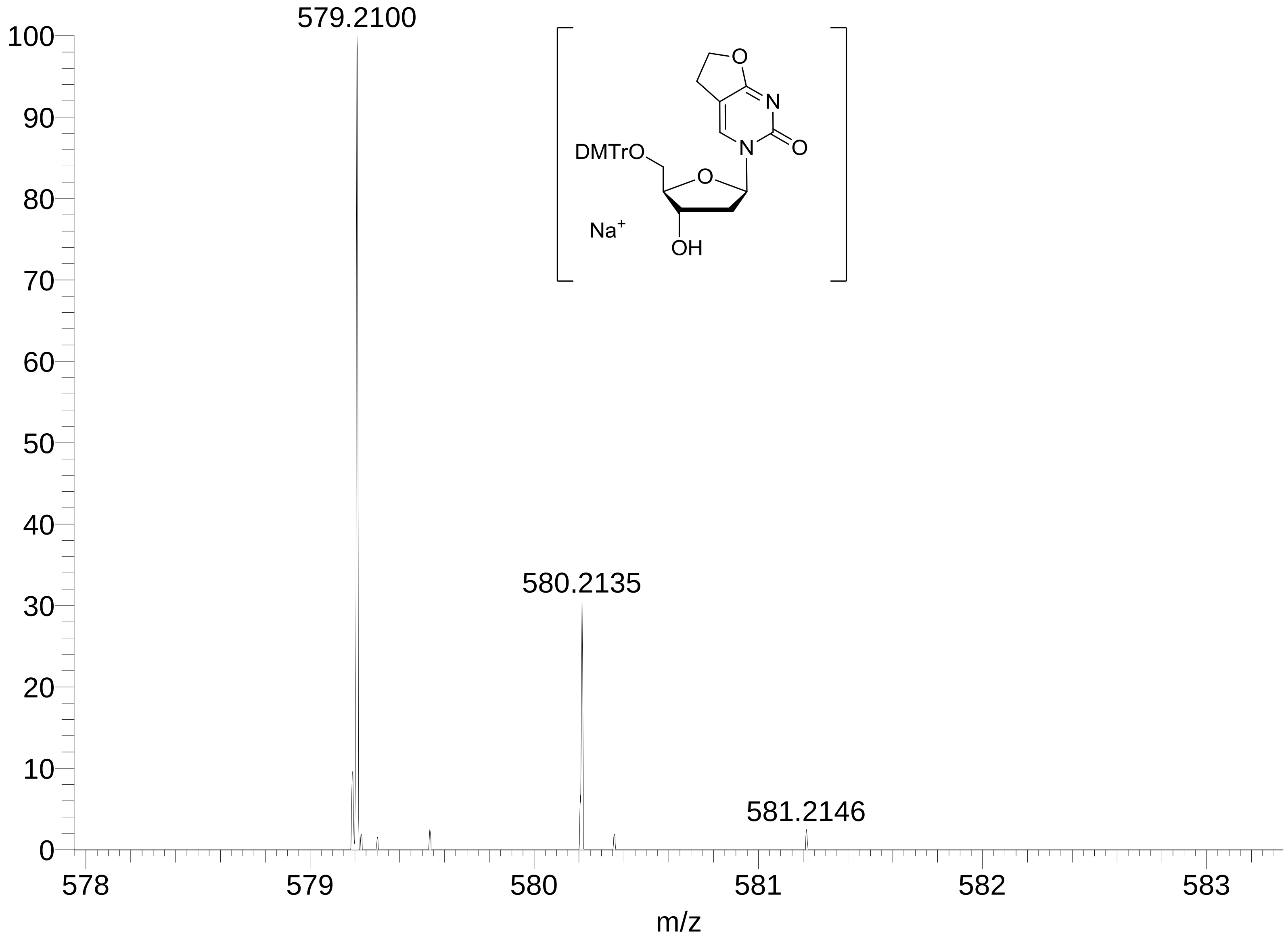

Supplementary Figure S22 - 500 MHz ^{1}H NMR spectrum of compound (**5b**) (in $CDCl_3$)

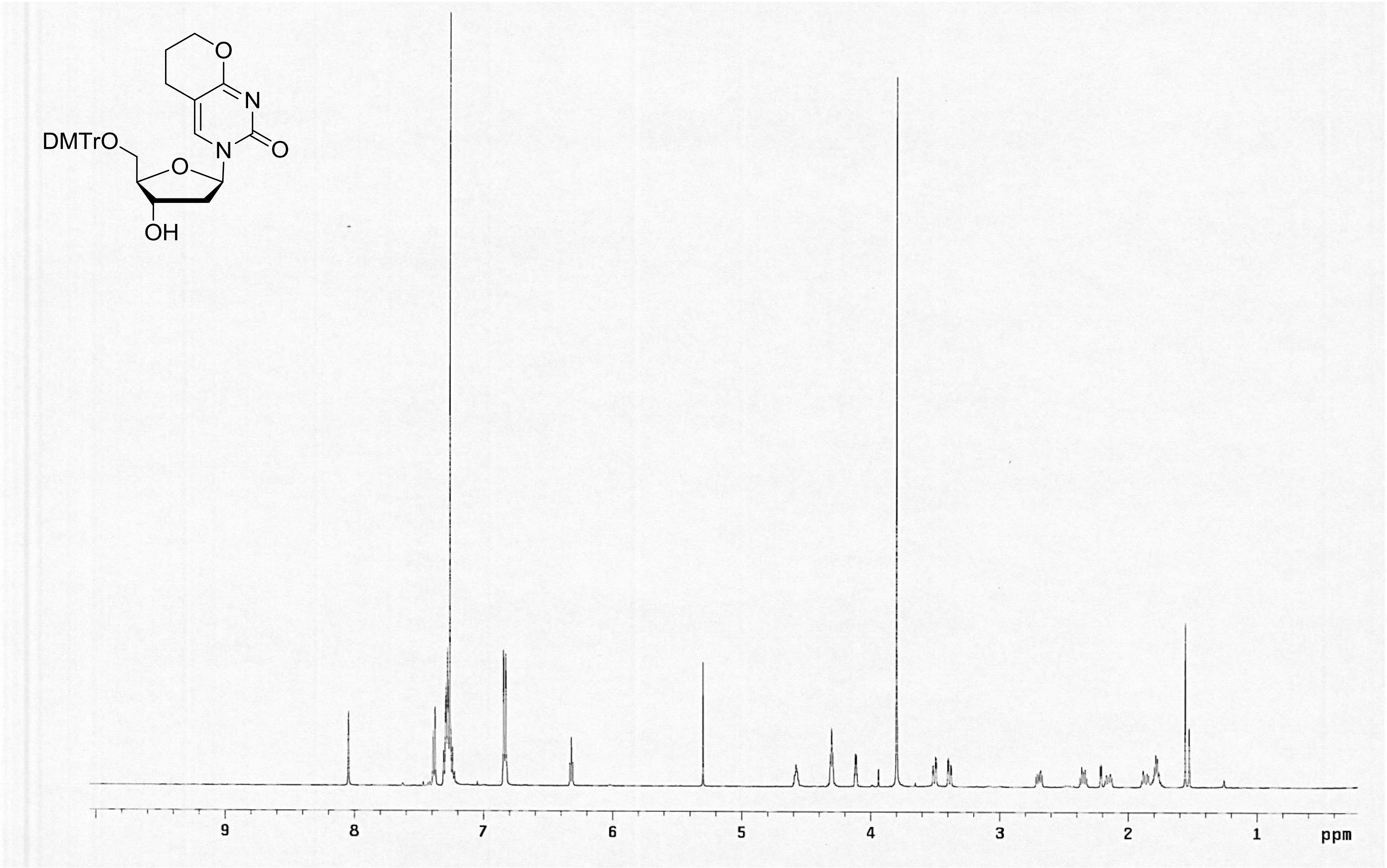

Supplementary Figure S23 - 125.7 MHz ^{13}C NMR spectrum of compound (**5b**) (in d_6-DMSO)

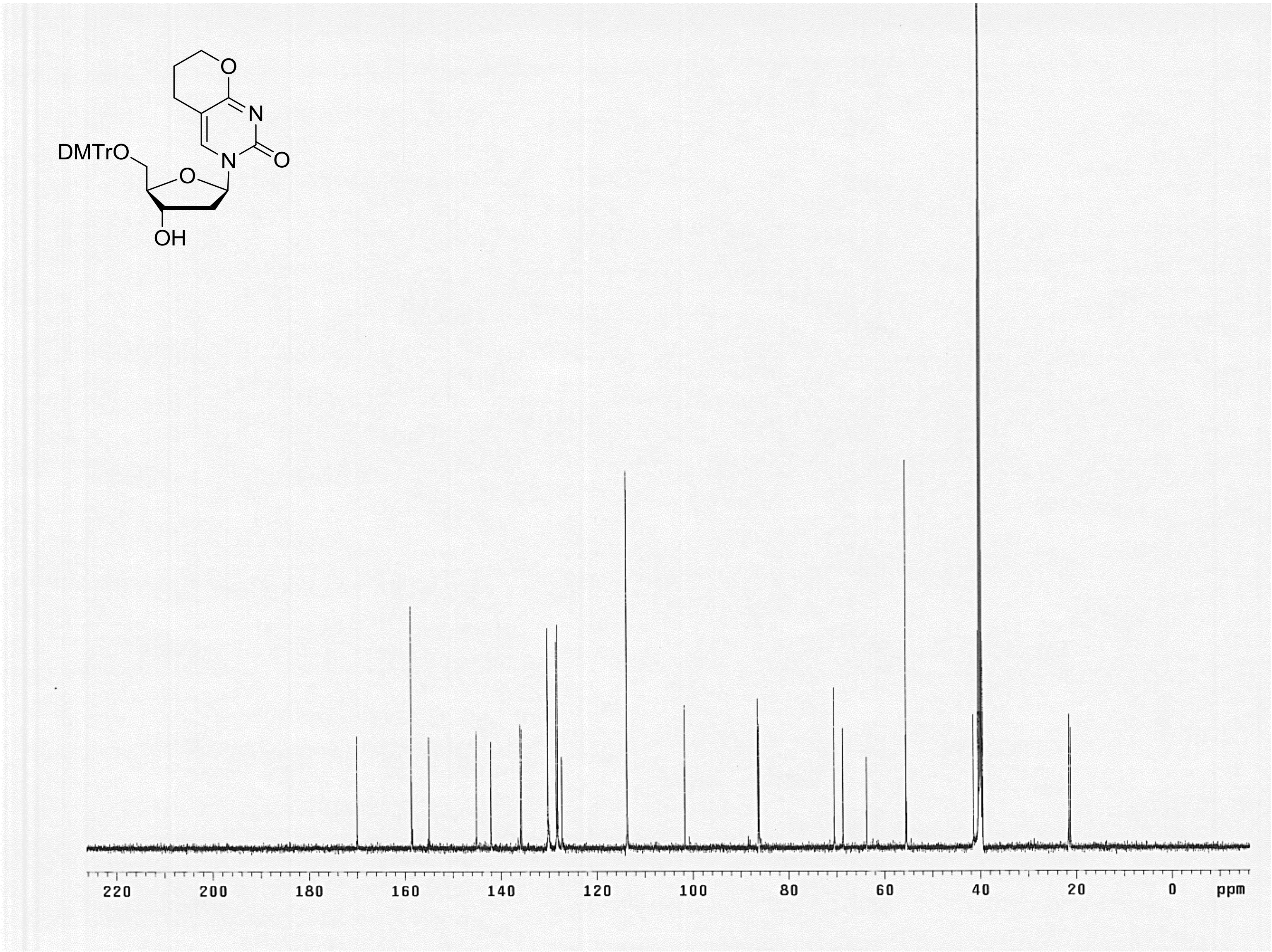

Supplementary Figure S24 - HR ESI-MS spectrum of compound (**5b**)

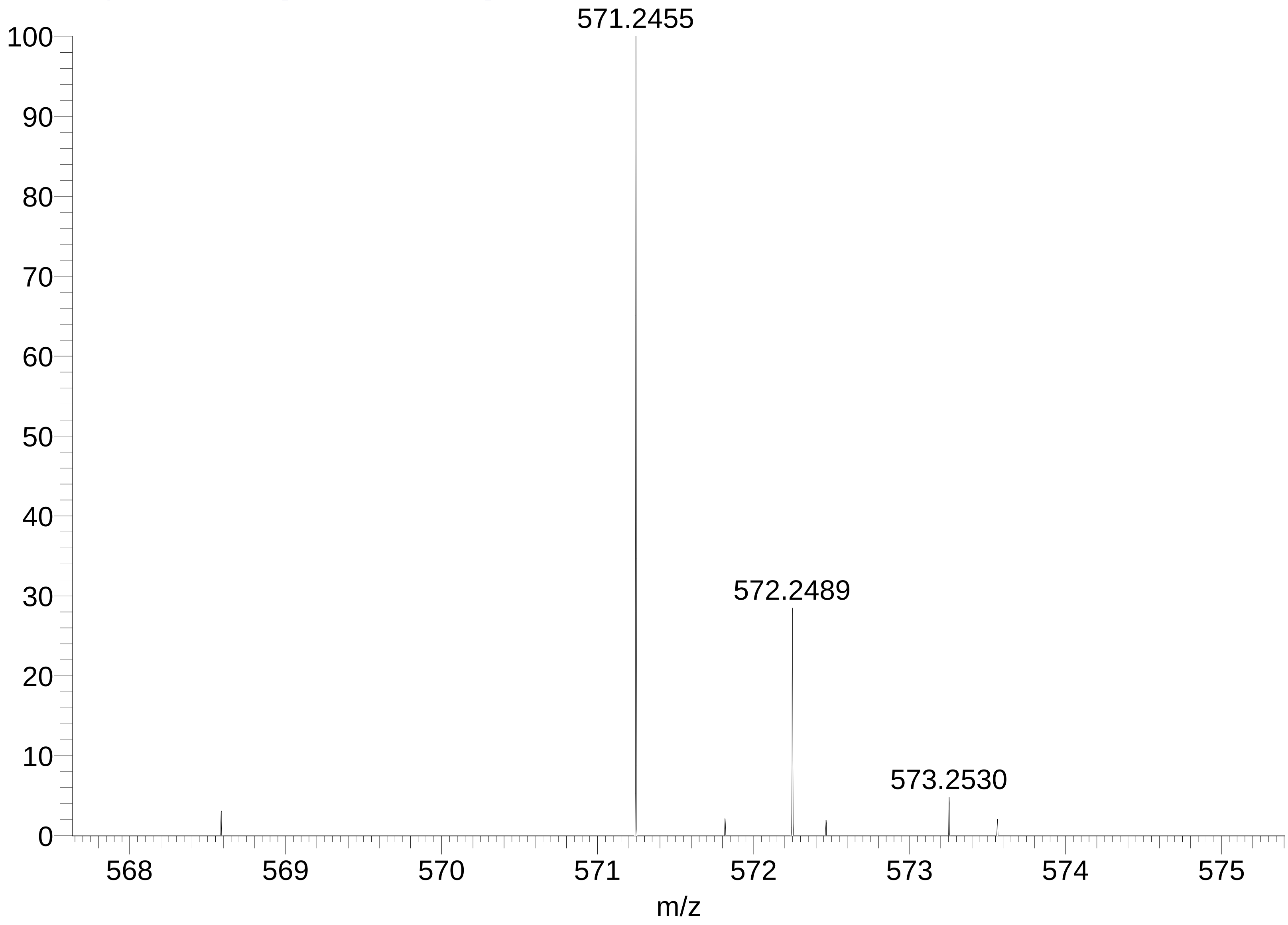

Supplementary Figure S25 - 500 MHz ^{1}H NMR spectrum of compound (**6a**) (in d_6-acetone)

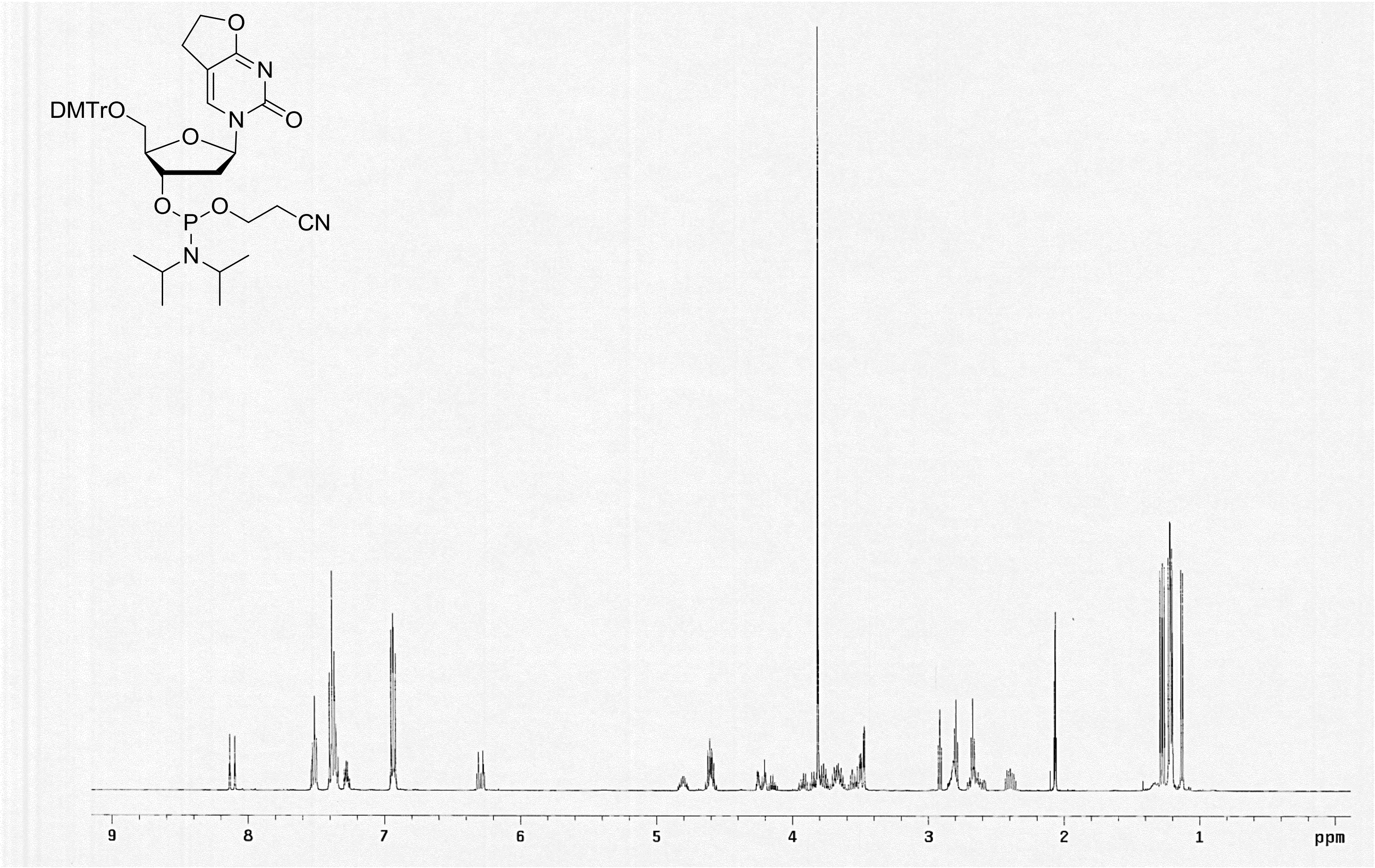

Supplementary Figure S26 - 125.7 MHz ^{13}C NMR spectrum of compound (**6a**) (in d_6-acetone)

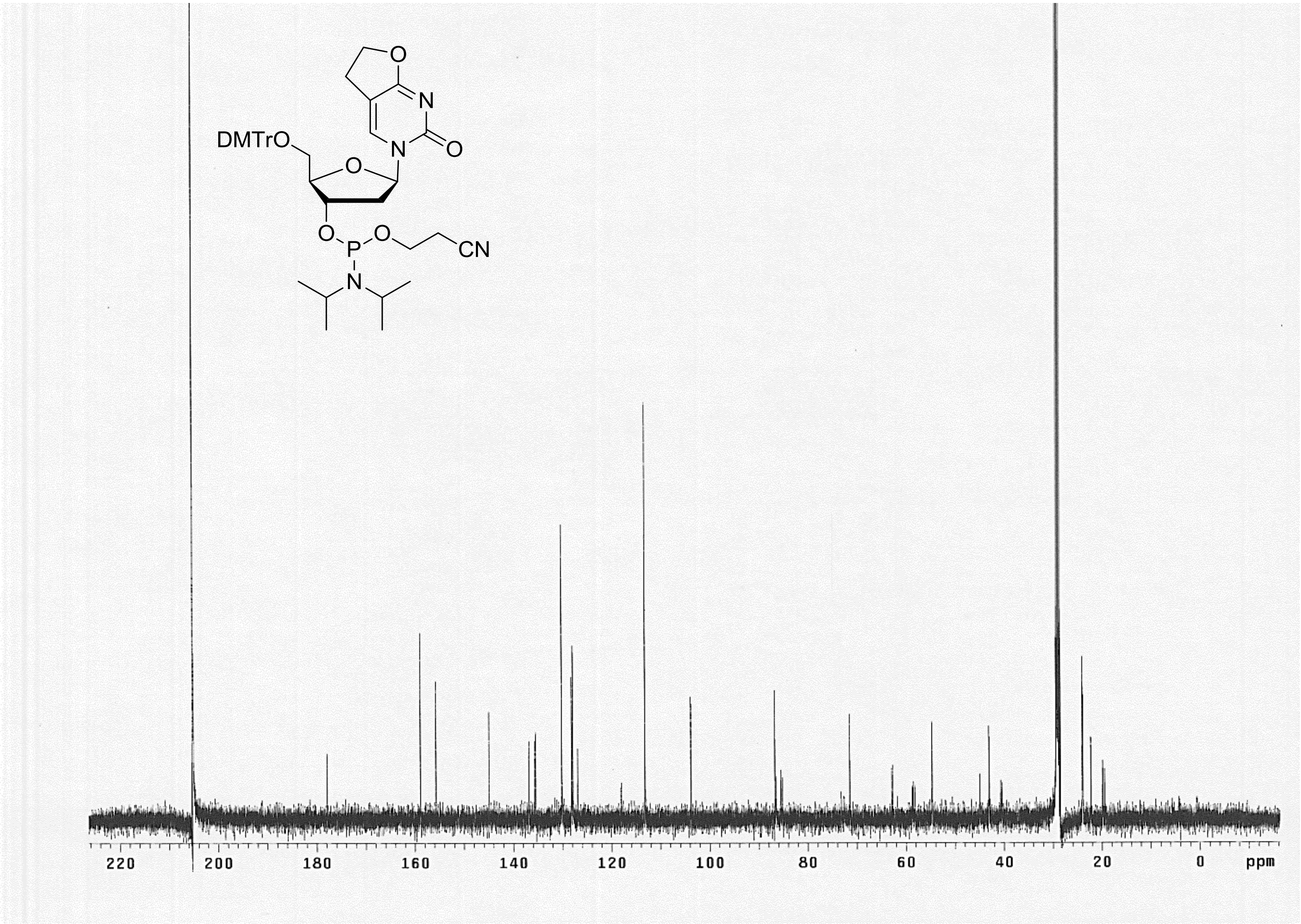

Supplementary Figure S27 - 202.3 MHz ^{31}P NMR spectrum of compound (**6a**) (in d_6-acetone)

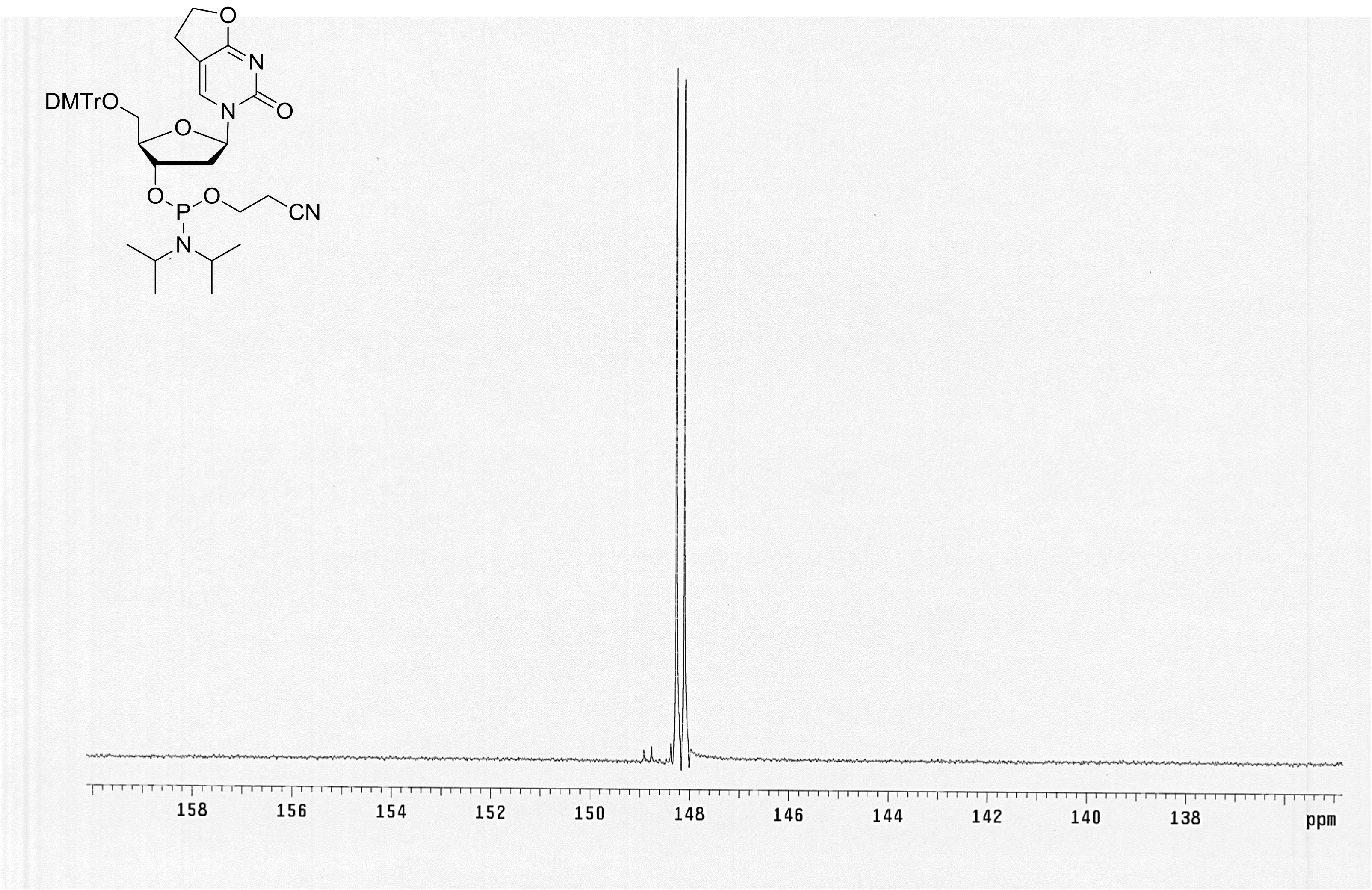

Supplementary Figure S28 - HR ESI-MS spectrum of compound (**6a**)

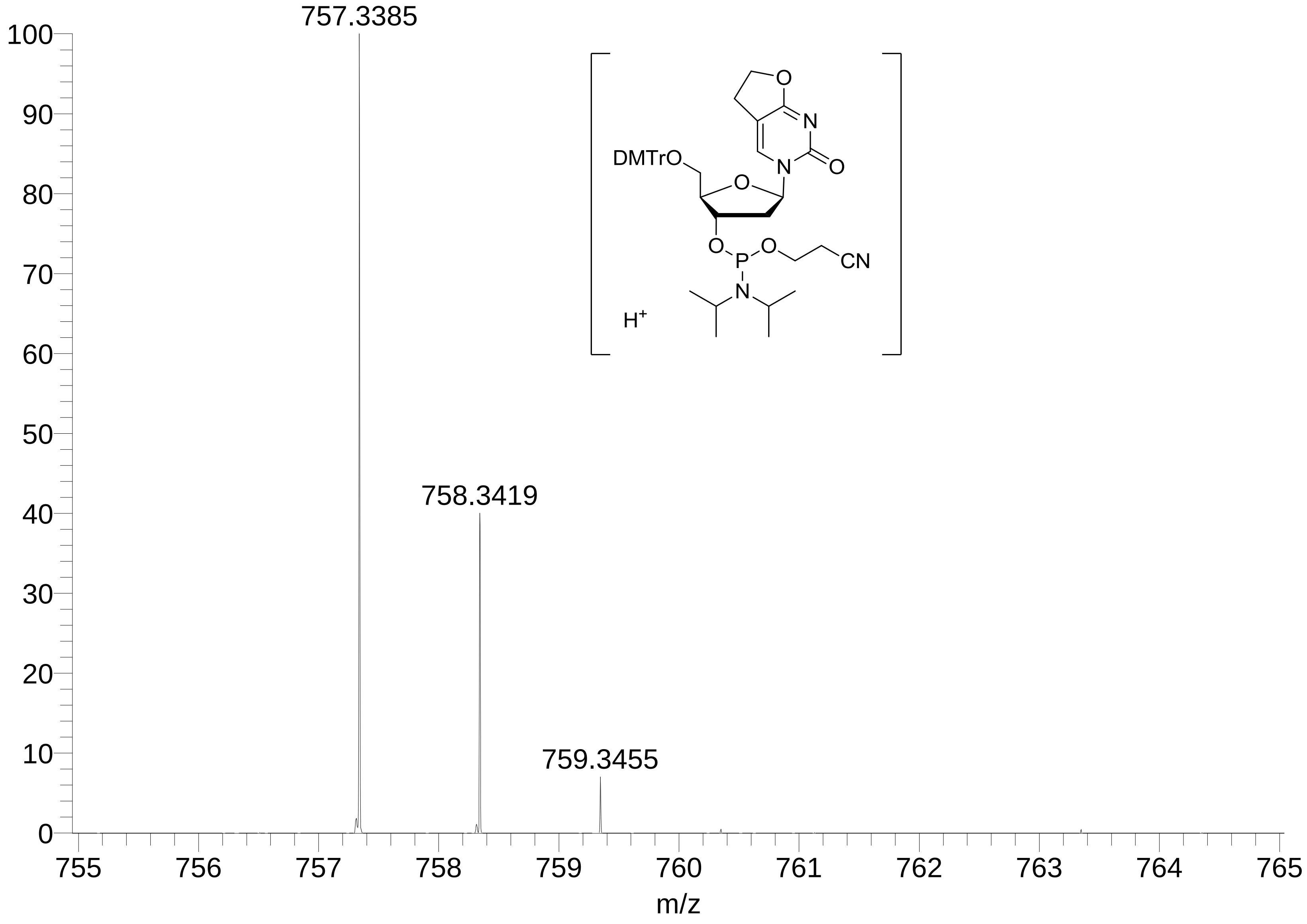

Supplementary Figure S29 - 500 MHz ^{1}H NMR spectrum of compound (**6b**) (in d_6-acetone)

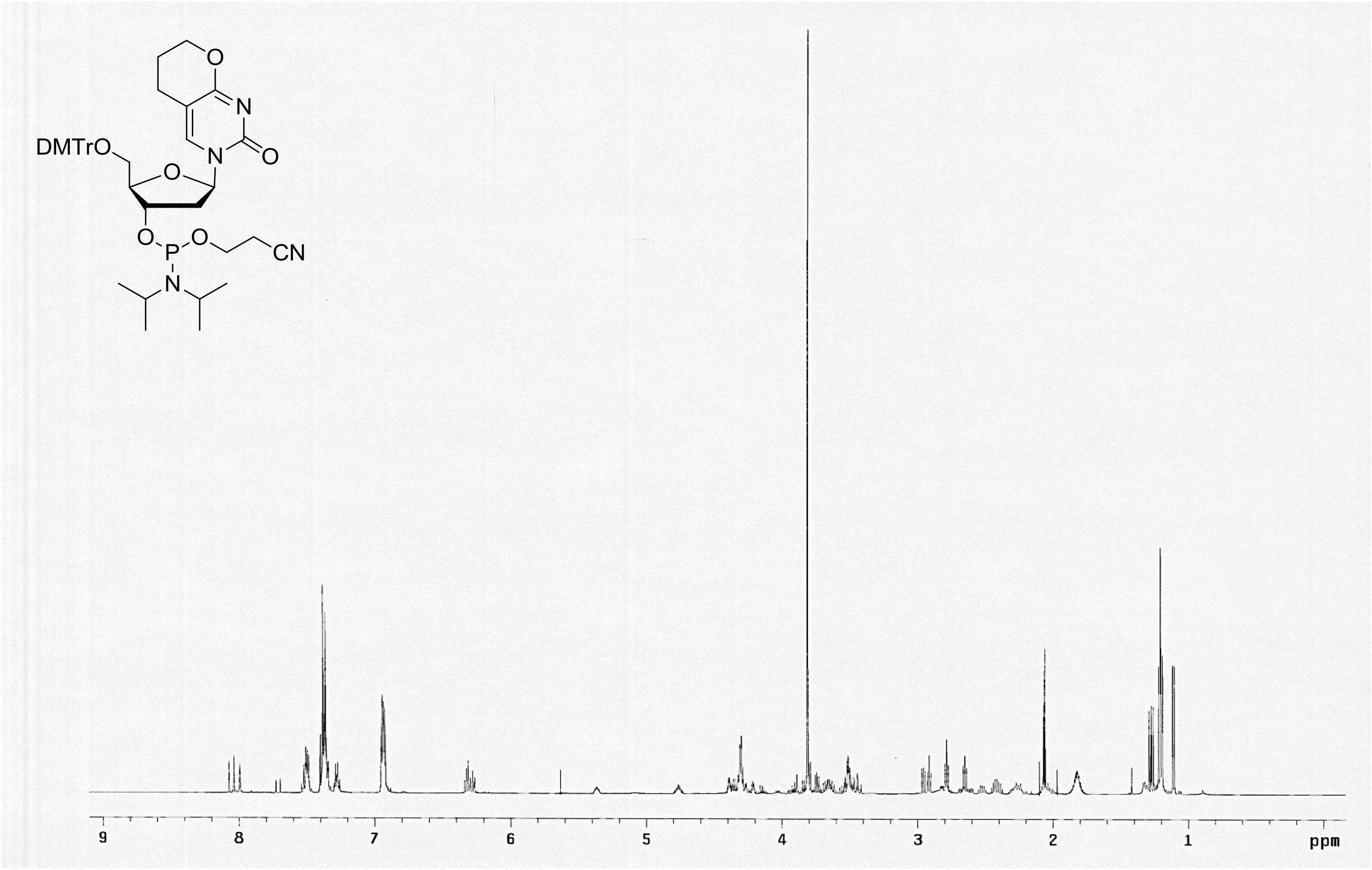

Supplementary Figure S30 - 125.7 MHz ^{13}C NMR spectrum of compound (**6b**) (in d_6-acetone)

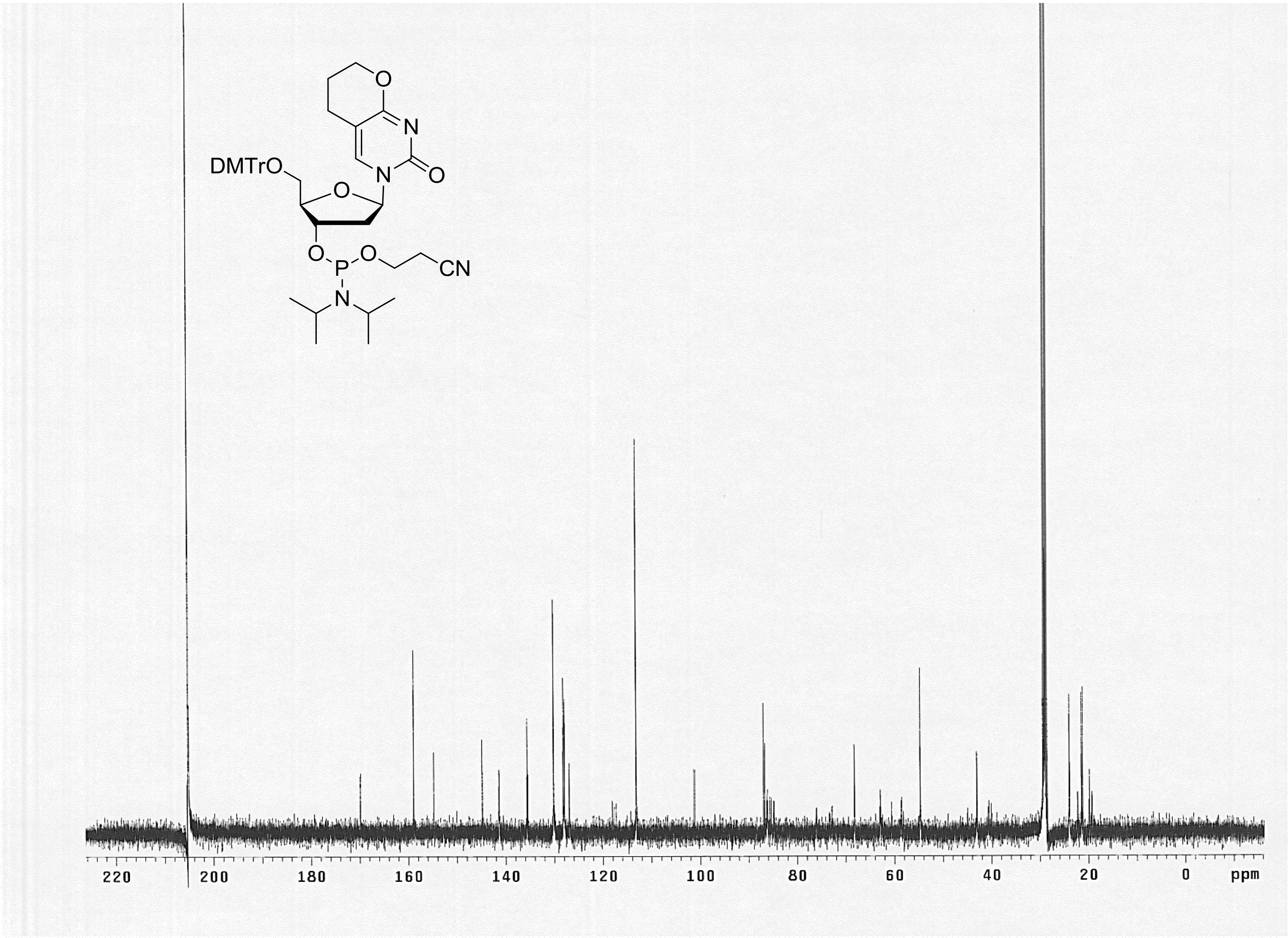

Supplementary Figure S31 - 202.3 MHz ^{31}P NMR spectrum of compound (**6b**) (in d_6-acetone)

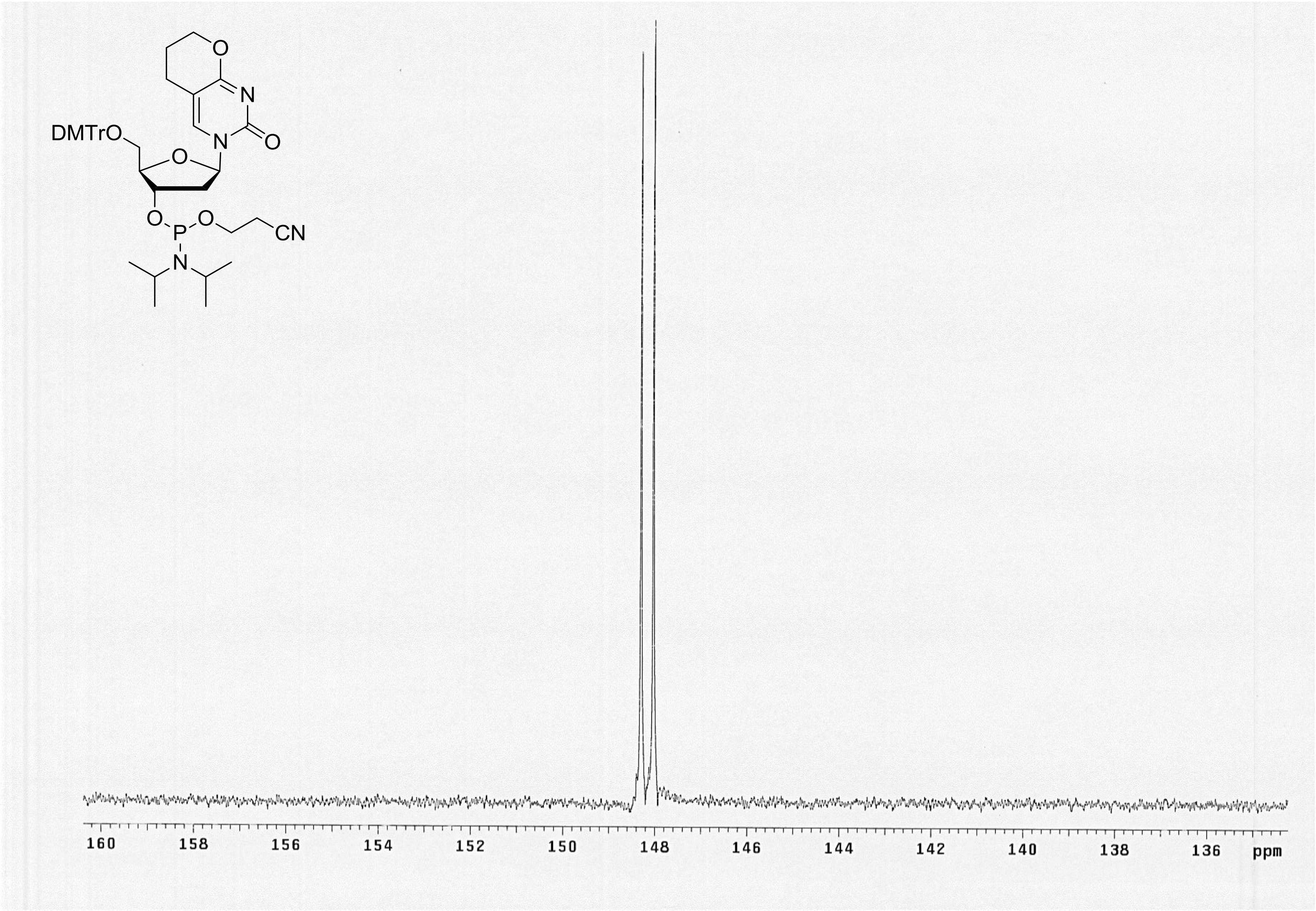

Supplementary Figure S32 - HR ESI-MS spectrum of compound (**6b**)

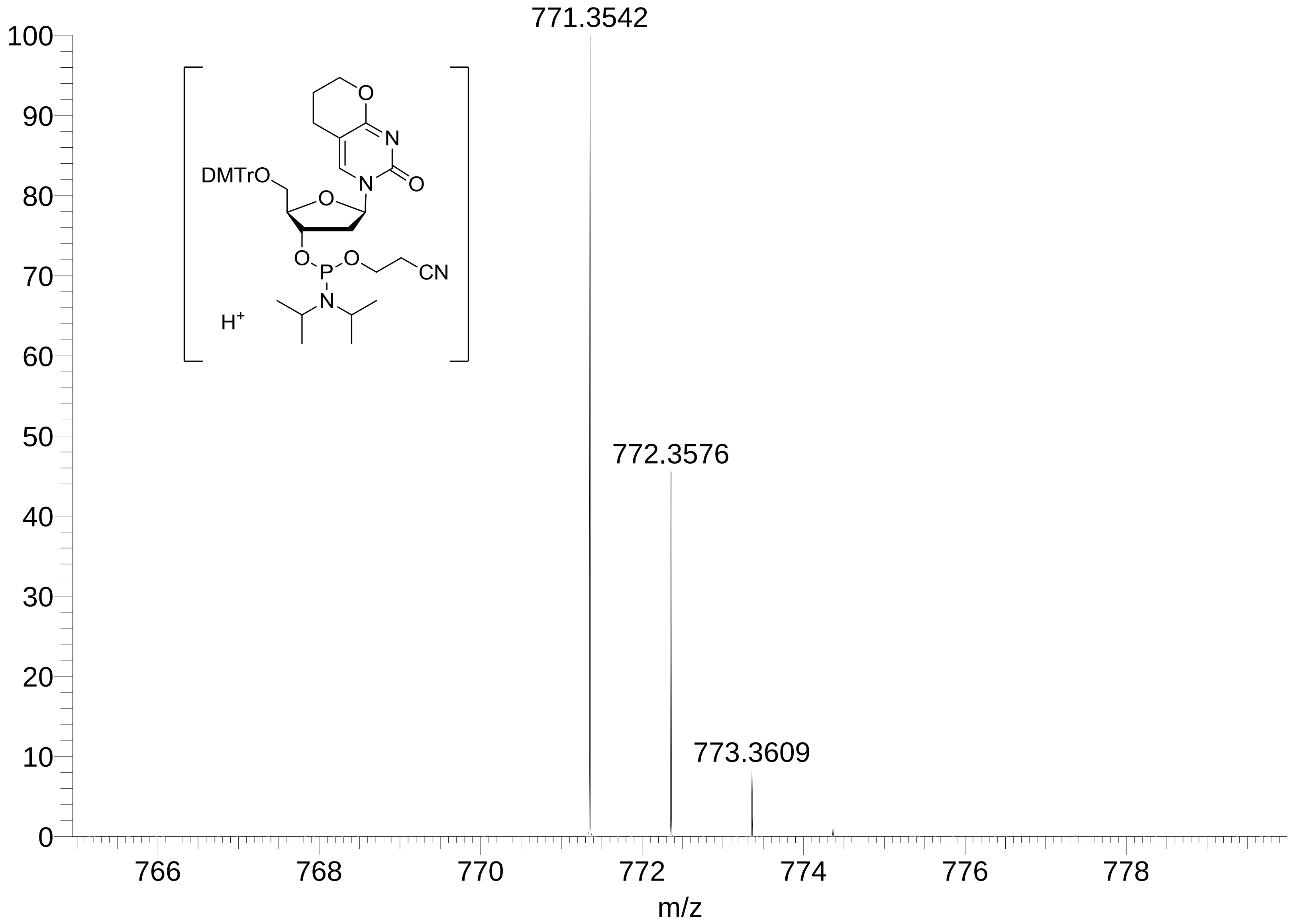

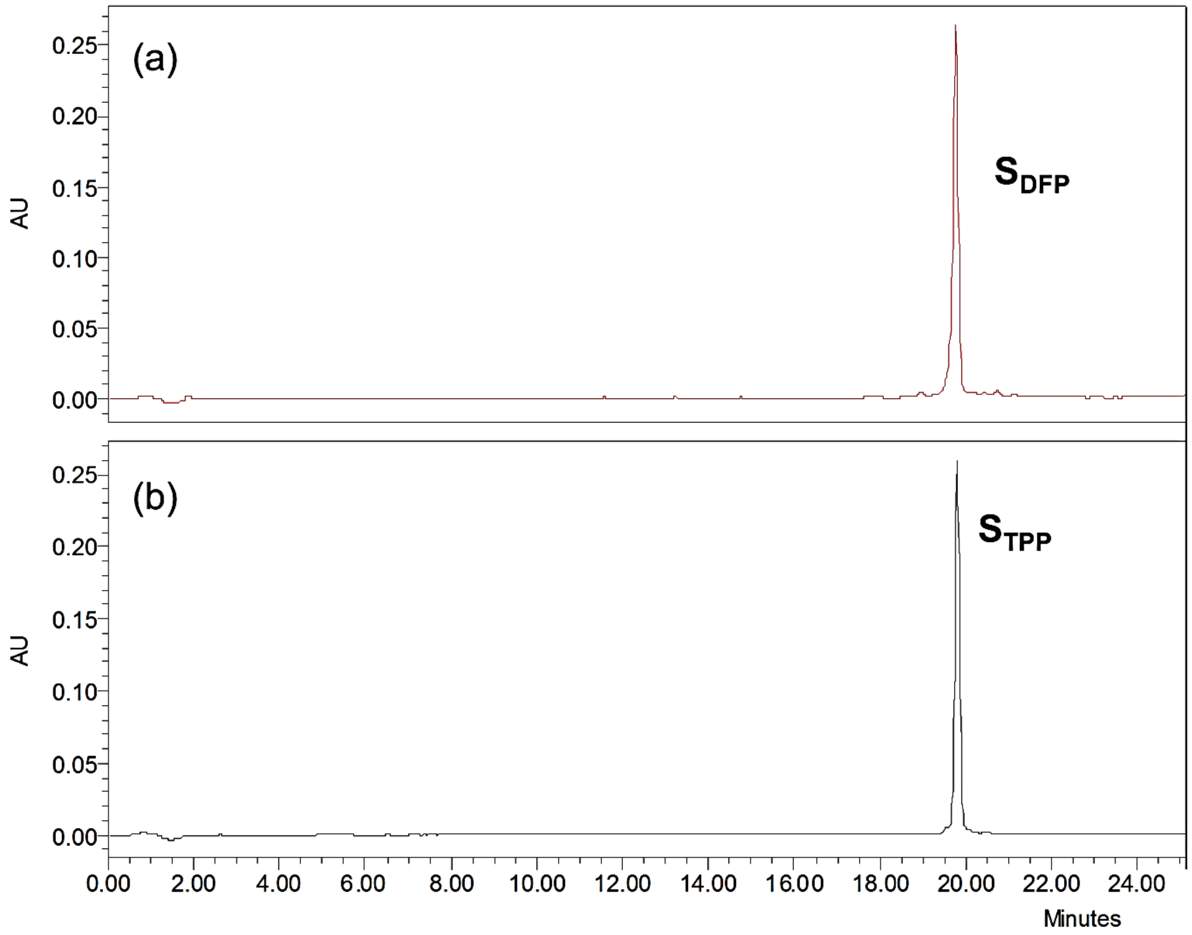

Supplementary Figure S33 - SAX-HPLC profile of purified (**a**) **S$_{DFP}$** and (**b**) **S$_{TPP}$.** The column was eluted using a linear gradient of 0-52% buffer B over 24 min (buffer A: 100 mM Tris HCl, pH 7.5, 10% acetonitrile and buffer B: 100mMTris HCl, pH 7.5, 10% acetonitrile, 1 M NaCl) at 55°C.

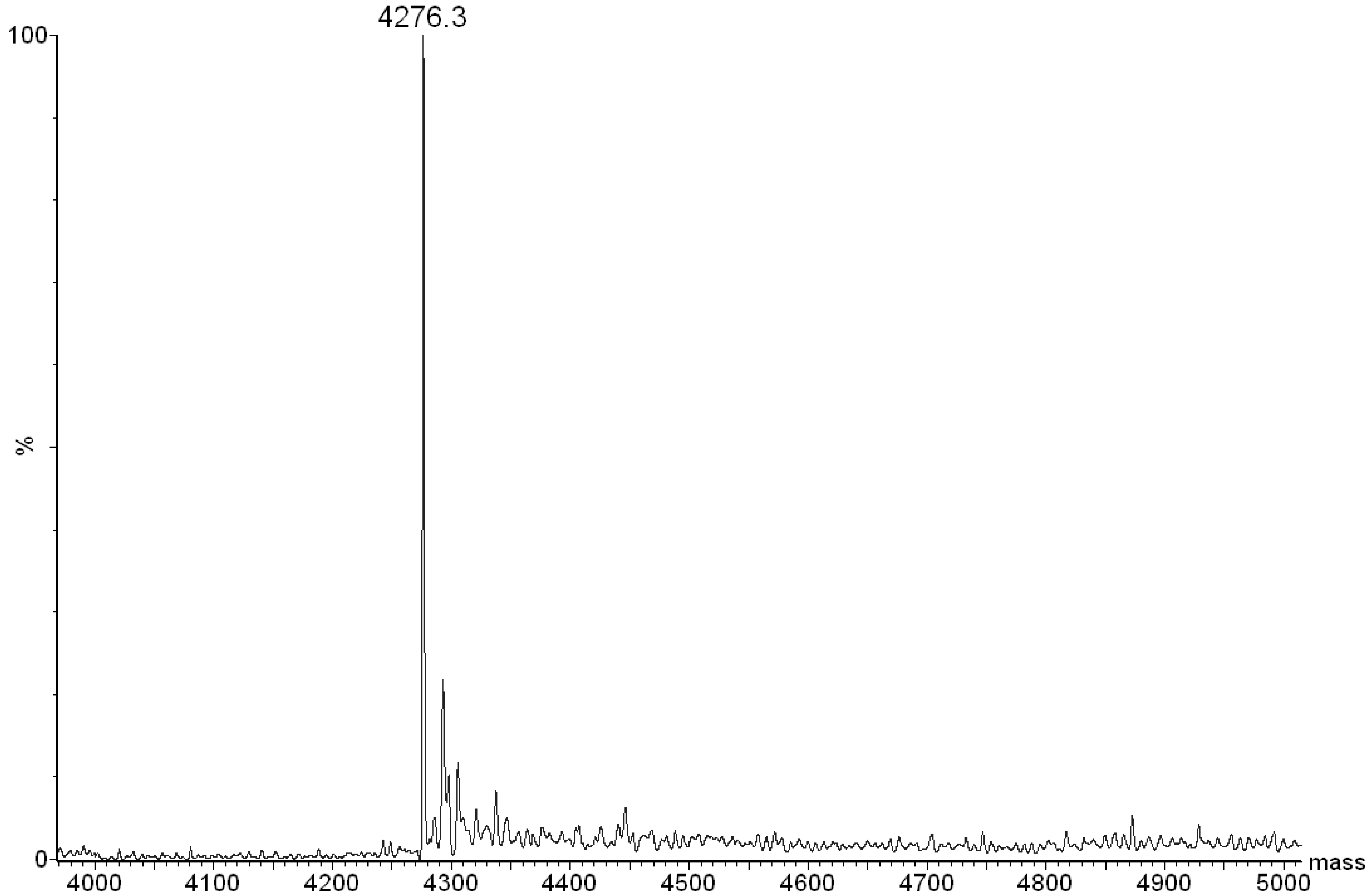

Supplementary Figure S34 - ESI MS of oligonucleotide $\mathbf{S_{DFP}}$

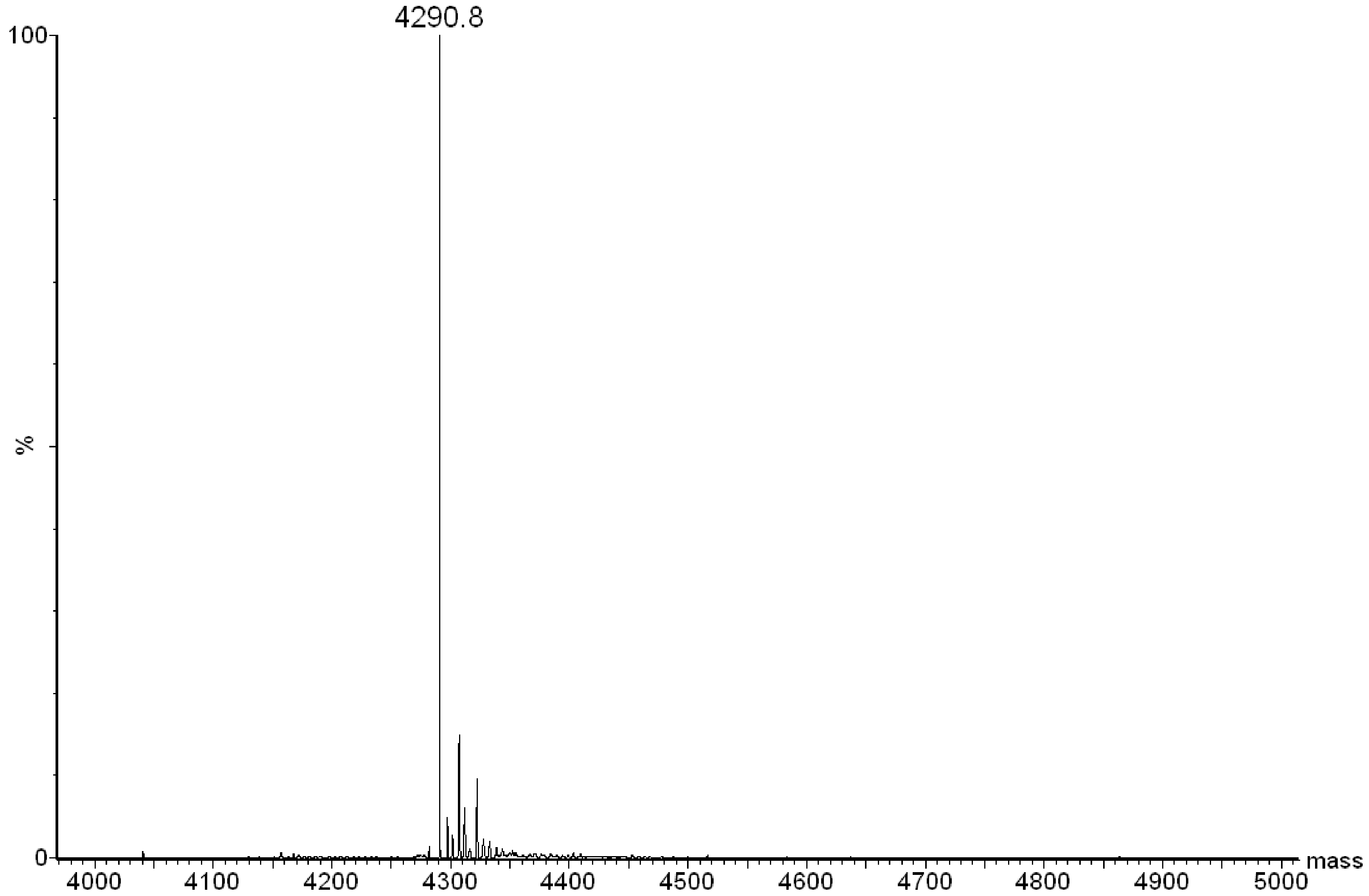

Supplementary Figure S35 - ESI MS of oligonucleotide $\mathbf{S_{TPP}}$

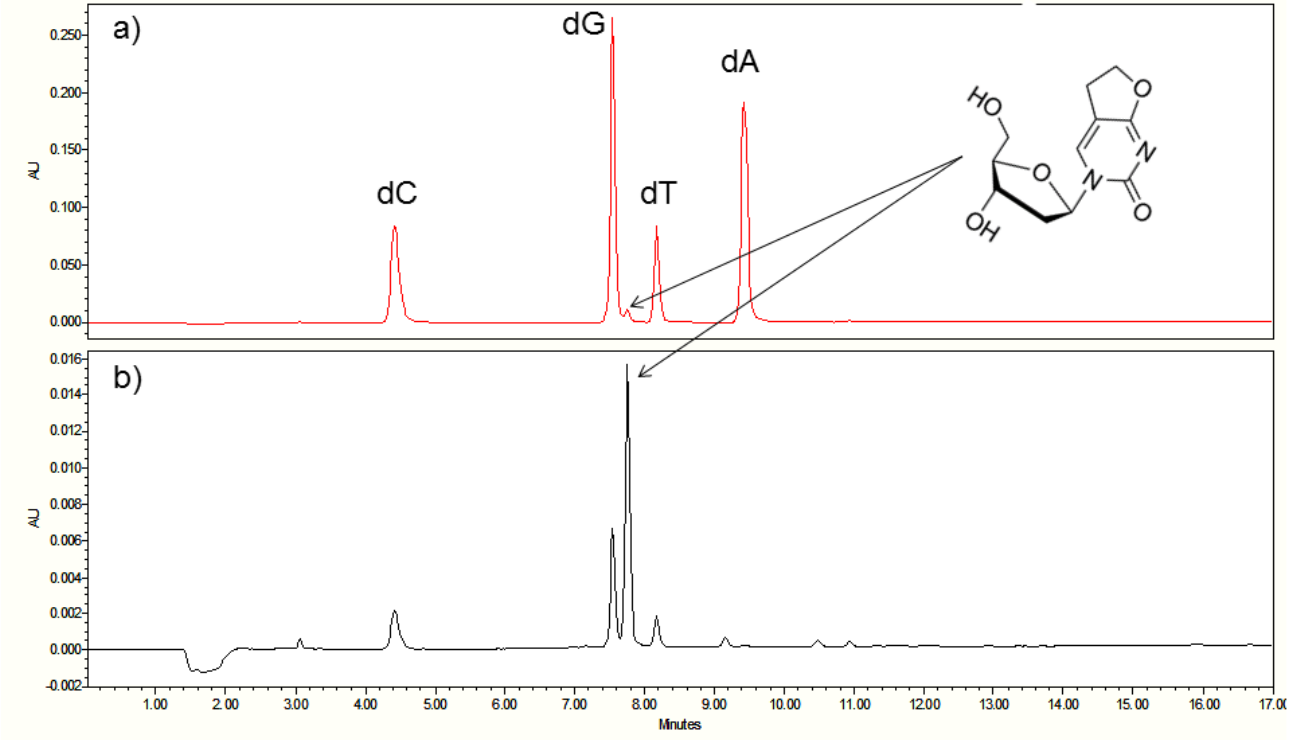

Supplementary Figure S36 - C-18 HPLC profile of digested oligomer $\mathbf{S_{DFP}}$ at (a) 260 nm and (b) 300 nm. The column was eluted with a linear gradient of 0-70% buffer B over 30 min was used to elute desired analytes (buffer A: 50 mM sodium phosphate, pH 5.8, 2% acetonitrile and buffer B: 50 mM sodium phosphate, pH 5.8, 50% acetonitrile).

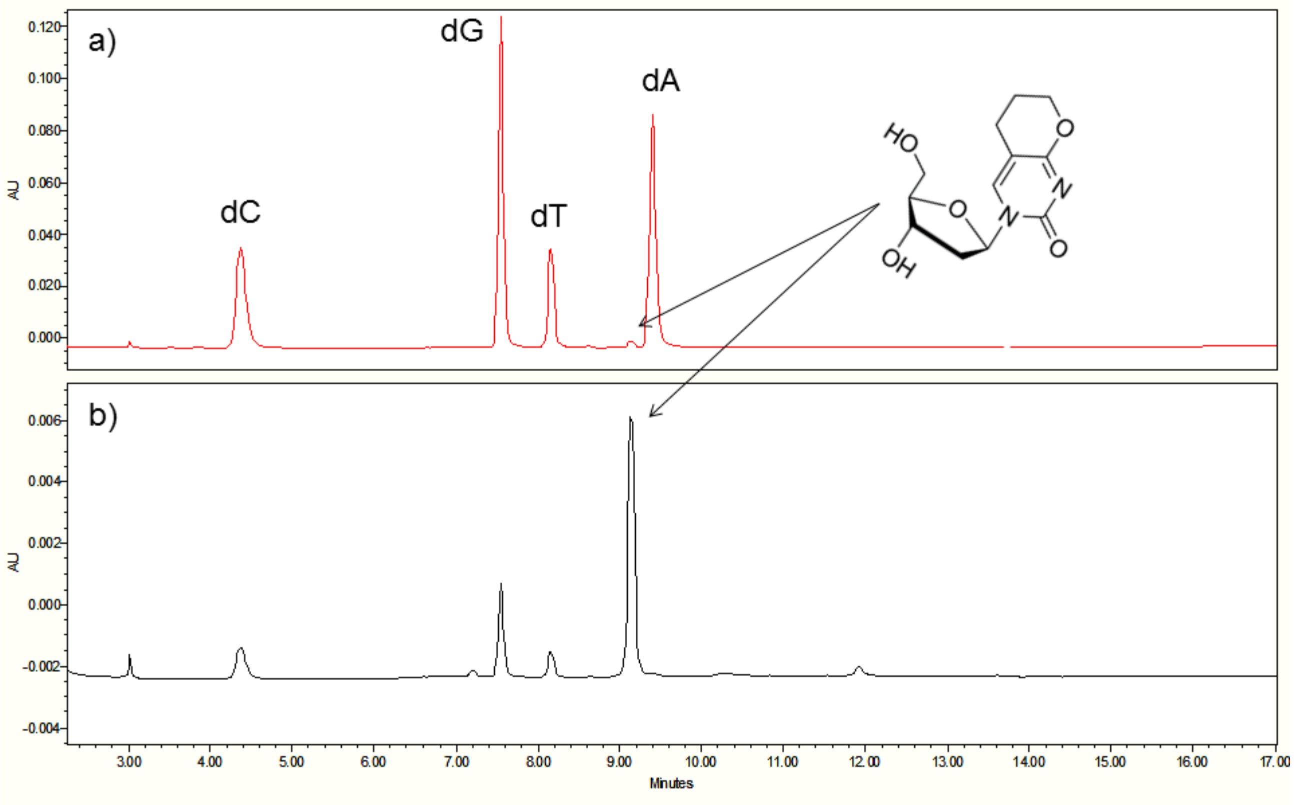

Supplementary Figure S37 - C-18 HPLC profile of digested oligomer **S$_{TPP}$** at (a) 260 nm and (b) 300 nm. The column was eluted with a linear gradient of 0-70% buffer B over 30 min was used to elute desired analytes (buffer A: 50 mM sodium phosphate, pH 5.8, 2% acetonitrile and buffer B: 50 mM sodium phosphate, pH 5.8, 50% acetonitrile).

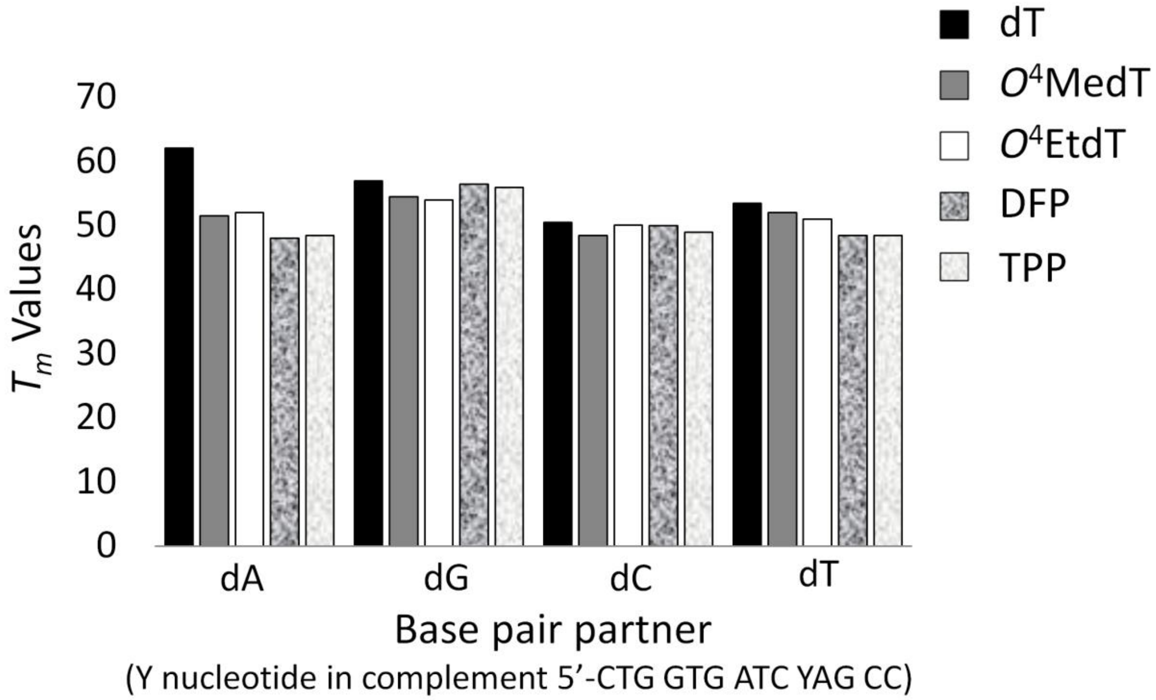

Supplementary Figure S38 - T_m values (°C) of duplexes containing dT, *O*4MedT, *O*4EtdT, DFP or TPP across different base pairing partners. The sequence of the DNA duplex was 5´GGCTXGATCACCAG 3´/5´ CTGGTGATCYAGCC 3´ where X is dT, *O*4MedT, *O*4EtdT, DFP or TPP and Y is dA, dG, dC or dT. The solutions contain a total strand concentration of 3.8 μM for the duplexes in a buffer consisting of 90 mM sodium chloride, pH 7.0, 10 mM sodium phosphate, and 1 mM EDTA buffer. Data was acquired at 0.5 °C/min from 90 °C to 15 °C monitoring UV absorption at 260 nm. Samples were held at 15 °C for 2 min and re-heated at 0.5 °C/min to 90 °C showing reversibility (data not shown).

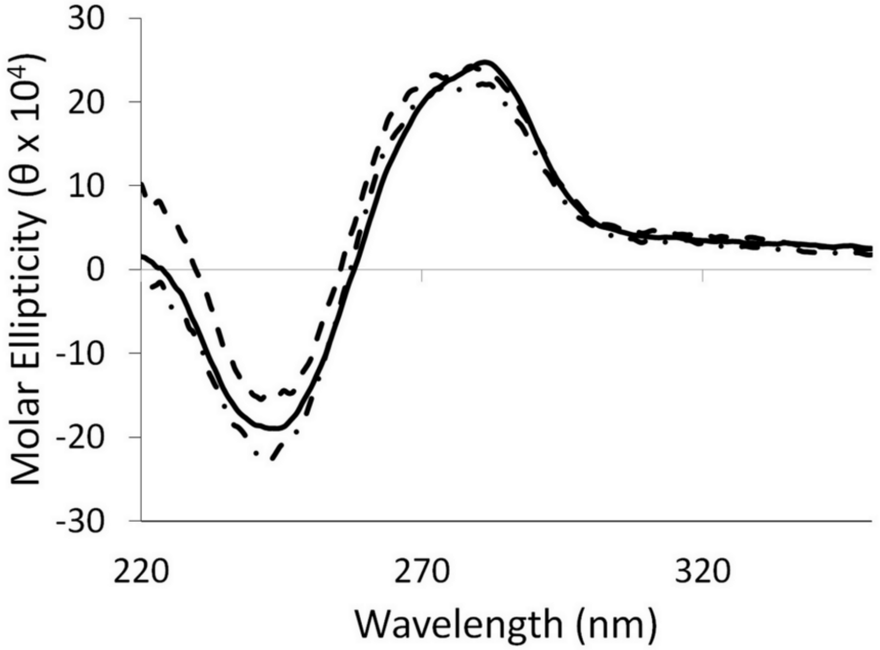

Supplementary Figure S39 - Far-UV circular dichroism spectra of DNA sequences containing the modified adducts in duplexes 5´ GGCTXGATCACCAG 3´ / 5´ CTGGTGATCAAGCC 3´ where X is dT (—), **DFP** (– – –) or **TPP** (– • –). Solutions containing a total strand concentration of 3.8 μM for the duplexes were prepared in buffer containing 90 mM sodium chloride, pH = 7.0, 10 mM sodium phosphate, and 1 mM EDTA buffer.

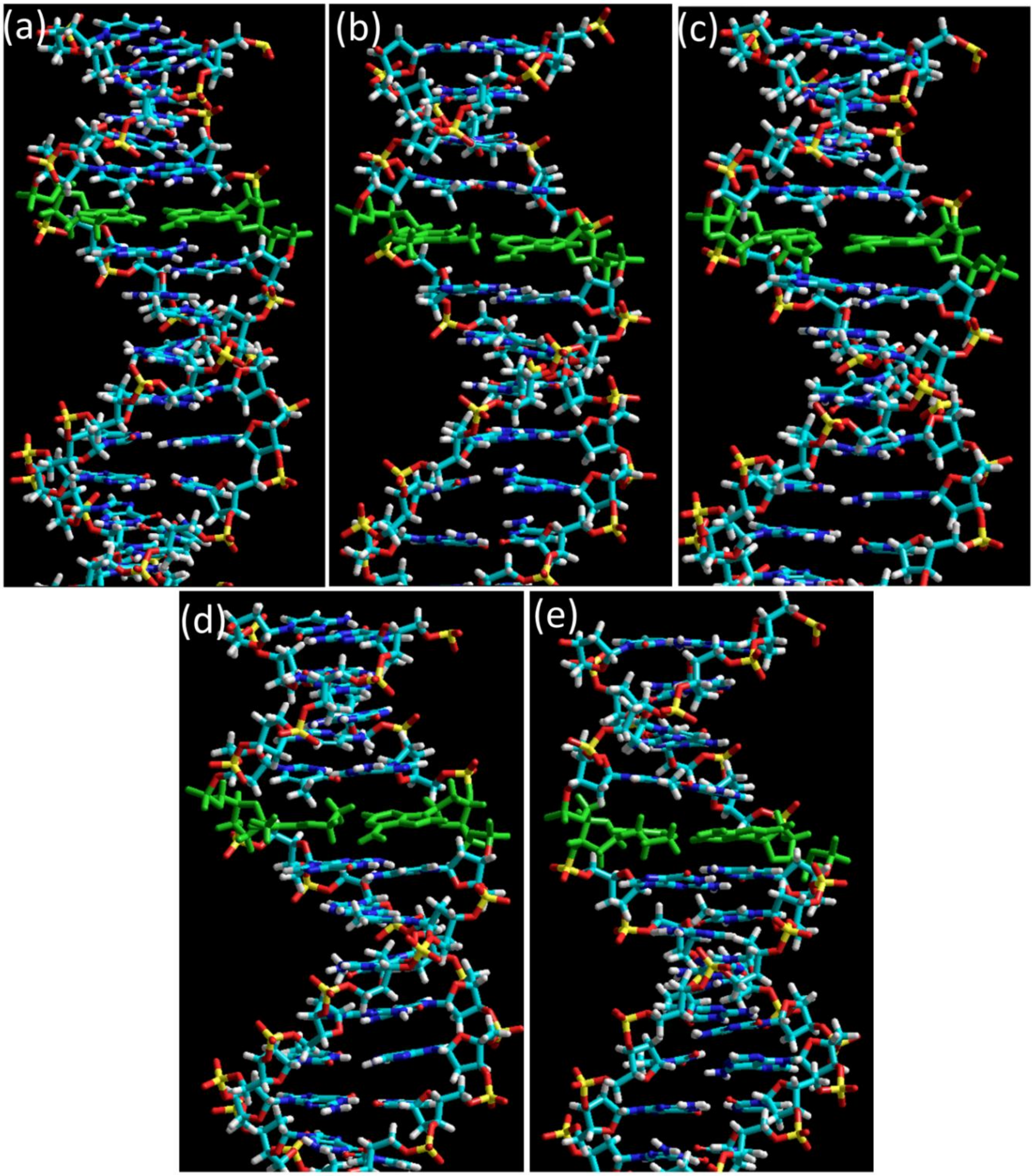

Supplementary Figure S40 - Side view of geometry optimized models of DNA duplexes 5' GGCTXGATCACCAG 3' / 5' CTGGTGATCAAGCC 3' where X is (a) dT, (b) O^4MedT, (c) **DFP**, (d) O^4EtdT, and (e) **TPP**.

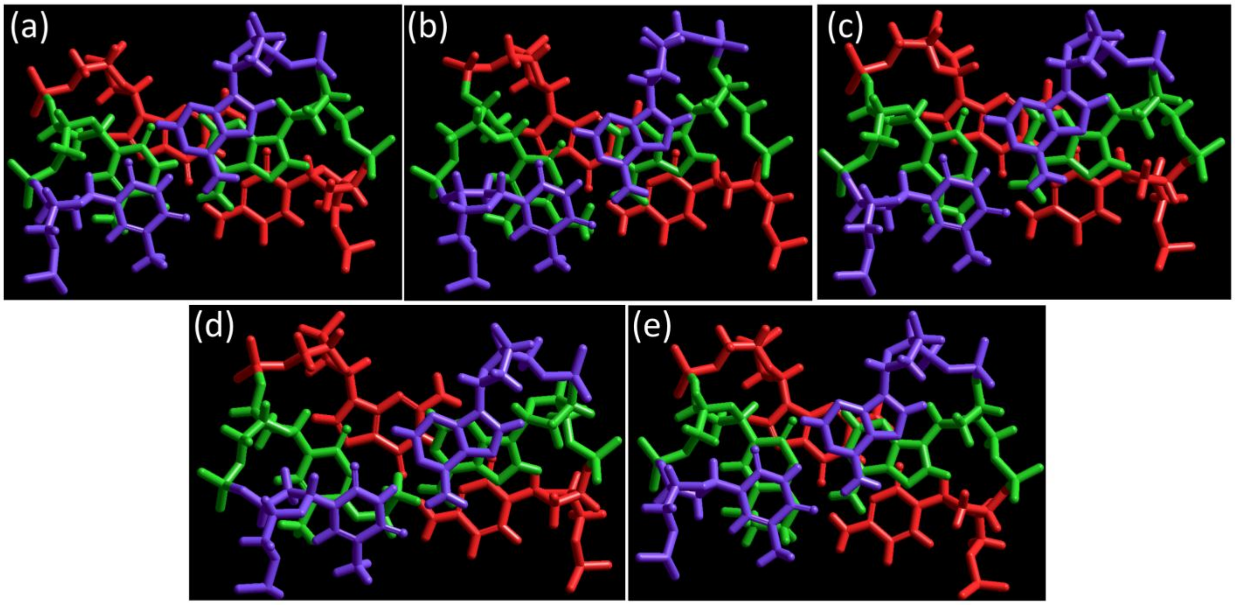

Supplementary Figure S41 - Top view of geometry optimized models of the DNA duplexes 5’ GGCTXGATCACCAG 3’ / 5’ CTGGTGATCAAGCC 3’ where X is (a) dT, (b) *O*^{4}MedT, (c) **DFP**, (d) *O*^{4}EtdT, and (e) **TPP**. All X · A base pairs are colored in green and flanking base pairs are red and purple. The modified thymidyl nucleotides were found to adopt a wobble conformation similar to those described experimentally by NMR and computationally for *O*^{4}MedT and *O*^{4}EtdT.

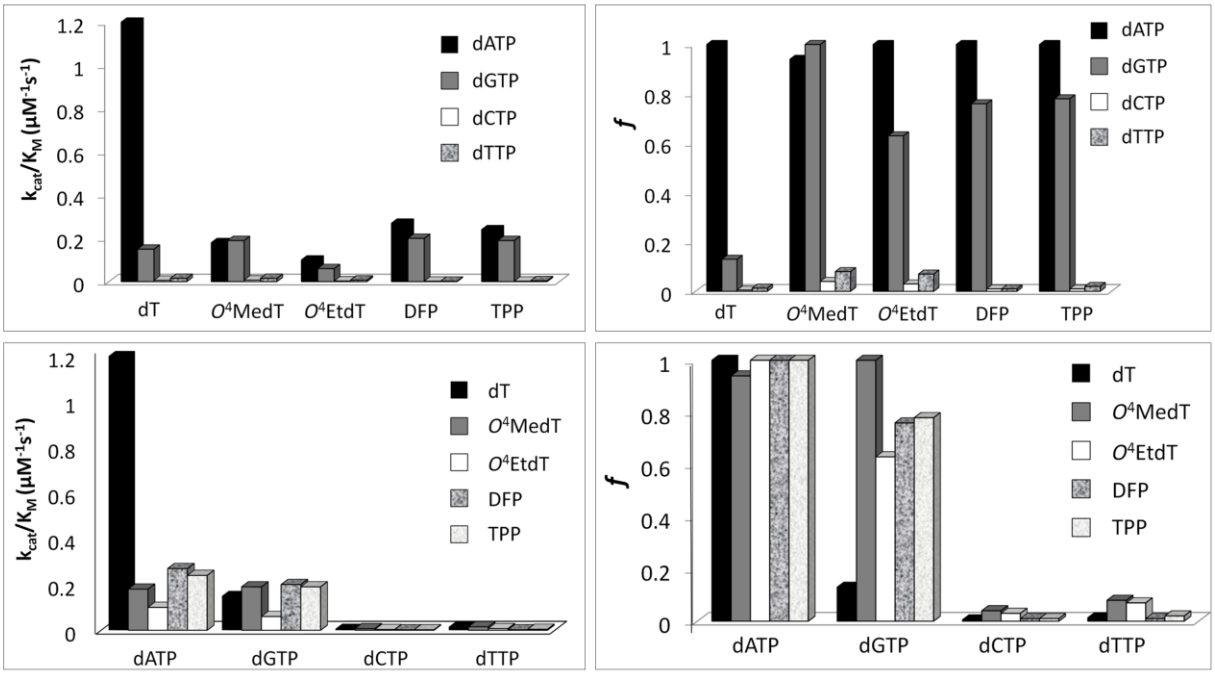

Supplementary Figure S42 - Steady-state incorporation efficiencies opposite dT, O^4MedT, O^4EtdT, DFP, and TPP by hPol η with individual dNTPs. Values are reported in **Supplementary Table S1.** Panels (a-d) are different representations of steady-state kinetic data. Note that Panel (a) is re-depicted here, despite being shown in the main text, for ease of comparison with panels (b-d).

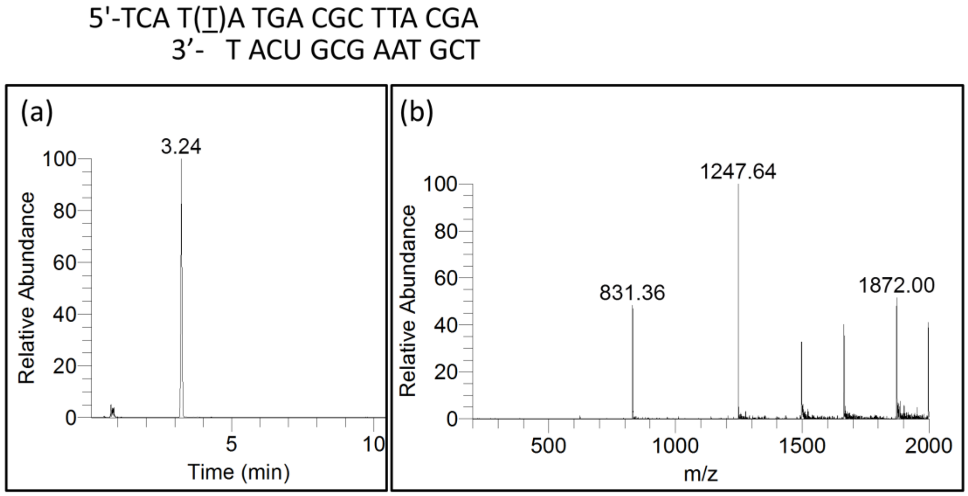

Supplementary Figure S43 - LC-MS analysis of the most abundant full-length extension products opposite unmodified dT in the DNA template by hPol *η* in the presence of all four dNTPs. (a) Sample reconstructed extracted ion chromatogram for *m/z* 1247.8 for product with sequence 5'-pCAT AATGA and (b) mass spectrum of peak at retention time 3.24 min. See **Supplementary Table S2** for the full list of products and respective *m/z* assignments.

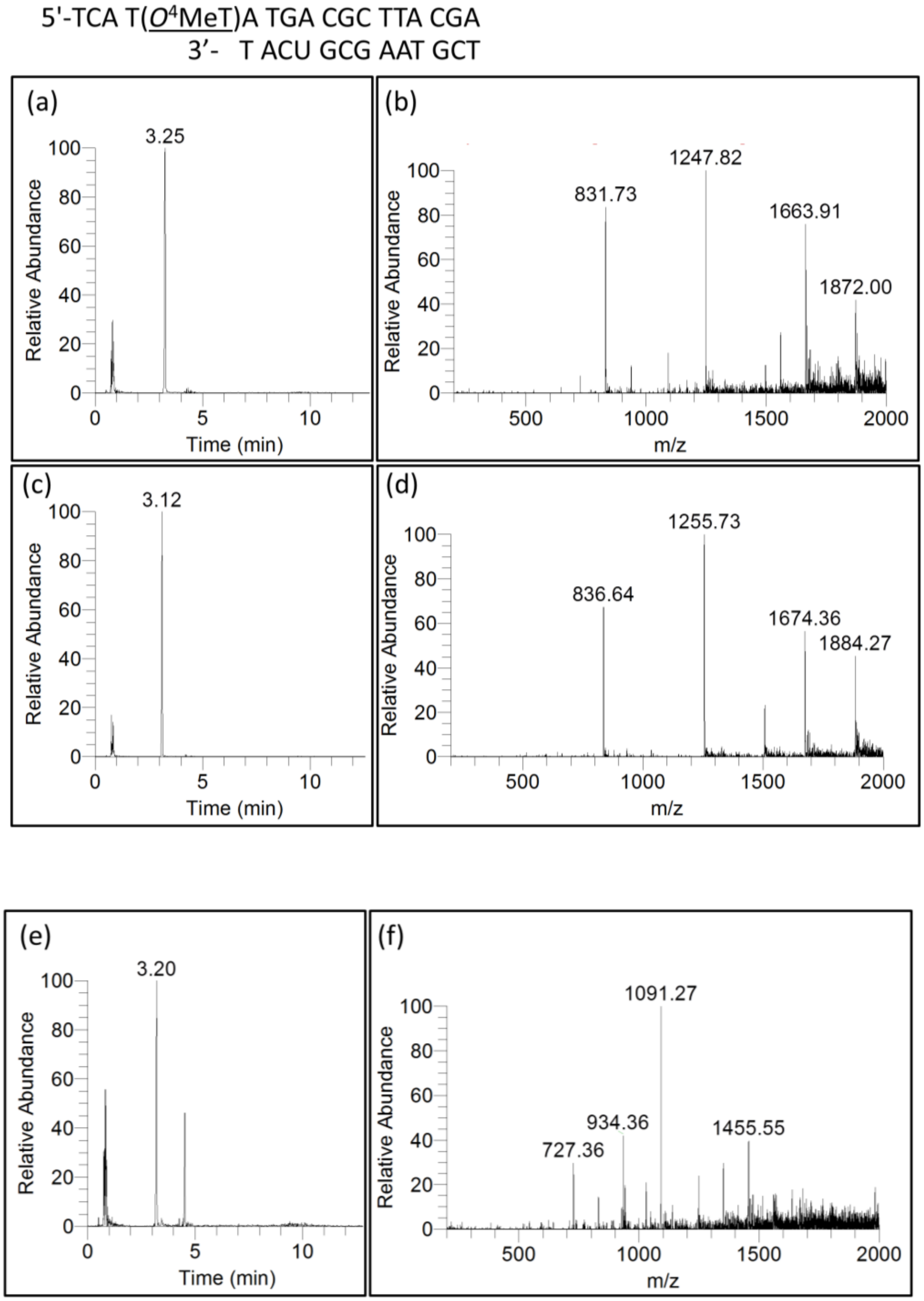

Supplementary Figure S44 - LC-MS analysis of the most abundant full-length extension products opposite O^4MedT in the DNA template by hPol η in the presence of all four dNTPs. (a) Sample reconstructed extracted ion chromatogram for *m/z* 1247.8 for product with sequence 5'-pCAT AATGA and (b) mass spectrum of peak at retention time 3.25 min. (c) Sample reconstructed extracted ion chromatogram for *m/z* 1255.8 for product with sequence 5'-pCAT GATGA and (d) mass spectrum of peak at retention time 3.12 min. (e) Sample reconstructed

extracted ion chromatogram for *m/z* 1091.2 for product with sequence 5’-pCAT _ATGA and (f) mass spectrum of peak at retention time 3.20 min. See **Supplementary Table S2** for the full list of products and respective *m/z* assignments.

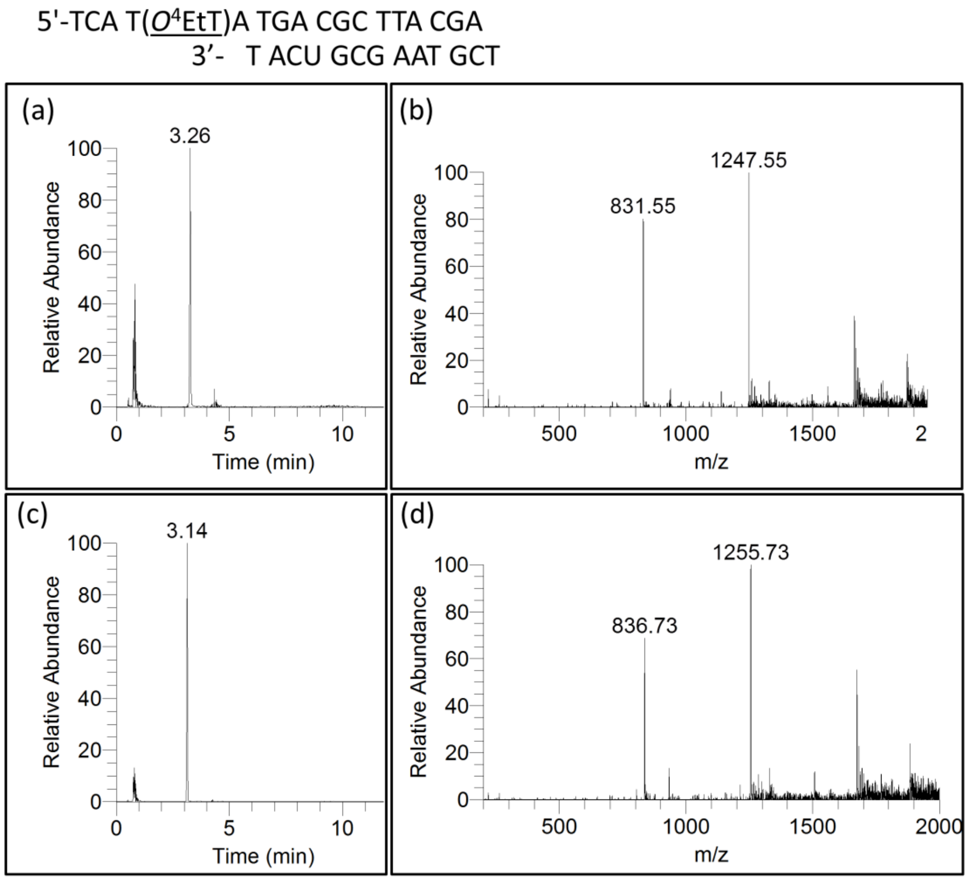

Supplementary Figure S45 - LC-MS analysis of the most abundant full-length extension products opposite O^4EtdT in the DNA template by hPol η in the presence of all four dNTPs. (a) Sample reconstructed extracted ion chromatogram for *m/z* 1247.8 for product with sequence 5’-pCAT AATGA and (b) mass spectrum of peak at retention time 3.26 min. (c) Sample reconstructed extracted ion chromatogram for *m/z* 1255.8 for product with sequence 5’-pCAT GATGA and (d) mass spectrum of peak at retention time 3.14 min. See **Supplementary Table S2** for the full list of products and respective *m/z* assignments.

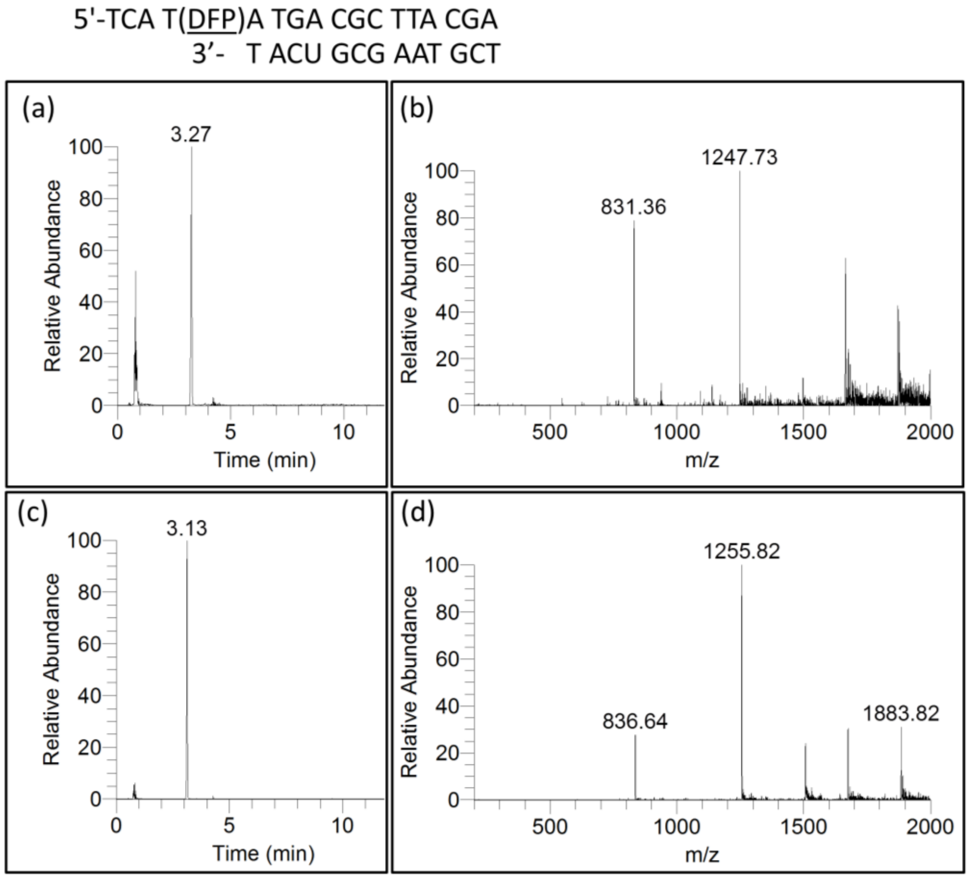

Supplementary Figure S46 - LC-MS analysis of the most abundant full-length extension products opposite DFP in the DNA template by hPol η in the presence of all four dNTPs. (a) Sample reconstructed extracted ion chromatogram for *m/z* 1247.8 for product with sequence 5'-pCAT AATGA and (b) mass spectrum of peak at retention time 3.27 min. (c) Sample reconstructed extracted ion chromatogram for *m/z* 1255.8 for product with sequence 5'-pCAT GATGA and (d) mass spectrum of peak at retention time 3.13 min. See **Supplementary Table S2** for the full list of products and respective *m/z* assignments.

.

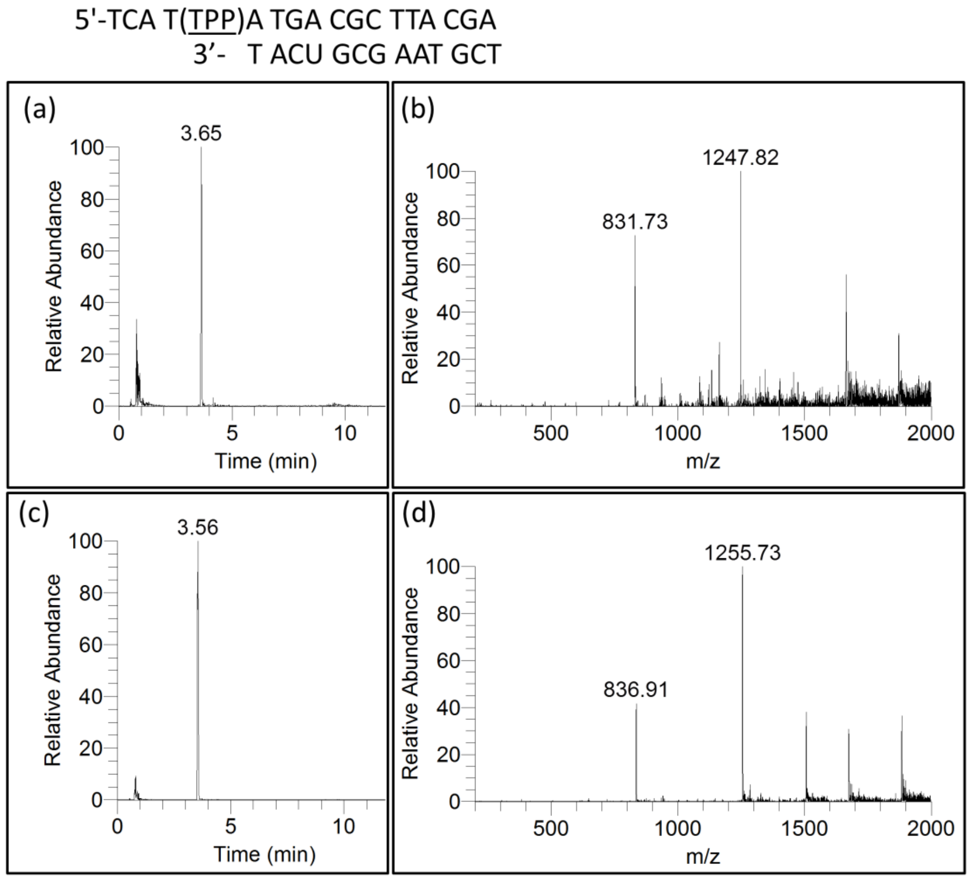

Supplementary Figure S47 - LC-MS analysis of the most abundant full-length extension products opposite TPP in the DNA template by hPol η in the presence of all four dNTPs. (a) Sample reconstructed extracted ion chromatogram for *m/z* 1247.8 for product with sequence 5'-pCAT AATGA and (b) mass spectrum of peak at retention time 3.65 min. (c) Sample reconstructed extracted ion chromatogram for *m/z* 1255.8 for product with sequence 5'-pCAT GATGA and (d) mass spectrum of peak at retention time 3.56 min. See **Supplementary Table S2** for the full list of products and respective *m/z* assignments.

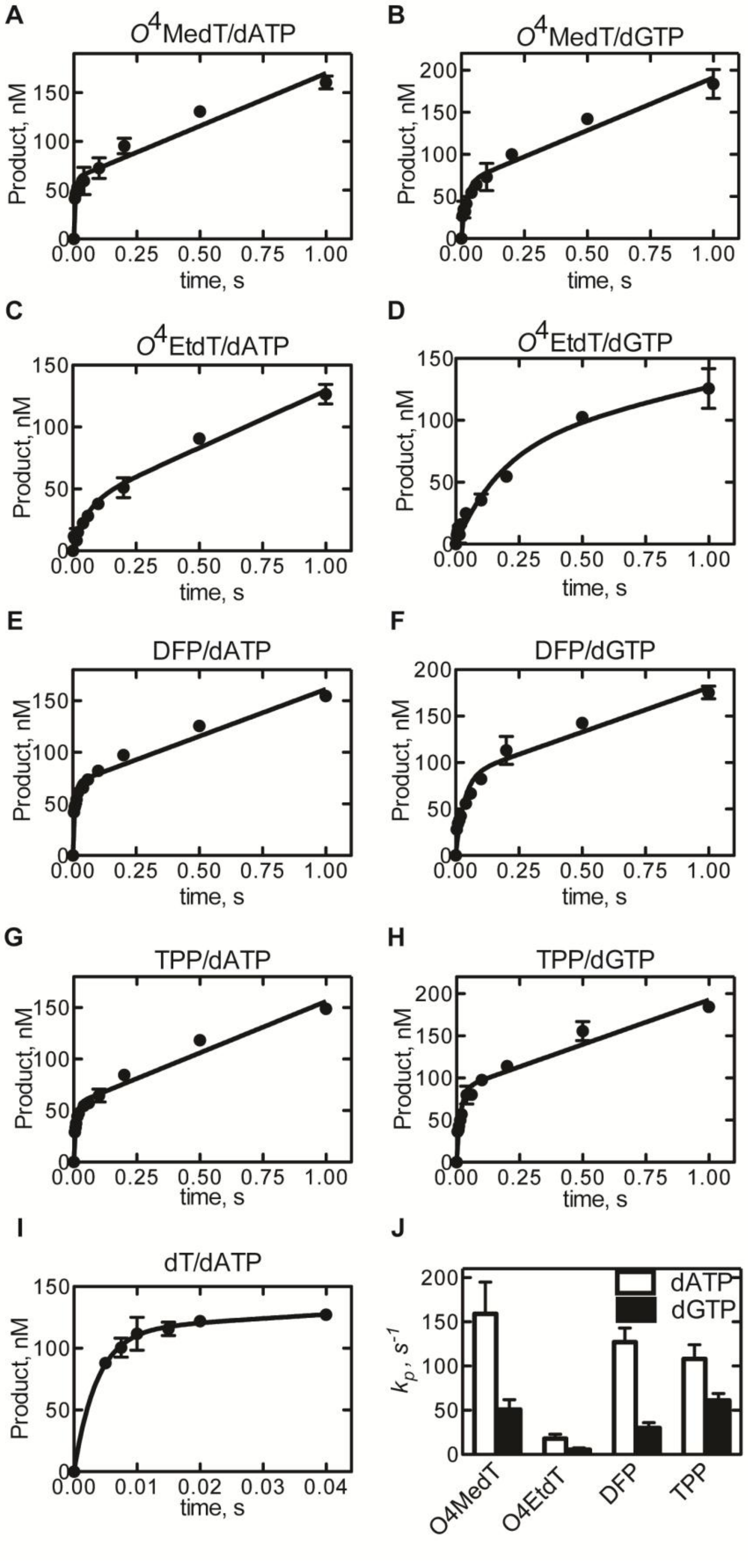
A
O4MedT/dATP
B
O4MedT/dGTP
C
O4EtdT/dATP
D
O4EtdT/dGTP
E
DFP/dATP
F
DFP/dGTP
G
TPP/dATP
H
TPP/dGTP
I
dT/dATP
J
Product, nM
time, s
0.00 0.25 0.50 0.75 1.00
0.00 0.01 0.02 0.03 0.04
0 50 100 150 200
k_p, s^{-1}
dATP
dGTP
O4MedT
O4EtdT
DFP
TPP

Supplementary Figure S48 - Pre-steady-state kinetic analysis of dNTP incorporation by hPol *η* at 37 °C. (A) Template *O*^{4}MedT and incoming dATP. (B) Template *O*^{4}MedT and incoming dGTP. (C) Template *O*^{4}EtdT and incoming dATP. (D) Template *O*^{4}EtdT and incoming dGTP. (E) Template DFP and incoming dATP. (F) Template DFP and incoming dGTP. (G) Template TPP and incoming dATP. (H) Template TPP and incoming dGTP. (I) Template dT and incoming dATP. (J) Comparison of k_p for each reaction. DNA sequences were as follows; 5´-FAM-labeled primer (5´-TCGTAAGCGUCAT-3´) and template (3´-AGCATTCGCAGTAXTACT-5´), where X denotes the modified nucleotide.[14]

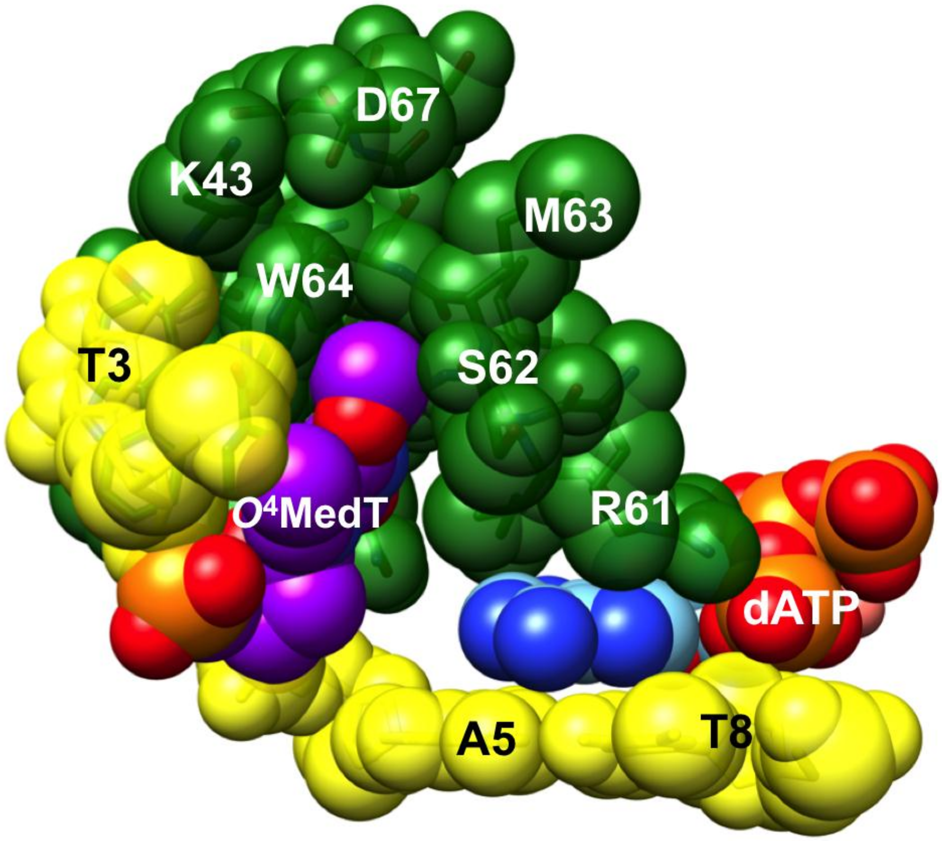

Supplementary Figure S49 - Space filling model of the hPol η active site with O^4MedT (magenta carbon atoms) wedged between Trp-64 and Ser-62 (protein residues are green), allowing for extensive hydrophobic interactions between the O^4MedT methyl group and surrounding residues from the polymerase finger domain. Carbon atoms of the incoming dATP are colored in light blue and template dT 3′ to the lesion and the 5′-adjacent T:A base pair are colored in yellow.

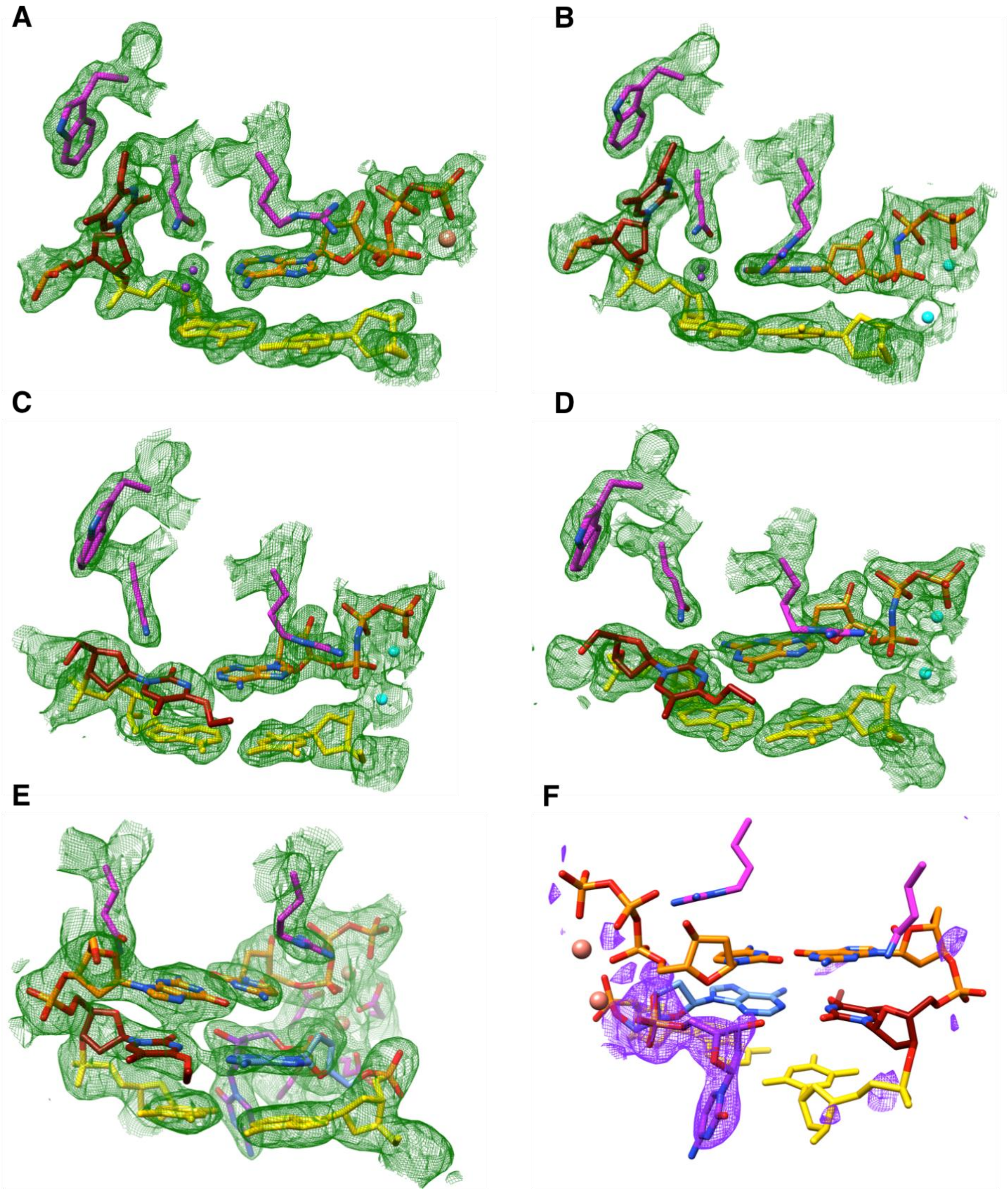

Supplementary Figure S50 - Quality of the final models of ternary hPol η complexes with O^4MedT/O^4EtdT-adducted DNA template strands. Fourier (2F*o*-F*c*) sum electron density drawn at the 1σ threshold (green meshwork) around the active site region. *A*, O^4MedT and dATP,

insertion stage. *B*, O^4MedT and dGMPNPP, insertion stage. *C*, O^4EtdT and dAMPNPP, insertion stage. *D*, O^4EtdT and dGMPNPP, insertion stage. *E*, O^4EtdT opposite primer dA, followed by dCTP opposite template dG; extension stage. *F*, Omit (F*o*-F*c*) electron density drawn at the 3σ threshold (purple meshwork) around the 3´-terminal primer dC that was added during crystallization. Carbon atoms of O^4MedT and O^4EtdT, the incoming nucleotide triphosphate, and selected hPol η amino acid side chains are colored in maroon, orange and magenta, respectively, and Mg^{2+} and Ca^{2+} ions are cyan and pink spheres, respectively. Carbon atoms of the template dA paired to O^4EtdT and those of the 3′-terminal primer dC are colored in light blue and purple, respectively.

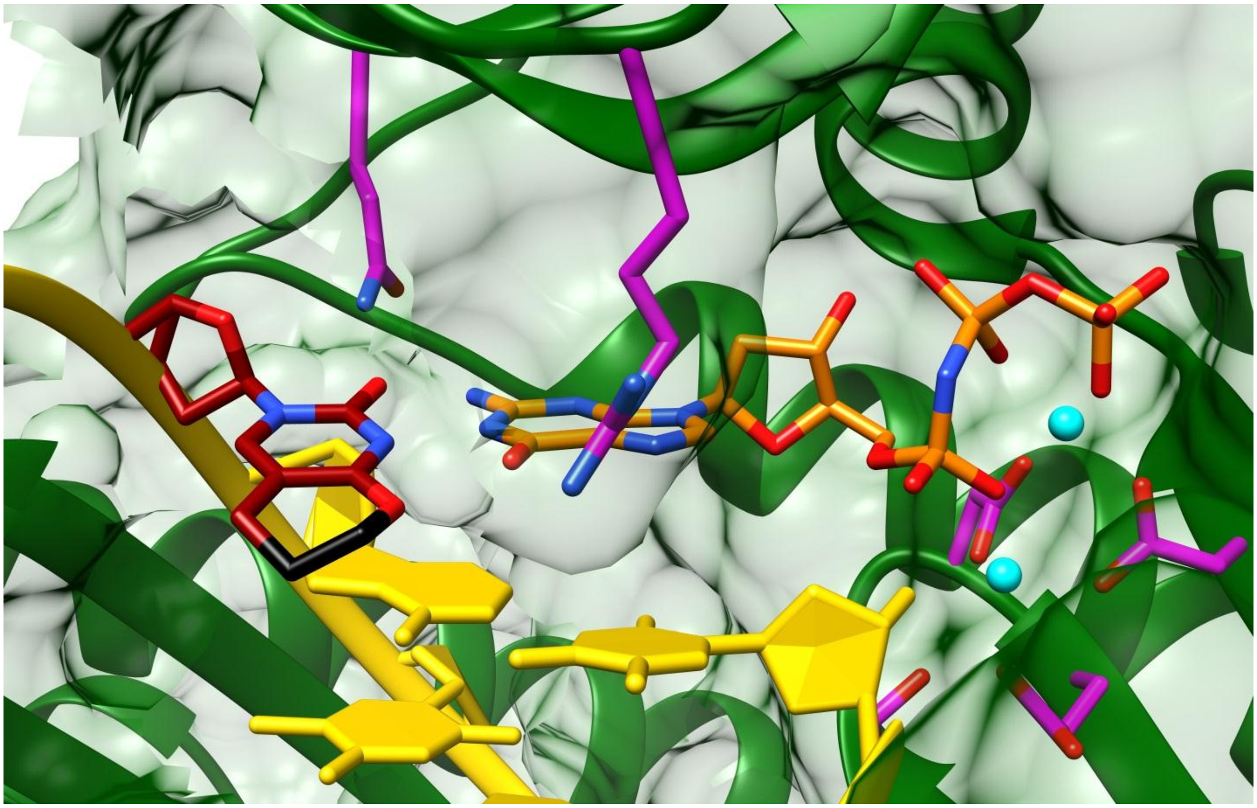

Supplementary Figure S51 - Model of the active site configuration in the ternary hPol η insertion-step complex with dGMPNPP opposite TPP (black carbon atoms). The TPP moiety was modeled in place of the ethyl adduct (O^4EtdT) seen in the crystal structure of the complex. The bicyclic ring protrudes into the major groove of the template-primer duplex, and can be accommodated without clashing with incoming nucleotide triphosphate or amino acid side chains in its vicinity.

Supplementary Table S1 - Steady-state kinetics of incorporation of dATP, dGTP, dCTP and dTTP opposite dT, O^4MedT, O^4EtdT, DFP, and TPP by hPol η.

**Template base**	**dNTP**	k_{cat} (s^{-1})	K_M (μM)	k_{cat}/K_M ($\mu M^{-1}\ s^{-1}$)	f [1]
dT	dATP	1.60 ± 0.03	1.4 ± 0.1	1.2 ± 0.1	
	dGTP	0.71 ± 0.03	4.6 ± 0.7	0.15 ± 0.02	0.13
	dCTP	0.16 ± 0.01	29 ± 7	0.006 ± 0.001	0.005
	dTTP	0.37 ± 0.01	24 ± 3	0.015 ± 0.002	0.013
O^4MedT	dATP	0.69 ± 0.03	3.9 ± 0.6	0.18 ± 0.03	0.94
	dGTP	1.18 ± 0.02	6.2 ± 0.4	0.19 ± 0.01	
	dCTP	0.11 ± 0.01	15 ± 3	0.007 ± 0.002	0.04
	dTTP	0.25 ± 0.02	17 ± 4	0.015 ± 0.004	0.08
O^4EtdT	dATP	0.83 ± 0.03	9 ± 1	0.10 ± 0.01	
	dGTP	0.61 ± 0.02	10 ± 1	0.06 ± 0.01	0.63
	dCTP	0.12 ± 0.01	43 ± 10	0.003 ± 0.001	0.03
	dTTP	0.19 ± 0.02	27 ± 7	0.007 ± 0.002	0.07
DFP	dATP	1.18 ± 0.05	4.4 ± 0.7	0.27 ± 0.04	
	dGTP	1.15 ± 0.02	5.7 ± 0.4	0.20 ± 0.02	0.76
	dCTP	0.12 ± 0.01	55 ± 14	0.002 ± 0.001	0.01
	dTTP	0.20 ± 0.02	84 ± 21	0.002 ± 0.001	0.01
TPP	dATP	1.33 ± 0.04	5.4 ± 0.6	0.24 ± 0.03	
	dGTP	1.18 ± 0.03	6.2 ± 0.6	0.19 ± 0.02	0.78
	dCTP	0.12 ± 0.01	54 ± 12	0.002 ± 0.001	0.01
	dTTP	0.21 ± 0.01	51 ± 11	0.004 ± 0.001	0.02

[1] $f = (k_{cat}/K_M)_{dNTP} / (k_{cat}/K_M)_{max}$

Supplementary Table S2 - LC-MS/MS analysis of full-length extension products opposite dT, O^4MedT, O^4EtdT, DFP and TPP by hPol η (values are reported in %).

**Name**	**Sequence**	**dT**	**O^4MedT**	**O^4EtdT**	**DFP**	**TPP**
7Del	5'-pCAT _ATGA		6	2	< 1	< 1
8A	5'-pCAT AATGA	89	27	22	21	16
8_A	5'-pCAT _ATGAA	< 1	1	< 1	< 1	< 1
8G	5'-pCAT GATGA	3	45	50	69	72
8_G	5'-pCAT _ATGAG	3	3	1		< 1
8C	5'-pCAT CATGA	< 1		< 1		< 1
8T	5'-pCAT TATGA		1	2		
9AA	5'-pCAT AATGAA	3	1	1	< 1	
9AG	5'-pCAT AATGAG	2	1	1	< 1	< 1
9GA	5'-pCAT GATGAA	< 1	8	10	4	4
9GG	5'-pCAT GATGAG		7	10	3	3

Supplementary Table S3 - Pre-steady-state kinetics of incorporation of dATP and dGTP by hPol η opposite O^4MedT, O^4EtdT, DFP, and TPP, and unmodified control (dT).

**Template base**	**dNTP**	**k_p (s^{-1})**	**k_{ss} (s^{-1})**	**Burst amplitude (nM)**
O^4MedT	dATP	159±36	0.43±0.03	62±4
	dGTP	51±11	0.50±0.04	66±5
O^4EtdT [(a)]	dATP	18±5	0.37±0.03	37±5
	dGTP	5.5±2.0	0.19±0.10	80±23
DFP	dATP	127±16	0.36±0.02	70±3
	dGTP	30±6	0.38±0.04	85±7
TPP	dATP	108±16	0.40±0.02	56±3
	dGTP	61±8	0.42±0.03	87±4
dT [(b)]	dATP	>200		

[(a)] It was difficult to observe burst reaction for dATP or dGTP incorporation opposite O^4EtdT.
[(b)] The reaction for dATP incorporation opposite dT was too fast to be detected by the KinTek RP-3 instrument (KinTek Corporation, Austin, TX).

Supplementary Table S4 - Crystal data, data collection parameters and structure refinement statistics.

**Complex**	**O^4MedT: dATP**	**O^4MedT: dGMPNPP**	**O^4EtdT: dAMPNPP**	**O^4EtdT: dGMPNPP**	**O^4EtdT:dA Extension**
**Data Collection**					
Wavelength [Å]	0.97856	0.97856	0.97856	0.97872	0.97872
Space group	$P6_1$	$P6_1$	$P6_1$	$P6_1$	$P6_1$
Resolution [Å]	50 - 1.97 (2.00 - 1.97)[a]	50 - 2.35 (2.39 - 2.35)	50 - 2.29 (2.35 - 2.29)	50 - 1.99 (2.02 - 1.99)	50 - 2.30 (2.34 - 2.30)
Unit cell *a*=*b*, *c* [Å]	99.03, 82.17	98.66, 82.13	98.82, 82.02	98.47, 82.23	98.64, 81.54
Unique reflections	32,497 (1,624)	18,606 (901)	20,600 (1,504)	30,841 (1,534)	20,077 (941)
Completeness [%]	100 (100)	97.9 (96.5)	100 (100)	98.9 (98.5)	99.4 (94.5)
I/σ(I)	14.30 (2.13)	10.46 (1.75)	8.40 (3.20)	19.92 (2.31)	22.72 (2.05)
Wilson B-factor [Å^2]	16.0	30.9	26.1	22.8	43.9
R-merge	0.165 (0.834)	0.127 (0.941)	0.140 (0.539)	0.139 (0.997)	0.093 (0.897)
Redundancy	5.7 (5.7)	4.2 (3.5)	5.6 (5.6)	11.4 (10.0)	7.2 (5.6)
**Refinement**					
R-work	0.165 (0.213)	0.171 (0.238)	0.176 (0.156)	0.161 (0.198)	0.204 (0.289)
R-free	0.216 (0.282)	0.247 (0.301)	0.227 (0.261)	0.210 (0.274)	0.266 (0.363)
Number of atoms Protein/DNA	3,402/384	3,366/387	3,367/389	3,399/394	3,323/410

dNMPNPP/ Mg^{2+}	30/1 (Ca^{2+})	31/2	30/2	31/2	28/2 (Ca^{2+})
Water/Solute	494/1	222/1	212/1	362/1	103/0
Protein residues	439	430	430	438	424
B-factor [$Å^2$]					
Average	24.0	34.0	31.0	28.0	55.5
Protein/DNA	23.2/26.5	34.2/39.8	30.9/36.9	27.0/34.2	55.3/60.1
dNMPNPP/ Mg^{2+}	20.5/12.8	28.3/28.6	19.3/17.8	23.6/18.9	39.3/41.6
Water/ glycerol	30.4/13.4	35.2/27.1	30.1/20.9	32.5/ 19.5	47.9/-
R.m.s. deviations					
bonds [Å]	0.016	0.009	0.012	0.008	0.011
angles [deg.]	1.6	1.1	1.4	1.1	1.5
Ramachandran					
Favored (%)	98	97	97	98	96
Allowed (%)	1.5	2.3	2.8	1.8	3.0
Outliers (%)	0.5	0.7	0.2	0.2	1.0
PDB ID Code	5DLF	5DLG	5DQG	5DQH	5DQI

[a] Statistics for the highest-resolution shell are shown in parentheses.

References

(1) Fissekis, J. D.; Myles, A.; Bosworth Brown, G. *J. Org. Chem.* **1964**, *29*, 2670.
(2) Gazivoda, T.; Raic-Malic, S.; Hergold-Brundic, A.; Cetina, M. *Molecules* **2008**, *13*, 2786.
(3) Denny, G. H.; Ryder, M. A. *J. Med. Chem. Soc.* **1974**, *17*, 1230.
(4) Leonard, N. J.; Cundall, R. L. *J. Am. Chem. Soc.* **1974**, *96*, 5904.
(5) Fissekis, J. D.; Sweet, F. *J. Org. Chem.* **1973**, *38*, 264.
(6) Puglisi, J. D.; Tinoco, I., Jr. *Methods Enzymol.* **1989**, *180*, 304.
(7) McManus, F. P.; O'Flaherty, D. K.; Noronha, A. M.; Wilds, C. J. *Org. Biomol. Chem.* **2012**, *10*, 7078.
(8) Lowe, L. G.; Guengerich, F. P. *Biochemistry* **1996**, *35*, 9840.
(9) Patra, A.; Nagy, L. D.; Zhang, Q.; Su, Y.; Muller, L.; Guengerich, F. P.; Egli, M. *J. Biol. Chem.* **2014**, *289*, 16867.
(10) Chowdhury, G.; Guengerich, F. P. *Current Protocols Nucleic Acid Chem. / edited by Serge L. Beaucage* **2011**, *Chapter 7*, Unit 7 16 1.
(11) Christov, P. P.; Angel, K. C.; Guengerich, F. P.; Rizzo, C. J. *Chemical Res. Toxicol.* **2009**, *22*, 1086.
(12) Zang, H.; Goodenough, A. K.; Choi, J. Y.; Irimia, A.; Loukachevitch, L. V.; Kozekov, I. D.; Angel, K. C.; Rizzo, C. J.; Egli, M.; Guengerich, F. P. *J. Biol. Chem.* **2005**, *280*, 29750.
(13) Patra, A.; Zhang, Q.; Lei, L.; Su, Y.; Egli, M.; Guengerich, F. P. *J. Biol. Chem.* **2015**, *290*, 8028.
(14) Su, Y.; Patra, A.; Harp, J. M.; Egli, M.; Guengerich, F. P. *J. Biol. Chem.* **2015**, *290*, 15921.
(15) Otwinowski, Z.; Minor, W. *Methods Enzymol.* **1997**, *276*, 307.
(16) *Acta Crystallogr. Section D, Biol. Crystallogr.* **1994**, *50*, 760.
(17) McCoy, A. J.; Grosse-Kunstleve, R. W.; Adams, P. D.; Winn, M. D.; Storoni, L. C.; Read, R. J. *J. App. Crystallogr.* **2007**, *40*, 658.
(18) Adams, P. D.; Afonine, P. V.; Bunkoczi, G.; Chen, V. B.; Davis, I. W.; Echols, N.; Headd, J. J.; Hung, L. W.; Kapral, G. J.; Grosse-Kunstleve, R. W.; McCoy, A. J.; Moriarty, N. W.; Oeffner, R.; Read, R. J.; Richardson, D. C.; Richardson, J. S.; Terwilliger, T. C.; Zwart, P. H. *Acta Crystallogr. Section D, Biol. Crystallogr.* **2010**, *66*, 213.
(19) Murshudov, G. N.; Skubak, P.; Lebedev, A. A.; Pannu, N. S.; Steiner, R. A.; Nicholls, R. A.; Winn, M. D.; Long, F.; Vagin, A. A. *Acta Crystallogr. Section D, Biol. Crystallogr.* **2011**, *67*, 355.
(20) Emsley, P.; Cowtan, K. *Acta Crystallogr. Section D, Biol. Crystallogr.* **2004**, *60*, 2126.
(21) Pettersen, E. F.; Goddard, T. D.; Huang, C. C.; Couch, G. S.; Greenblatt, D. M.; Meng, E. C.; Ferrin, T. E. *J. Comput. Chem.* **2004**, *25*, 1605.
(22) Xu, Y. Z.; Swann, P. F. *Nucleic Acids Res.* **1990**, *18*, 4061.
(23) Eger, K.; Jalalian, M.; Schmidt, M. *J. Heterocyclic Chem.* **1995**, *32*, 211.
(24) Zhu, Q.; Delaney, M. O.; Greenberg, M. M. *Bioorg. Med. Chem. Lett.* **2001**, *11*, 1105.
(25) Fabrega, C.; Eritja, R.; Sinha, N. D.; Dosanjh, M. K.; Singer, B. *Bioorg Med. Chem.* **1995**, *3*, 101.
(26) Xu, Y. Z.; Swann, P. F. *Tetrahed. Lett.* **1994**, *35*, 303.
(27) Kalnik, M. W.; Kouchakdjian, M.; Li, B. F.; Swann, P. F.; Patel, D. J. *Biochemistry* **1988**, *27*, 100.
(28) Cruzeiro-Hansson, L.; Goodfellow, J. M. *Carcinogenesis* **1994**, *15*, 1525.
(29) Li, B. F.; Reese, C. B.; Swann, P. F. *Biochemistry* **1987**, *26*, 1086.
